# Analysis of Fall and Jump Behaviors in Freely Moving *Drosophila melanogaster* Using 58 fps Video

**DOI:** 10.3390/insects17060624

**Published:** 2026-06-13

**Authors:** Shoham Das, Yash Patel, Kyle Wang, John Tower

**Affiliations:** Molecular and Cellular Biosciences Department, University of Southern California, Los Angeles, CA 90089, USA; shohamda@usc.edu (S.D.); yashdhir@usc.edu (Y.P.); kjiayiwang@gmail.com (K.W.)

**Keywords:** *Drosophila*, aging, falls, video, machine learning, locomotor activity, computer vision

## Abstract

Analysis of freely moving *Drosophila* captures complex movement behaviors. However, previous experiments have been limited by the inability to distinguish between falls and downward jumps (downjumps). Here, individual flies moving freely in a culture vial were recorded using a single 58 fps video camera. Upward jumps were readily identified by positive movement in the vertical direction. Several statistical and machine learning methods were used to distinguish between falls and downjumps, including Principal Component Analysis (PCA), K-Means Clustering, Uniform Manifold Approximation and Projection (UMAP), Hierarchical Density-Based Spatial Clustering with Applications to Noise (HDBSCAN) and Shapley Additive Explanations (SHAP). Falls were abundant and characterized by an initial velocity consistent with simple acceleration due to gravity. Downjumps were more rare, and were characterized by a greater initial velocity, indicating active propulsion by the fly. Aged flies took longer to resume movement after a fall, suggesting possible negative effects of falls. Falls in young *w*[1118]-strain flies exhibited mid-event velocities that were lower than expected, indicating some compensatory behavior that was reduced in aged flies. These methods should facilitate future studies of the effects of aging and neurodegenerative disease models on locomotor behaviors and falls, including the testing of potential interventions.

## Simple Summary

Falls are a major source of human disability and death, with interventions currently limited to exercise. *Drosophila* is a powerful model system for the analysis of movement disorders and possible interventions, both during normal aging and in *Drosophila* models of human neurodegenerative disease. However, previous *Drosophila* experiments have been limited by the inability to distinguish between falls and downward jumps (downjumps). Here, individual flies moving freely in a culture vial were analyzed using a single 58 fps video camera. Upward jumps were readily identified by positive movement in the vertical direction. Advanced statistical and machine learning tools were used to distinguish between falls and downjumps. Falls were characterized by an initial velocity consistent with simple acceleration due to gravity. By contrast, downjumps were characterized by a greater initial velocity, consistent with active propulsion by the fly. In agreement with previous studies, aged flies moved less and explored a smaller region of the vial. In addition, aged flies took longer to resume movement after a fall, suggesting possible negative effects of falls. These methods should facilitate future studies of the effects of aging and neurodegeneration models on locomotor behaviors and falls, including the testing of potential interventions.

## 1. Introduction

In humans, both normal aging and neurodegenerative disease (ND) are associated with locomotor impairments, including increased falls. Falls are a significant cause of disability and death, and there are currently no effective interventions known other than exercise [1,2,3,4]. It is therefore of interest to leverage the *Drosophila* model system to study the etiology of locomotor impairments, including falls, with the ultimate goal of identifying environmental or small-molecule interventions that might be relevant to human health.

*Drosophila* is a leading model system for the study of nervous system function and animal behavior. The neuronal wiring diagram and cell connectome have been mapped for the entire *Drosophila* brain [5,6], and powerful transgenic tools enable modulation of the activity of specific neuronal subsets, including individual neurons [7]. Video analysis has been used to analyze several complex *Drosophila* behaviors including flight, grooming, mating and aggression [8,9,10,11].

*Drosophila* is also a leading model for the study of aging [12,13,14] and for modeling human ND, including Alzheimer’s disease (AD) and Parkinson’s disease (PD) [15]. Several methods have been used to study how normal aging and the ND models affect *Drosophila* locomotor behaviors. *Drosophila* activity monitors (DAMs) and locomotor activity monitors (LAMs) employ infrared beams in the fly container, and record each time the beam is disrupted by a moving fly. These assays provide quantification of total fly movement activity, which is observed to be reduced with age and in the ND models. The negative geotaxis assay specifically measures upward climbing speed after the fly is knocked to the bottom of a cylinder, a behavior generally interpreted as an induced escape response [16]. Negative geotaxis behavior is also reduced with age and in *Drosophila* ND models [17,18,19]. *Drosophila* have also been assayed for 2D locomotor behaviors while moving freely in a Petri dish-shaped arena [20], and for 3D locomotor behaviors while moving freely in a culture vial [18,21], including assays of 3D movement trajectories using synchronized video cameras [22]. These studies have revealed locomotor behaviors associated with imminent mortality, including bouts of erratic movement (as indicated by increased frequency of directional heading changes), and supine behavior (fly lying on its back) [21,22,23,24,25].

Several previous studies of *Drosophila* have described events where the fly drops rapidly through space from a higher position in the observation chamber to a lower position, and these drops have sometimes been referred to as “falls” depending on the context in which they were observed. For example, Lobato et al. [26] used 30 fps video to analyze flies as they climbed upwards in a negative geotaxis assay. Flies were observed to occasionally drop during climbing, and they defined drops of greater than 11.7 mm as “falls”. Similarly, Canic et al. [27] used 30 fps video to analyze flies as they climbed upwards in the negative geotaxis assay, and defined drops of greater than 12 mm as “falls”. Finally, in our recent study, YOLOv4 machine learning software was trained to recognize drops in 30 fps videos of freely moving flies [28]. In that study, the frequency of drops was found to peak proximal to death associated with normal aging or as produced by dehydration/starvation stress. Those events were interpreted to most likely represent “falls”, because they increased in the hours before death when total movement activity had significantly decreased. One limitation of the previous studies is that 30 fps video analysis cannot unambiguously distinguish between a true fall, where the downwards velocity results only from acceleration due to gravity, and a downjump, where the downwards velocity will include additional acceleration produced by the fly.

Analysis of falls and downjumps is of particular interest, because the inability of aged flies to right themselves after a fall is implicated as one cause of death [22,23,24,25]. Here a single 58 fps camera was used to record individual flies moving freely in a culture vial. Custom software (MotionDetection version 2.1, MDv2.1) was developed to track fly movement trajectories, and analysis of drop velocities and other metrics revealed distinct behaviors, including falls, downjumps, upward jumps (upjumps) and arced jumps (arcjumps).

## 2. Materials and Methods

### 2.1. Drosophila Culture and Strains

*Drosophila melanogaster* flies were cultured at 25 °C and 65–80% humidity under a 12 h:12 h light–dark cycle using standard agar/dextrose/corn meal/yeast media [29]. Several strains were obtained from the Bloomington Drosophila Stock Center (BDSC, Bloomington, IN, USA). Strain *y*[1] *w*[1118]; *P[w[*+*mC]* = *UAS-APP.Abeta42.E693G.VTR]4* (BDSC #33773, abbreviated here as “UAS-Abeta42”, is a UAS-controlled transgene containing the human amyloid β42 protein with the E693G mutation [18]. Strain w[*]; *P[w[*+*mC]* = *UAS-Hsap\SNCA.A30P]40.1* (BDSC #8147, abbreviated here as “UAS-SNCA”) is a UAS-controlled transgene encoding the A30P mutant of human α-synuclein [30]. Strain *P[w[*+*mW.hs]* = *GawB]elav[C155]* (BDSC #458, abbreviated here as “elav-GAL4”) yields neuronal GAL4 expression driven by the *elav* gene promoter [31]. The *w*[1118] reference strain (*w*[1118]*-iso*; *2-iso*; *3-iso*) has been previously described and sequenced [32,33]. This strain was previously cleared of *Wolbachia* by three generations culture on doxycycline followed by confirmation using PCR as described [34]. Each transgenic strain was backcrossed nine generations to the *w*[1118] reference strain. To generate flies with pan-neuronal expression of human α-synuclein or amyloid-β42, virgin females from the elav-GAL4 strain were crossed to UAS-SNCA males or UAS-Abeta42 males, respectively, to generate hybrid progeny. Control flies were produced by crossing elav-GAL4 virgin females to males from the *w*[1118] reference strain. Dehydration/starvation stress was produced by putting young (2-day-old) *w*[1118] male flies in an empty vial for 24 h; control flies were maintained on normal media.

### 2.2. Hardware Implementation

#### 2.2.1. Cameras and Lens

Videos were captured using a FLIR Blackfly S BFS-U3-63S4M-C camera (6.3 megapixels, Sony IMX178 sensor; FLIR Systems, Wilsonville, OR, USA) attached to an 8 mm megapixel fixed-focal-length lens (Edmund Optics, Barrington, NJ, USA). The camera lens was positioned 14.4 cm from the center of the assay vial and aligned directly towards the center of the vial.

#### 2.2.2. Lights and Organization

Illumination was supplied by three external dimmable white LED light sources (power: 1–1.5 W; luminosity: 90–120 lm; color temperature: 3000 K; beam angle: 30°; housing: aluminum, YaeCCC Company, Shanghai, China, Amazon.com). One light source was positioned 10.8 cm directly above the center of the vial. The second light source was located 10.6 cm to the left and 4.4 cm above the center of the camera lens, and 11.8 cm from the vial. The third light source, located to the right of the camera, was placed 12.7 cm to the right and 3.7 cm above the center of the camera lens, and 12.2 cm from the vial.

The light sources were connected via a junction box and simultaneously controlled through a single-channel pulse-width modulation controller. The cameras were connected to computers via USB 3.1 (USB-A to USB Micro-B cable). Video acquisition was performed on HP desktop computers running Windows 10 Enterprise LTSC with dual Intel Xeon E5-2630 processors (2.30 GHz/Core) and 12 GB of RAM.

#### 2.2.3. Video Recording

Fly locomotion assays were conducted in a dark box housing the fixed-camera, fixed-lighting recording setup outlined above. Flies were randomly selected from their respective cohorts, anesthetized through minimal CO_2_ gas exposure, and placed into individual vials with media the day before recording. For assay, flies were transferred without anesthesia from their culture vials into unthreaded 4-dram (~15 mL) glass assay vials containing a black foam plug at the bottom of the vial. The vial is 2 cm inner diameter and 6 cm in height. The top of the foam plug was 2.3 cm from the base of the vial, producing a height of 3.7 cm available to the fly. A 30 mm diameter round glass coverslip (#1.5 thickness, Bioptech, Butler, PA, USA) was placed on top of the assay vial to enclose the fly. The assay vial with coverslip and fly were placed into the recording chamber, and video was recorded for 30 min at 58 frames per second (fps) using SpinView software (version 4.2.0.88, FLIR Systems) for camera control and data acquisition. All videos were recorded between 10:00 AM and 2:00 PM. Videos were analyzed for movement and event detection using custom software Motion DetectionV2.1 (MDv2.1), using HP desktop computers running Windows 10 Enterprise LTSC with dual Intel Xeon W-2245 processors (3.90 GHz/Core) and 64 GB of RAM.

### 2.3. Motion Detection Software and Data Analysis

#### 2.3.1. Object Detection

Video metadata was extracted using OpenCV VideoCapture class properties [35]. Background subtraction utilized the Mixture of Gaussian (MOG2) algorithm, which dynamically updates its background representation to adapt to gradual lighting changes [36]. Background subtraction generates a foreground mask, where a change in pixel values between frames indicates motion.

Contours were passed through a minimum area filter with a floor of 20 pixels to prevent filtering of hidden flies. When multiple contours were detected, each contour was compared with the previous frame’s detections using a weighted distance metric, where distance is calculated as follows:

$$distance=\;2\;\times (\Delta x\;+\;\Delta y)\;+\;0.3\times (\Delta w\;+\;\Delta h)\;+\;0.05\times \frac{\Delta area}{1000}$$

Δ*x* is the absolute difference in x-coordinate, Δ*y* is the absolute difference in y-coordinate, Δ*w* is the absolute difference in width, Δ*h* is the absolute height difference, and Δ*area* is the absolute difference in area. The difference in area is divided by 1000 to reduce area differences to similar magnitudes to position differences.

The software processes the video at two different sensitivity thresholds for changes in pixel intensity. The low sensitivity threshold analysis is conducted first to identify fly movement and reduce false positives. The high sensitivity threshold analysis is then conducted to capture fast movements missed in the initial pass. Any missing values in the lower-sensitivity detections that were detected by the higher-sensitivity analysis were added to the detection file.

All detections are recorded in a CSV file with columns for frame number, timestamp(s), bounding box coordinates (x, y, width, height), and pixel area. If no valid detection occurs in a frame, that frame is omitted from the file. For each video, an annotated video with bounding boxes for each frame area is also optionally generated.

#### 2.3.2. Calibration of Pixels to Mm

Pixels were converted to millimeters by utilizing the vial dimensions as a reference. The software prompts the user to draw horizontal and vertical calibration lines on a single video frame. The vial height (37 mm) and the vial width (20 mm) are divided by the length of the vertical line and the length of the horizontal line, respectively, to generate millimeter-to-pixel ratios. These ratios are then used by the software to convert velocities, displacements, and accelerations into SI units.

#### 2.3.3. Displacement and Kinematic Metrics

Vertical and horizontal displacements are calculated as framewise differences. Velocity is computed by dividing the Euclidean distance by the time difference between frames. The equation is as follows:

$$velocity\;=\;\frac{\sqrt{{\Delta x}^{2}+{\Delta y}^{2}}}{\Delta t}$$

Event-level and frame-by-frame kinematic metrics derived from these calculations are summarized in Table S1. Pixels were converted to millimeters using the calibration method outlined above to convert kinematic metrics into SI units. All velocities and horizontal displacements are expressed as absolute values in figures and tables.

#### 2.3.4. Event Detection, Classification, and Filtering

Candidate events are initially identified by vertical displacement thresholds. Downward events (referred to as drops) were identified by vertical displacements exceeding 60 pixels from frame n to frame n + 2 in the downward direction. Upjumps were identified by vertical displacements exceeding 50 pixels from frame n to frame n + 1 in the upward direction. Upjumps followed by a drop within a predefined refractory period of 15 frames (0.26 s) are classified as arcjumps and the drop within the refractory period is not counted.

To account for errors in object tracking introduced by any glare at the top of the vial, events occurring entirely within the upper 25% of the vial were censored. Consecutive events within a predefined refractory period of 15 frames are merged to avoid double-counting.

#### 2.3.5. Defining the Temporal Bounds of Events

Event triggers are the metrics by which the software identifies an event, and are calculated by the first instance the vertical displacement exceeds an event’s displacement threshold (Table S1). The event start is estimated by taking the time at event trigger (Table S1), and adjusting back to frame n − 1 if frame n − 1 is consecutive and the vertical displacement from frame n to frame n − 1 is greater than 20 pixels. Event ends are determined by subsequent vertical movement stability, defined either by less than 5 pixels of vertical movement from frame n to frame n + 1, or less than 10 pixels of vertical movement from frame n to frame n + 3.

### 2.4. Statistical Analyses

All data processing, statistical analyses, and dimensionality reduction were conducted using Python (Version 3.12.12). Analyses were conducted using custom Python scripts. Data organization, data manipulation, and numerical operations were performed using the Python libraries pandas (Version 2.2.2) and NumPy (Version 2.0.2) [37]. Group comparisons were conducted using the SciPy library (Version 1.16.3). To compare between two independent groups in event count and drop metric assays, two-tailed Student’s *t*-tests were performed. Welch’s correction was applied when assumptions of equal variance were violated, and the Mann–Whitney U test was used when normality assumptions were not met. To compare drop metrics between clusters, the nonparametric Kruskal–Wallis test was used. Post hoc pairwise comparisons were conducted using Dunn’s test using the scikit-posthocs package (Version 0.12.0) to determine significant differences between clusters [38]. Bonferroni correction using the statsmodels package (Version 0.14.6) was used to correct for multiple testing wherever applicable [39]. Statistical significance was defined as *p* < 0.05.

Principal Component Analysis (PCA) was performed through the scikit-learn library (Version 1.6.1) [40]. Numerical data were standardized by centering variables to a zero mean and scaling to unit variance. Centroid-based partitional clustering was performed using the K-Means Clustering algorithm [41] implemented by the scikit-learn Python package. The number of clusters (*k*) was predefined as three. Uniform Manifold Approximation and Projection (UMAP) was performed using the umap-learn package (Version 0.5.11) with default parameters [42]. Student’s *t*-test comparisons, PCA data, and UMAP data were visualized using the libraries Matplotlib (Version 3.10.0) [43] and seaborn (Version 0.13.2) [44]. Density-based clustering was performed using the Hierarchical Density-Based Spatial Clustering of Applications with Noise (HDBSCAN) algorithm [45], implemented by the hdbscan Python package (Version 0.8.41) [46]. The hyperparameter min_cluster_size was optimized by computing the total stability of clusters at each possible min_cluster_size value, closely following the proposed optimization algorithm described by Campello et al. [45]. Candidate min_cluster_size values that produced a noise fraction exceeding 20% of the dataset were excluded from consideration. The optimal value for min_cluster_size was then selected as the value that maximized the total stability of all the clusters. This yielded four individual clusters. All other parameters were kept at their default values. Cluster quality and cohesiveness were examined using the silhouette score, Davies–Bouldin index, and the Calinski–Harabasz index [47,48,49].

To examine the kinematic differences between HDBSCAN-defined clusters, a Random Forest Classifier from the scikit-learn library was trained to predict cluster membership using the entire dataset. Model explanations were generated using Shapley Additive Explanations (SHAP), a method to estimate the contribution of each feature to each prediction. Feature importance was computed using the mean absolute SHAP value across samples in each cluster (Tables S2–S5). The direction of feature importance was quantified using Spearman rank correlation. SHAP analysis was conducted using the SHAP library (Version 0.50.0) [50,51]. One limitation of the SHAP analysis is several of the features were not independent. To address this limitation, an additional SHAP analysis was conducted on a reduced feature set. A representative feature was kept for each of three feature pairs that had a Pearson correlation greater than 0.8. For a group of 4 non-independent features that included maximum velocity, maximum acceleration, maximum jerk and average velocity, a standardized mean was used as a single feature. In addition, two features that did not significantly differ between the HDBSCAN-defined clusters based on the Kruskal–Wallis test were omitted. This reduced the number of features from 18 to 7. This modified SHAP analysis resulted in essentially identical cluster definitions.

### 2.5. Post Hoc Filtration of Event Dataset

Events that were scored as drops but that displayed upward movement were interpreted to result from inaccuracies in fly tracking and were excluded from further analysis, reducing the dataset by 83 drops, or 3.5% of drops. To filter events resulting from non-fly detections (e.g., glare), upjumps with negative vertical displacement were excluded. Arcjumps were modeled using quadratic regression of vertical displacement as a function of time. Arcjumps were excluded if their quadratic correlation coefficient (R^2^) was less than 0.85, the quadratic coefficient was positive, or if the trajectory of the event did not include both positive and negative vertical displacements. These measures further filtered the dataset by 7.32%, reducing the total number of events to 2367. The final dataset consists of 232 videos with 2299 drops, 43 arcjumps, and 25 upjumps.

### 2.6. Velocity Calculations

#### 2.6.1. Expected Initial Velocity for a Fall

The expected initial velocity of resulting from acceleration due to gravity can be calculated using the Newtonian free-fall equation with drag:

$$v\left(t\right)=\;v_{t}tanh(\;\frac{gt}{v_{t}}+\;{tanh}^{-1}\left(\frac{v_{0}}{v_{t}}\right))$$

where $v_{t}$ is the terminal velocity, g is Earth’s gravitational acceleration ($g\approx 9.81\;m/s^{2})$, t is the time of interest, and $v_{0}$ is the initial velocity. Assuming that a fall starts from rest, the equation simplifies to:

$$v\left(t\right)=\;v_{t}tanh(\frac{gt}{v_{t}})$$

MDv2.1 software defines initial velocity as the median of the first two non-zero velocity measurements, which occur between frames 0 and 1, and between frames 1 and 2, in >95% of events. At a video recording speed of 58 frames per second, this time can be represented by the fraction *t_MD_* which is defined as the time at which MDv2.1 calculates initial velocity.

$$t_{MD}=\frac{median(1,2)}{58}=\frac{3}{116}$$

Terminal velocity is calculated as:

$$v_{t}=\;\sqrt{\frac{2W}{\rho AC_{D}}}$$

where W is the weight of the fly, ρ is the density of air, A is the cross-sectional surface area of the fly, and $C_{D}$ is the drag coefficient. The drag coefficient of a de-winged fly was previously estimated as $C_{D}=1$ [52]. The density of air was assumed $\rho =1.2\;kg/m^{3}$, consistent with the air density at sea level. The weight of the fly can be assumed as 10 μΝ [53]. The cross-sectional surface area of the fly can be approximated as an ellipse, with length (l) = 3 mm and an estimated width (w) = 2 mm. Therefore:

$$A=\;\frac{\pi }{4}lw=\;4.71\times 10^{-6}\;m^{2}$$

Substituting these values into the equation above provides a terminal velocity of:

$$v_{t}\approx 1.88\;m/s$$

Revisiting the original equation provides the expected initial velocity:

$$v\left(t\right)=v_{0}{=v}_{t}\tanh\left(\frac{gt_{MD}}{v_{t}}\right)$$

$$v_{0}=0.252\;mm/s\approx 250\;mm/s$$

Therefore, the expected initial velocity resulting from acceleration due to gravity is approximately 250 mm/s.

#### 2.6.2. Expected Initial Velocities for a Downjump Based on Published Values

Previous studies have reported a range of initial take-off velocities for *Drosophila melanogaster* upjumps beginning from a stationary position on a horizontal surface. Assuming that the fly generates the same forces when jumping downward, these values can be utilized to calculate the range of expected initial velocities for a downjump at $t_{MD}$, as described above. Zumstein et al. [54] reported an initial take-off velocity of 0.61 m/s. To calculate the initial velocity, the same equation is utilized as was described above.

$$v(t)=v_{t}tanh(\;\frac{gt_{MD}}{v_{t}}+{tanh}^{-1}\left(\frac{v_{0}}{v_{t}}\right))$$

v=0.826 m/s≈ 830 mm/s

Card and Dickinson [55] reported initial velocities for two distinct jump behaviors they described as a “voluntary take-off” (0.28 m/s) and “escape take-off” (0.48 m/s). Their measurements were taken at t = 0.002 s, and thus the time term must be corrected to match the time at which measurements are calculated by the MDv2.1 software:

$$t_{MD}\;=\;\frac{3}{116}s\;-\;\frac{2}{1000}s.$$

The expected initial velocities of downjumps using the two distinct initial velocities reported by Card and Dickinson are as follows:

“Voluntary take-off”

$$v\left(t_{MD}\right)=v_{t}\tanh\left(\;\frac{gt_{MD}}{v_{t}}+{tanh}^{-1}\left(\frac{v_{0,\;\;voluntary}}{v_{t}}\right)\right)$$

v =0.503 m/s≈500 mm/s

“Escape take-off”

$$v\left(t_{MD}\right)=v_{t}\tanh\left(\;\frac{gt_{MD}}{v_{t}}+{tanh}^{-1}\left(\frac{v_{0,\;\;escape}}{v_{t}}\right)\right)$$

v=0.691 m/s≈690 mm/s

Therefore, based on the values reported by Zumstein et al. and Card and Dickinson, the initial velocity of putative downjumps is expected to range between 500 mm/s and 830 mm/s.

#### 2.6.3. Expected Initial Velocity for an Arcjump Based on Observed Height of Jump

The expected initial velocity of an arcjump can be calculated using the Newtonian equation for vertical projectile motion:

$$h_{max}=\;\frac{v_{t}}{2g}ln(1+\;\frac{v_{0}^{2}}{v_{t}^{2}})$$

where $h_{max}$ is the maximum height of the arc. Across all arcjumps, the average observed maximum height of the arc was 0.004 m. Rearranging the equation to solve for the initial velocity and substituting the necessary values provides an initial velocity of:

$$v_{0}=\;v_{t}\sqrt{e^{\left(\frac{2gh_{max}}{v_{t}^{2}}\right)}-1}\;$$

$$v_{0}=0.284\;mm/s\approx 280\;mm/s$$

Therefore, the expected initial velocity of an arcjump, based on the observed maximum height of the arc, is 280 mm/s.

#### 2.6.4. Expected Initial Velocity for an Arcjump or Upjump Based on Published Values for Upjumps

The expected initial velocity at time $t_{MD}$ can be calculated using the following equation:

$$v\left(t\right)=v_{t}\tan\left({tan}^{-1}\left(\frac{v_{0}}{v_{t}}\right)-\frac{gt}{v_{t}}\right)\;$$

Substituting the reported take-off velocity value from Zumstein et al. (0.61 m/s) provides an expected initial velocity for an arcjump or upjump of:

$$v\left(t_{MD}\right)=v_{t}\tan\left({tan}^{-1}\left(\frac{v_{0}}{v_{t}}\right)-\frac{gt_{MD}}{v_{t}}\right)$$

v=0.339 m/s ≈340 mm/s

Using the initial velocity values from Card and Dickinson for voluntary ($v_{0}=0.28\;m/s$) and escape ($v_{0}=0.48\;m/s$) take-offs and correcting for the time difference in observations provides the initial velocity values of:

“Voluntary take-off”

$$v\left(t_{MD}\right)=v_{t}\tan\left({tan}^{-1}\left(\frac{v_{0,voluntary}}{v_{t}}\right)-\frac{gt_{MD}}{v_{t}}\right)$$

v=0.044 m/s≈44 mm/s

“Escape take-off”

$$v\left(t_{MD}\right)=v_{t}\tan\left({tan}^{-1}\left(\frac{v_{0,escape}}{v_{t}}\right)-\frac{gt_{MD}}{v_{t}}\right)$$

v=0.237 m/s≈240 mm/s

Therefore, based on previous reports, upjumps and arcjumps are expected to have initial velocities between 44 mm/s and 340 mm/s.

#### 2.6.5. Estimating the Effect of Drag on Initial Velocity

To estimate the effect of drag on the initial velocity of a fall, the drag force is estimated with the standard drag equation:

$$F_{d}=\;\frac{1}{2}C_{D}A\rho v^{2}$$

where:

$$C_{D}=drag\;coefficient,$$

A=cross sectional surface  area, 

ρ=density of air, and

v=instantaneous velocity

The drag coefficient of a de-winged fly was previously estimated as $C_{D}=1$ [52]. The cross-sectional surface area of the fly was approximated as a ellipse with length (l) = 3 mm and width (w) = 2 mm. Therefore:

$$A=\;\frac{\pi }{4}lw=\;4.71\times 10^{-6}\;m^{2}$$

The density of air was assumed $\rho =1.2\;kg/m^{3}$, consistent with the air density at sea level. The instantaneous velocity can be taken as the median initial velocity across all observed falls as v=0.257 m/s. Substituting these values into the equation above provides a drag force of:

$$F_{d}=\;1.87\times 10^{-7}\;N$$

To assess the relative importance of drag, this value was compared to the gravitational force acting on the fly:

$$\frac{F_{d}}{F_{g}}=\;\frac{F_{d}}{mg}=\frac{F_{d}}{W}=0.0187\;(1.87\%)$$

The force of drag is therefore less than 2% the force of gravity at the time of the fly’s initial velocity.

### 2.7. Software Availability

All software and respective user protocols are available for free public download from the Github repository. The GitHub repository is accessible at https://github.com/johntower/MotionDetectionV2.1, URL accessed on 11 June 2026.

## 3. Results

### 3.1. The Dataset

The dataset consists of *w*[1118]-strain flies of various ages, as well as several transgene combinations in the *w*[1118] isogenic background (Tables S6–S43). The total number of flies is 232, including 174 males and 58 females. A 30 min video was generated and analyzed for each fly.

### 3.2. Video Recording and Trajectory Analysis

**Figure 1:**
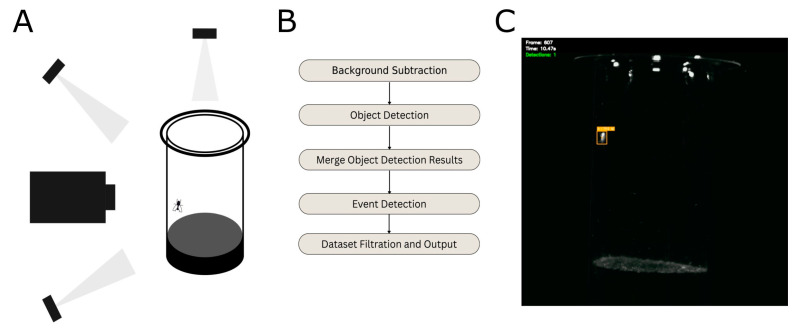
Experimental setup and MD pipeline. (**A**) Schematic of video camera, lighting, and recording chamber vial. (**B**) Flowchart diagram detailing MD object detection, event detection, and event filtration algorithms. (**C**) Representative still image of a fly in the recording chamber with the bounding box generated by the MD software. Note several spots of glare are apparent near the top of the vial.

To analyze movement, individual flies were placed in a glass culture vial covered with a glass coverslip, and illuminated with three white LED lights inside a closed box (Figure 1A). A single video camera was used to record a 30 min video at 58 fps. The resulting video is a 2D representation of the fly’s 3D movement patterns in the vial. Motion Detection (MD) V2.1 software was used to analyze the recorded video. MD identifies the fly in each frame of video as a bright object against a dark background that moves between frames (Figure 1C). MD generates a CSV file with an (x, y) position for the fly, timestamps (in seconds and frames), and bounding box dimensions (width, height, and area) for each frame in which the fly was detected.

MD analyzes the fly movement trajectories and identifies several specific events. Drops are identified by the fly moving rapidly through space from an upper position in the vial to a lower position in the vial, and are identified as movement > 2.8 mm between frame n and frame n + 2. Upward jumps (upjumps) are defined as the fly moving rapidly through space from a lower position in the vial to a higher position in the vial, with movement > 2.3 mm between frame n and frame n + 1. Arced jumps (arcjumps) are defined by the fly moving upwards from a lower position in the vial to a higher position in the vial, and then moving through space in an arced trajectory followed by a landing at a lower position in the vial. Arcjumps are identified by an upjump followed by a drop within 0.26 s.

### 3.3. Optimization and Testing of Event Scoring

The MDv2.1 software event detection thresholds were optimized using consensus events from a 30 min video that was manually annotated by two independent scorers. The manual annotation identified 32 drops, 6 upjumps and 6 arcjumps, and MDv2.1 accurately identified each event with zero false positives and zero false negatives, for an accuracy of 100% (Supplementary Table S46). The MDv2.1 software was additionally evaluated using a novel 30 min video not used during parameter optimization. Manual annotation of this video by two independent scorers identified consensus events consisting of 10 drops, 2 upjumps and 1 arcjump. MDv2.1 accurately detected 9 drops and 1 arcjump, as well as three false negatives (2 upjumps and 1 drop), and four false positive drops (Supplementary Table S47). Further inspection of the false positives by a third independent scorer indicated that all four false positives were indeed true drop events missed by the two original annotators, resulting in an accuracy of 14/17 events, or approximately 82%.

### 3.4. PCA/K-Means Analysis of Drops

Drops are expected to include at least two distinct types of events, falls and downjumps. Falls are defined as the fly losing contact with the surface of the vial and then moving downwards through space at an initial velocity determined by acceleration due to gravity. This is expected to result in an initial velocity for a fall of approximately 250 mm/s (Materials and Methods). Downjumps are defined by the fly using its legs to actively propel itself away from the surface of the vial in a downwards direction. Downjumps are expected to result in an initial velocity greater than that resulting from acceleration due to gravity.

**Figure 2:**
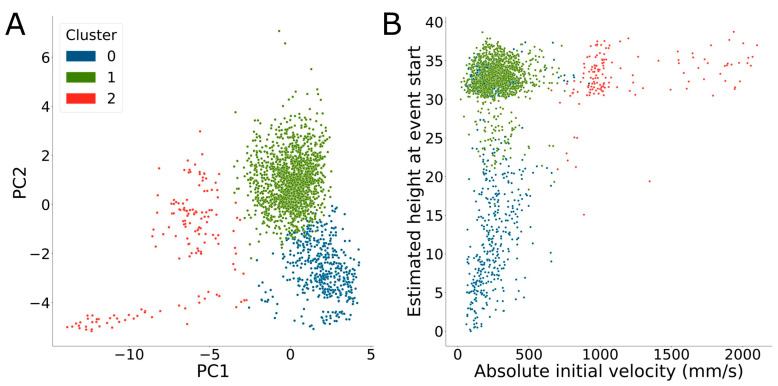
PCA/K-means analysis. (**A**) PCA projection of drop events, colored by K-means cluster assignment (k = 3). PC1 =  −0.375 (absolute initial velocity) + 0.375 (initial velocity) + 0.374 (average instantaneous velocity) + 0.371 (initial vertical velocity) + 0.366 (maximum instantaneous velocity) + 0.363 (maximum instantaneous acceleration) + 0.224 (maximum instantaneous jerk) + 0.186 (estimated height at event start) + 0.185 (vertical displacement) + 0.184 (estimated height at vertical trigger) + 0.140 (event velocity) − 0.032 (estimated height at event end) + 0.03 (elapsed time in frames) + 0.03 (elapsed time) − 0.017 (event angle) − 0.012 (estimated X position at event end) − 0.008 (horizontal displacement) − 0.004 (estimated X position at event end). PC2 = 0.469 (elapsed time in frames) + 0.469 (elapsed time in seconds) + 0.409 (vertical displacement) + 0.344 (estimated height at event start) + 0.341 (estimated height at event trigger) − 0.172 (absolute initial velocity) − 0.172 (initial velocity) − 0.159 (initial vertical velocity) − 0.158 (estimated height at event end) + 0.141 (maximum instantaneous jerk) − 0.137 (average instantaneous velocity) + 0.070 (maximum instantaneous velocity) + 0.041 (estimated X position at event start) − 0.040 (event velocity) + 0.037 (estimated X position at event end) + 0.025 (maximum instantaneous acceleration) − 0.011 (horizontal displacement) + 0.004 (event angle). (**B**) Plot of absolute initial velocity versus estimated height at event start for all drops, with events colored by the PCA/K-means cluster labels.

Analysis of the total 232 videos using MDv2.1 identified 2299 drops. This group of drops was then analyzed using Principal Component Analysis (PCA) and K-Means Clustering in an effort to distinguish between falls and downjumps. The MD software generates 25 movement metrics, including initial velocity (Table S1). Metrics that capture the change in kinematic parameters across the event (kinematic patterns; Table S1) were excluded, and therefore 18 movement metrics were utilized for the PCA/K-means. The PCA/K-means analysis was partly supervised in that an output of three clusters was specified (Figure 2A). PC1 explains approximately 31.25% of the variance in the dataset, and PC2 explains approximately 19.84% of the variance in the dataset. The PC1 equation indicates that the location of the event cluster on the PC1 axis is determined primarily by velocity metrics, where a higher PC1 value indicates lower initial velocity, lower average and maximum velocity, and reduced maximum acceleration. The PC2 equation indicates that the location of the event cluster on the PC2 axis is determined primarily by the duration of the event and the extent of downwards movement (elapsed time, height at event start, and vertical displacement). A more positive value for a drop event on the PC2 axis indicates a greater duration and greater downwards movement.

### 3.5. Initial Velocity Correlation with PCA/K-Means Identifies Falls and Downjumps

To analyze drops based on initial velocity, initial velocity was plotted versus the starting height for the event (Figure 2B). The majority of drop events are observed to begin at or near the top of the vial, with a smaller number of drops initiating from lower positions, and the majority of these events had an initial velocity of less than 500 mm/s, consistent with falls. Notably, a number of events began at or near the top of the vial, and had an initial velocity of greater than 500 mm/s, consistent with downjumps. Superimposing the PCA/K-means clusters onto the plot of initial velocity versus starting height indicates a strong correlation. Cluster 1 (green) primarily coincides with events starting near the top of the vial, and has a median initial velocity of 260 mm/s, consistent with falls. Cluster 0 (blue) primarily coincides with events starting from lower positions in the vial, and has a median initial velocity of 226 mm/s, consistent with falls. Finally, cluster 2 (red) primarily coincides with events starting near the top of the vial, and has a median initial velocity of 1005 mm/s, consistent with downjumps. The median initial velocity for the downjump cluster (cluster 2) was significantly different from that for each of the fall clusters (clusters 0 and 1) (Mann–Whitney U test, Bonferroni-adjusted *p* < 0.0001). Taken together, the PCA/K-Means Clustering and initial velocity distributions indicate that the drops can be divided into three groups, consisting of falls from near the top of the vial, falls from lower positions in the vial, and downjumps from near the top of the vial.

### 3.6. UMAP/HDBSCAN and SHAP Analysis of Drops

**Figure 3:**
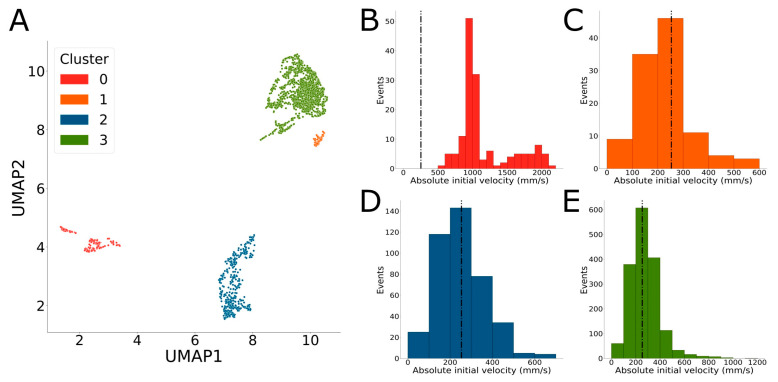
UMAP and Hierarchical Density-Based Spatial Clustering of Applications with Noise (HDBSCAN) define drops by kinematic metrics. (**A**) UMAP visualization of drop events colored by HDBSCAN-defined clusters. (**B**–**E**) Histograms of absolute initial velocities for each HDBSCAN-defined cluster. The dotted line indicates the expected initial velocity due to gravity (250 mm/s). (**B**) Cluster 0. Downjumps. (**C**) Cluster 1. Falls with missing frame data. (**D**) Cluster 2. Falls from lower in vial. (**E**) Cluster 3. Falls from top of vial.

Whereas the PCA revealed a strong linear structure in the dataset, the separation across the K-means clusters was modest (silhouette score = 0.30, Davies–Bouldin index = 1.36, Calinski–Harabasz score = 650.40), suggesting that non-linear relationships that might further differentiate distinct drop behaviors may have been missed. To address this possibility, the unsupervised Unifold Manifold Approximation and Projection (UMAP) analysis was applied to the same dataset to further explore and visualize possible nonlinear relationships between movement metrics. The UMAP visualization revealed four distinct groups of points that were well separated in space (Figure 3A). The unsupervised clustering algorithm Hierarchical Density-Based Spatial Clustering of Applications with Noise (HDBSCAN) was then applied to the UMAP-transformed data to define the clusters. The quality of the HDBSCAN-defined clusters was found to be robust (silhouette score = 0.69, Davies–Bouldin index = 0.33, Calinski–Harabasz score = 3673.14). Significant differences were observed for 14 out of the 18 movement metrics across these clusters using the Kruskal–Wallis test (Table S44). Post hoc pairwise comparisons showed that the largest differences between clusters were observed for maximum velocity, event velocity, and maximum acceleration (Table S45). Moderate to high pairwise differences were observed for initial velocity and average velocity. These findings indicate that velocity metrics primarily drive differentiation across the HDBSCAN-defined clusters.

The analysis presented above reveals which movement metrics differ on average between the HDBSCAN-defined clusters. Additional analyses were conducted to determine the relative importance of each movement metric in determining the assignment of each drop to the HDBSCAN-defined clusters. First, a Random Forest Classifier machine learning algorithm was trained using the movement metrics as predictors and the HDBSCAN-defined cluster identities as the target variable. The classifier achieved high predictive performance on novel data with a test accuracy of 0.978. Additional tests revealed a cross-validated balanced accuracy of 0.988 ± 0.016 and an out-of-bag accuracy of 0.983, indicating that the machine learning model is not over-fitting. The importance of each movement metric in determining the assignment of each drop to a cluster was assessed using Shapley Additive Explanations (SHAP) analysis, which quantifies the contributions of each movement metric to the classifier’s prediction. Cluster-wise SHAP analysis revealed which movement metrics most strongly contributed to the likelihood of drop assignment to each HDBSCAN-derived cluster, and Spearman correlations between raw feature values and the corresponding SHAP values were used to determine whether higher values increased or decreased assignment probability (Tables S2–S5).

### 3.7. Characterization of UMAP/HDBSCAN-Defined Clusters

Characterization of the UMAP/HDBSCAN-defined clusters reveals their correspondence to falls and downjumps. The strongest predictors of cluster 0 (red) membership were initial velocity, maximum acceleration, maximum velocity, and average velocity. The Spearman correlation analysis revealed that greater velocity and acceleration increased the likelihood of assignment to cluster 0 (Table S2). Cluster 0 had a median initial velocity of 1007 mm/s. This greatly exceeds the initial velocity of 250 mm/s expected from acceleration due to gravity (Figure 3B, indicated with dotted line) consistent with the conclusion that cluster 0 represents downjumps.

Cluster 2 (blue) membership was driven primarily by estimated height at event start, vertical displacement, event velocity, and initial velocity. The Spearman correlation analysis revealed that a lower start point, smaller vertical displacement, and lower velocities increased the likelihood of assignment to cluster 2 (Table S3). Cluster 2 had a median initial velocity of 241 mm/s, consistent with the velocity expected from acceleration due to gravity, and consistent with the conclusion that cluster 2 represents falls beginning from lower positions in the vial (Figure 3D).

Cluster 3 (green) membership was driven primarily by event velocity, maximum velocity, and estimated height at event start. The Spearman correlation analysis revealed that lower velocities and accelerations and a start point near the top of the vial increased the likelihood of assignment to cluster 3 (Table S4). Cluster 3 had a median initial velocity of 261 mm/s, consistent with the velocity expected from acceleration due to gravity, and consistent with the conclusion that cluster 3 represents falls from the top of the vial (Figure 3E).

Finally, cluster 1 (orange) membership was driven primarily by event velocity, maximum instantaneous velocity, and estimated height at event start. The Spearman correlation analysis revealed that lower velocities and accelerations and a start point near the top of the vial increased the likelihood of assignment to cluster 1 (Table S5). Cluster 1 had a median initial velocity of 221 mm/s consistent with the conclusion that cluster 1 also represents falls from the top of the vial (Figure 3C). However, inspection of cluster 1 events revealed that cluster 1 represented events where the fly was not detected in multiple frames between the event start and the event end. Therefore, kinematic metrics such as maximum velocity, average velocity, and maximum acceleration could not be accurately measured. Events in this cluster were confirmed to represent drops by visual inspection of the video. Consequently, cluster 1 drops (108 total) were retained for analysis of event counts but omitted from further analysis of drop kinematics. These results indicate the usefulness of UMAP for identifying data points with reduced information content.

**Figure 4:**
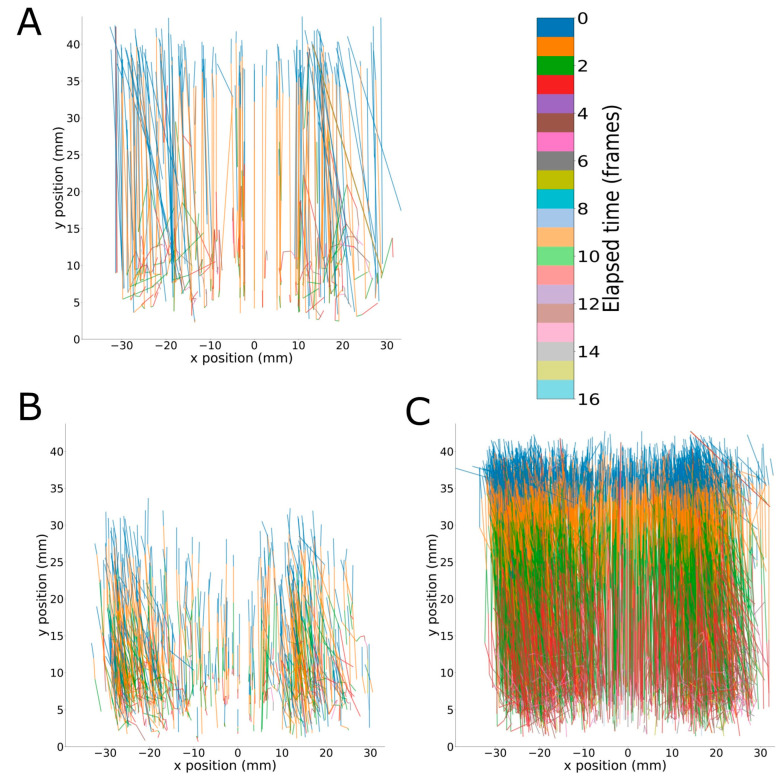
Trajectories for HDBSCAN-defined cluster events. (**A**–**C**) Color indicates the event frame progression. (**A**) Trajectories for cluster 0 downjumps from the top of the vial. (**B**) Trajectories for cluster 2 falls from lower positions in the vial. (**C**) Trajectories for cluster 3 falls from top of the vial.

Plots of drop trajectories colored by frame progression reveal a generally linear path for both downjumps (Figure 4A) and falls (Figure 4B,C). In addition, the majority of downjumps and falls are observed to end at the bottom of the vial. Downjumps typically reached the bottom of the vial within three frames, consistent with their greater velocity, whereas falls are observed to span five frames, consistent with their slower velocity.

In conclusion, the UMAP/HDBSCAN analysis results were consistent with the results of the PCA/K-means analysis, in identifying three distinct groups of events: falls from the top of the vial, falls from lower positions in the vial, and downjumps from the top of the vial. For the HDBSCAN-defined clusters, the median initial velocity for the downjump cluster (cluster 0) was significantly different from that for each of the fall clusters (clusters 2 and 3) (Mann–Whitney U test, Bonferroni-adjusted *p* < 0.0001). In addition, the median initial velocities measured for fall cluster 2 (241 mm/s; Figure 3D) and fall cluster 3 (261 mm/s; Figure 3E) are equivalent to that expected from acceleration due to gravity (250 mm/s). By contrast, the median initial velocity measured for downjump cluster 0 (1007 mm/s; Figure 3B) is significantly greater than that expected from acceleration due to gravity (Wilcoxon signed-rank test, *p* < 1 × 10^−25^).

### 3.8. Analysis of Fall and Jump Trajectories

**Figure 5:**
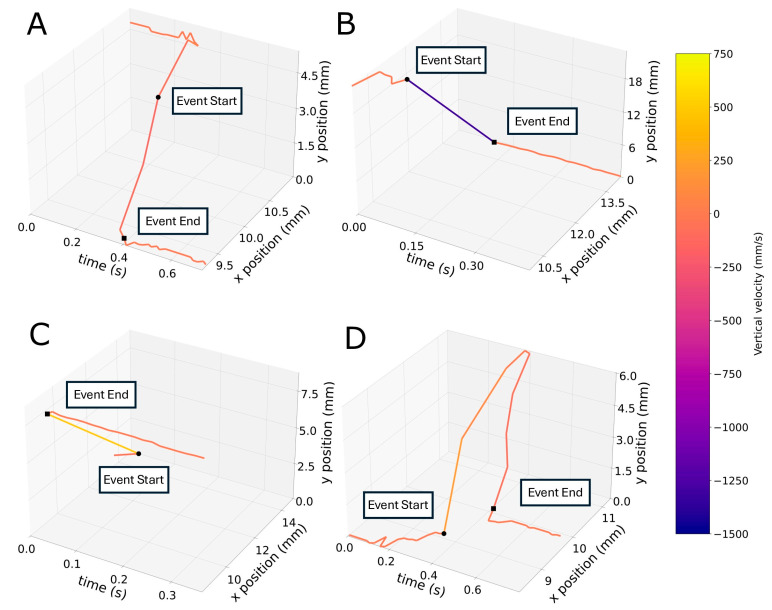
Representative trajectory for each event type. (**A**–**D**) Color signifies vertical velocity. (**A**) Representative trajectory for a fall (see Video_clip_1_fall.mp4). (**B**) Representative trajectory for a downjump (see Video_clip_2_downjump.mp4). (**C**) Representative trajectory for an upjump (see Video_clip_3_upjump.mp4). (**D**) Representative trajectory for an arcjump (see Video_clip_4_arcjump.mp4).

Event trajectories were plotted for examples of a fall, downjump, upjump and arcjump (Figure 5), and video clips for each event are included in the Supplemental Materials. The trajectories are colored by vertical velocity. The fall trajectory illustrates the moderate increase in downward velocity resulting from acceleration due to gravity (Figure 5A; clip 1). The downjump trajectory is marked by a dramatic increase in downward velocity, as expected (Figure 5B; clip 2). The upjump is marked by a rapid increase in upward velocity (Figure 5C; clip 3). Finally, the arcjump demonstrates a near-parabolic trajectory (Figure 5D; clip 4).

### 3.9. Effects of Age

To assess how aging might affect movement metrics, the drop frequency, drop movement metrics, and total movement were measured in three groups of young flies (11–13 days old, Tables S6–S8) and two groups of aged flies (40–52 days, Tables S9 and S10). The combined groups of young and old flies were then compared for potential differences in movement. Aged flies of both sexes moved significantly less than their younger counterparts, and male flies spent significantly less time in the upper half of the vial (Table 1 and Table 2), consistent with previous studies [23]. Notably, aged flies took longer to resume movement after a drop as compared to their younger counterparts (Table 1 and Table 2). While the total number of drops (the sum of falls and downjumps) was significantly reduced in aged flies, this contrast disappeared when drops were normalized to total movement, suggesting that the decrease in drops observed in aged flies may be a result of their reduced total movement (Table 1 and Table 2). For each group, accelerations calculated across the entire duration of the fall were less than expected from acceleration due to gravity, particularly for males, suggesting some mid-fall compensatory behavior to slow the fall (Table 3). This effect was reduced in the aged flies, suggesting less compensatory behavior with age. Consequently, values for initial velocity, maximum velocity, average velocity, maximum acceleration and maximum jerk were increased with age for males (Table 1 and Table 2). No statistically significant differences were observed for direct comparisons between male and female flies at either the young or old time points (Tables S11 and S12).

### 3.10. Effects of Dehydration/Starvation Stress and Genotype

Previous experiments indicated that dehydration/starvation stress resulted in an increase in drop frequency that peaked in the hours immediately preceding death [28]. Here, the effects of dehydration/starvation stress on the frequency of falls and jumps was analyzed in male flies at 24 h of treatment (Table 4). The number of total drops was doubled, but this difference did not reach statistical significance. However, the stressed flies exhibited a small but significant increase in downjumps. No significant changes in drop metrics were observed (Supplementary Table S48).

As a proof of principle, small cohorts of ND model flies and controls were also assayed. Human Abeta42-expressing female flies (AD model) were compared to age-matched controls at three different time points (7 days, 15 days, and 35 days) (Tables S15–S28). Significant differences in drop behavior were not observed in 7-day-old Abeta42 flies relative to controls (Tables S15–S20). At the 15-day time point, the Abeta42 females had significantly decreased fall frequency and total movement compared to control females (Table S21); however, these differences were not mirrored at the 35-day time point (Table S22). In summary, no consistent effect of Abeta42 expression was observed for event counts or movement metrics. In addition, human alpha-synuclein (SNCA)-expressing male flies (PD model) were compared to age-matched controls at three different time points (14 days, 21 days, and 28 days) (Tables S29–S43). SNCA males exhibited reduced total movement relative to controls at 28 days (Table S35). No significant effects were observed for event counts or specific movement metrics. Taken together, these results indicate limited effects of human Abeta42 and alpha-synuclein expression on fall frequency and drop behavior, although further investigations with larger sample sizes and extended age time points are needed.

## 4. Discussion

### 4.1. System Design

The goal of this research was to develop an economical and tractable system that would enable quantification of falls and jumps in freely moving flies. A single 58 fps video camera was used to record single flies as they moved freely in a glass culture vial. Custom software was used to track fly movement trajectories in the videos and to derive a series of movement metrics. Statistical analyses enabled identification and quantification of distinct movement behaviors, including falls, downjumps, upjumps and arcjumps. The system was designed with one camera to simplify the data analysis, as well as to favor economy and future scaling. The fly moves freely through 3D space inside the vial while the single camera captures this image as a 2D video. One limitation of this approach is that converting 3D movement patterns to a 2D pattern inherently reduces information. Vertical movement is effectively captured, whereas horizontal movement will sometimes be underestimated depending upon the orientation of the event relative to the camera. The advantages of this approach are that reducing the data to 2D patterns significantly reduces file size and processing needs and simplifies several aspects of the analysis. The present data indicate that this simplified approach is sufficient to distinguish between multiple types of complex movement behaviors, including falls, downjumps, upjumps and arcjumps.

The system presented here has certain advantages relative to the negative geotaxis assay, which is often used to quantify fly locomotor activity [16,19,26,27]. In the negative geotaxis assay, flies are knocked to the bottom of a cylinder, and then are visualized as they walk upwards away from gravity. The number of flies that reach a certain height in a defined period of time is scored. The negative geotaxis assay is generally interpreted to represent an escape response triggered by the knock-down of the fly, and the scores are driven primarily by walking speed [16]. More recent implementations of the negative geotaxis assay utilize video and also score departures from linearity in the upward climbing trajectories [27]. By contrast, when flies move freely through 3D space in a vial, they exhibit a greater variety of spontaneous exploratory and movement behaviors, including walking, turning, jumping, flying and drops. Therefore, the assay of freely moving flies captures a more complex set of movement behaviors, and potentially, a more complex set of nervous system functions. As a consequence, this assay may provide increased sensitivity and reveal additional deficits due to aging and ND models, thereby facilitating future studies of causal mechanisms and possible interventions. Existing video tracking systems could be adapted to enable assay of falls and jumps using video speeds of 58 fps or greater. In the future, it may be of interest to ask how the behavioral repertoire might change with vials of different size, or with vials containing obstacles.

### 4.2. Quantification of Jumps and Falls

Analysis of the total 232 videos identified 25 upjumps, 43 arcjumps and 2299 drops. Analysis of the drops using UMAP/HBDSCAN revealed that 2149 (93.48%) were falls and 150 (6.52%) were downjumps.

### 4.3. Comparison of Drop Event Initial Velocities to Expected Values and Previous Studies

The initial velocities measured for the different types of jumps and drops were calculated and compared to the expected initial velocities derived from kinematic calculations (Materials and Methods). As discussed above, as defined by the UMAP/HDBSCAN analysis, falls had an observed median initial velocity of 257 mm/s, consistent with the expected initial velocity resulting from acceleration due to gravity of approximately 250 mm/s. Downjumps had a median initial velocity of 1007 mm/s, which greatly exceeds the velocity expected from simple acceleration due to gravity, consistent with an initial propulsive contribution from the jump. Arcjumps exhibited a median initial velocity of 283 mm/s, which is similar to the value 280 mm/s that results from a calculation based on the maximum height of the arc (Materials and Methods).

Two previous studies have reported initial take-off velocities for upjumps, where flies were assayed as they jumped upwards from a horizontal surface [54,55]. Assuming that the fly generates the same forces when jumping downwards, these values can be utilized to calculate the range of expected initial velocities for upjumps and downjumps, and then compared to values measured in the data presented here. Zumstein et al. [54] estimated the upjump take-off velocity of wild-type flies by measuring horizontal jump distance, assuming a jump angle of 45 degrees and calculating velocity using the horizontal range formula for projectile motion. This calculation resulted in an estimated take-off velocity of approximately 610 mm/s at the moment the fly loses contact with the surface. Using this value, the expected initial velocity for an upjump in our system would be 340 mm/s, which is somewhat greater than our observed median value of 245 mm/s. The expected initial velocity of a downjump in our system using Zumstein et al.’s estimated take-off velocity is approximately 830 mm/s, which is less than our observed value of 1007 mm/s. The reasons for these differences are currently not clear but are likely to be related to differences in the biomechanics of upward jumps beginning from a horizontal surface as in the Zumstein et al. study, versus jumps beginning from a vertical surface or the ceiling of the chamber as assayed here.

Card and Dickinson [55] used video to directly assay the velocity flies jumping upwards from a horizontal surface, and reported two initial upjump velocities at t = 2 ms that they interpreted to represent two distinct jumping behaviors: voluntary take-offs (0.28 m/s) and escape take-offs (0.48 m/s). After correcting for the time interval used in their measurements and in ours, the expected initial velocities for an upjump in our system based on their results are approximately 44 mm/s for a voluntary take-off and approximately 240 mm/s for an escape take-off. The latter is consistent with our observed median of 245 mm/s. Therefore, the initial velocities measured here for upjumps were in general agreement with the values reported from previous studies of upjumps.

Again using the upjump velocities reported by Card and Dickenson [55], and applying a temporal correction for downjumps, where acceleration due to gravity additionally increases velocity, the expected initial velocities in our system are approximately 500 mm/s for voluntary take-off, and approximately 690 mm/s for an escape take-off. These values are significantly lower than our observed initial velocities for a downjump, which had a median of 1007 mm/s. The reason for this difference is currently unresolved, but is likely to be related to biomechanical differences between initiating upjumps versus downjumps.

### 4.4. Effects of Fly Age

Several movement metrics were observed to be affected by fly age in *w*[1118]-strain flies. For example, aged flies of both sexes moved less and spent less time in the upper half of the vial, consistent with previous studies [16,21,22]. Notably, mid-event fall accelerations were reduced relative to that expected from acceleration due to gravity, preferentially in younger animals, indicating some compensatory behavior that is reduced with age. It is possible that young animals slow the descent of their fall using their wings, or by touching the wall of the vial with their legs, and older animals are less effective in this response. The estimated force of drag is negligible (less than 2%) relative to the force of gravity during a fall for a wingless fly (Materials and Methods). However, any wing extension would be expected to significantly increase drag and slow the fall. The current system does not enable analysis of the orientation of the fly or its potential use of wings or legs during the fall; however, this may be possible in the future using faster video speeds. This would also facilitate analysis of the possible causes for falls, such as slips, tremors or other movements. In the future, it will be of interest to further explore the effects of age on the biomechanics of falls and jumps.

Aged flies were also observed to take approximately 5 times (males) or 9 times (females) longer than young flies to resume movement after falling to the bottom of the vial, a period referred to here as the recovery time. This increased recovery time might be related to the fact that total movement activity in the aged flies was reduced by approximately 6 times (males) and 3 times (females); however, the fold increase in recovery time is greater than the fold reduction in total movement, at least for females. Another possibility is that the impact from the fall causes some disturbance in the fly that delays the resumption of movement. This is reminiscent of the “bang-senseless” phenotype, which is a temporary paralysis that results when the investigator intentionally knocks a mutant fly to the bottom of a vial. The bang-senseless phenotype is observed in flies with mutations that affect mitochondrial and nervous system functions, and worsens with age [56]. Quantification of the bang-senseless phenotype in aging wild-type flies has generally not been reported; however, our anecdotal observations suggest that flies at late ages express a phenotype resembling bang-senseless. Therefore, one possible interpretation of the increased recovery time observed here for the aged flies is that this represents a brief period of uncoordinated movement or paralysis caused by the impact. Impacts of falling and jumping flies with the sides of the vial may also be relevant. To what extent the repeated impacts of falling and jumping flies with the bottom and sides of the vial might contribute to anatomical damage remains unclear. Notably, flies that are near death exhibit increased supine behavior (fly on its back), and a significant fraction of flies are found dead in the supine position [23,24,25]. In the future, it may be of interest to investigate how falls and the resulting impact might contribute to supine behavior and mortality. In addition, recovery time might prove to be a useful metric in future assays of aging, ND models and interventions.

### 4.5. Effects of Stress and Genotype

Dehydration/starvation stress was associated with a small but significant increase in the number of downjumps, which might indicate increased foraging activity or a possible maladaptive hyperactivity. Neither AD nor PD model flies showed significant differences in the frequency of falls or jumps, which may result from the small sample sizes and limited age ranges tested.

### 4.6. Future Directions

Future directions will include further analysis of landings, recovery time and supine behavior. The biomechanics of fall initiations and compensatory behaviors, and how these change with age will be of particular interest. The *Drosophila* strain used here is the common *w*[1118] reference strain, which lacks eye pigments and cannot sense visual light. In the future, it will be of interest to compare wild-type and mutant strains to assay the potential effects of vision on falls and jumps and other behaviors. The methods developed here should facilitate further analysis of the effects of aging and ND models on locomotor behaviors and falls, including the testing of potential drug and environmental interventions.

## Figures and Tables

**Table 1:** Comparing event counts and drop metrics of 11–13-day-old *w*[1118] males (n = 18) to 40–52-day-old *w*[1118] males (n = 16). Pooled data from Supplementary Tables S6–S10. *p*-value for significance is *p* < 0.05. Metric sub-types are indicated in bold font.

	Young *w*[1118] Male			Old *w*[1118] Male				
Metric	Mean	SD	Median	Mean	SD	Median	*p* Value	Test
**Event Counts**								
Upward Jumps	0.000	0.000	0.000	0.000	0.000	0.000	-	Welch’s *t*-test
Arcjumps	0.222	0.711	0.000	0.062	0.242	0.000	0.623	Mann–Whitney U test
Falls	9.111	9.410	7.000	1.938	3.579	1.000	2.42 × 10^−4^	Mann–Whitney U test
Downward Jumps	0.167	0.373	0.000	0.312	0.682	0.000	0.775	Mann–Whitney U test
Drops	9.278	9.409	7.500	2.250	4.054	1.000	3.39 × 10^−4^	Mann–Whitney U test
Total Events	9.500	9.523	7.500	2.312	4.027	1.000	2.48 × 10^−4^	Mann–Whitney U test
Total Movement (mm)	8.95 × 10^3^	2.93 × 10^3^	9.50 × 10^3^	1.72 × 10^3^	770.595	1.95 × 10^3^	1.09 × 10^−5^	Mann–Whitney U test
Time spent in upper half of vial	0.607	0.179	0.637	0.423	0.179	0.426	1.26 × 10^−3^	Mann–Whitney U test
Event–Movement ratio	9.77 × 10^−4^	8.43 × 10^−4^	8.06 × 10^−4^	1.05 × 10^−3^	1.29 × 10^−3^	8.60 × 10^−4^	0.665	Mann–Whitney U test
**Drop Metrics**								
Event duration (frames)	4.588	0.878	4.682	4.692	1.617	4.500	0.820	Student’s *t*-test
Event duration (s)	0.079	0.015	0.081	0.081	0.028	0.078	0.820	Student’s *t*-test
Vertical displacement (mm)	23.419	4.938	24.375	22.540	9.520	24.279	0.751	Welch’s *t*-test
Horizontal displacement (mm)	3.658	1.536	3.838	4.460	2.405	4.146	0.264	Student’s *t*-test
Estimated height at event trigger (mm)	14.498	6.412	12.802	15.060	9.989	10.225	0.501	Mann–Whitney U test
Estimated height at event start (mm)	13.577	5.555	11.765	13.829	8.387	10.225	0.546	Mann–Whitney U test
Estimated X position at event start (mm)	22.139	2.027	22.694	21.912	3.799	21.326	0.832	Student’s *t*-test
Estimated height at event end (mm)	36.996	2.462	36.809	36.369	8.163	38.726	0.162	Mann–Whitney U test
Estimated X position at event end (mm)	21.577	1.937	21.860	20.827	3.775	21.091	0.495	Welch’s *t*-test
Event velocity (mm/s)	495.790	154.051	502.506	401.789	182.902	361.466	0.125	Student’s *t*-test
Maximum velocity (mm/s)	447.444	103.866	459.661	869.971	383.272	767.713	6.93 × 10^−4^	Welch’s *t*-test
Average velocity (mm/s)	266.253	46.940	270.112	410.761	185.553	393.163	9.39 × 10^−3^	Welch’s *t*-test
Initial vertical velocity (mm/s)	240.869	59.731	219.909	335.335	206.500	243.195	0.523	Mann–Whitney U test
Maximum acceleration (mm/s^2^)	1.88 × 10^4^	4.52 × 10^3^	1.77 × 10^4^	4.44 × 10^4^	2.24 × 10^4^	4.11 × 10^4^	4.93 × 10^−4^	Welch’s *t*-test
Initial velocity (mm/s)	255.023	55.302	243.071	389.014	208.786	342.968	0.028	Welch’s *t*-test
Absolute initial velocity (mm/s)	255.023	55.302	243.071	389.014	208.786	342.968	0.028	Welch’s *t*-test
Maximum jerk (mm/s^3^)	1.05 × 10^6^	6.60 × 10^5^	1.06 × 10^6^	3.38 × 10^6^	2.59 × 10^6^	2.57 × 10^6^	3.58 × 10^−3^	Welch’s *t*-test
Lag time (s)	2.122	1.483	1.662	10.132	10.354	5.505	0.011	Mann–Whitney U test
Event Angle (degrees)	88.895	9.952	90.643	91.553	11.086	94.97	0.429	Mann–Whitney U test

**Table 2:** Comparing event counts and drop metrics of 11–13-day-old *w*[1118] females (n = 18) to 40–52-day-old *w*[1118] females (n = 16). Pooled data from Supplementary Tables S6–S10. *p*-value for significance is *p* < 0.05. Metric sub-types are indicated in bold font.

	Young *w*[1118] Female			Old *w*[1118] Female				
Metric	Mean	SD	Median	Mean	SD	Median	*p* Value	Test
**Event Counts**								
Upward Jumps	0.111	0.314	0.000	0.125	0.331	0.000	0.926	Mann–Whitney U test
Arcjumps	0.111	0.314	0.000	0.062	0.242	0.000	0.648	Mann–Whitney U test
Falls	6.111	4.736	5.000	2.812	2.789	2.000	0.011	Mann–Whitney U test
Downward Jumps	0.333	0.577	0.000	0.438	0.704	0.000	0.763	Mann–Whitney U test
Drops	6.444	5.002	5.000	3.250	3.211	2.000	0.022	Mann–Whitney U test
Total Events	6.667	4.978	5.500	3.438	3.278	2.000	0.020	Mann–Whitney U test
Total Movement (mm)	7.19 × 10^3^	3.50 × 10^3^	6.91 × 10^3^	2.65 × 10^3^	2.01 × 10^3^	2.62 × 10^3^	9.35 × 10^−5^	Welch’s *t*-test
Time spent in upper half of vial	0.652	0.089	0.683	0.475	0.280	0.545	0.070	Mann–Whitney U test
Event–Movement ratio	9.84 × 10^−4^	4.76 × 10^−4^	9.15 × 10^−4^	1.22 × 10^−3^	1.06 × 10^−3^	8.79 × 10^−4^	1.000	Mann–Whitney U test
**Drop Metrics**								
Event duration (frames)	4.597	1.509	4.667	4.911	1.634	4.222	0.783	Mann–Whitney U test
Event duration (s)	0.079	0.026	0.080	0.085	0.028	0.073	0.820	Mann–Whitney U test
Vertical displacement (mm)	25.817	5.150	24.857	26.248	5.338	30.004	0.878	Student’s *t*-test
Horizontal displacement (mm)	3.411	1.228	3.323	3.641	2.357	2.429	0.784	Student’s *t*-test
Estimated height at event trigger (mm)	13.259	6.690	10.604	12.199	4.118	11.547	0.880	Mann–Whitney U test
Estimated height at event start (mm)	12.352	5.799	9.986	11.500	3.849	11.136	0.820	Mann–Whitney U test
Estimated X position at event start (mm)	21.909	4.679	21.344	22.850	3.651	20.762	0.697	Student’s *t*-test
Estimated height at event end (mm)	38.169	3.580	38.142	37.748	3.099	37.739	0.823	Student’s *t*-test
Estimated X position at event end (mm)	21.718	4.061	21.833	21.436	5.197	22.153	0.904	Student’s *t*-test
Event velocity (mm/s)	567.627	366.109	469.709	332.595	67.137	347.093	0.039	Mann–Whitney U test
Maximum velocity (mm/s)	621.872	395.772	493.323	841.449	225.654	791.430	0.058	Mann–Whitney U test
Average velocity (mm/s)	398.451	388.433	279.501	360.295	54.367	375.237	0.319	Mann–Whitney U test
Initial vertical velocity (mm/s)	386.151	382.422	306.397	292.713	110.141	312.638	0.940	Mann–Whitney U test
Maximum acceleration (mm/s^2^)	2.76 × 10^4^	2.27 × 10^4^	2.08 × 10^4^	3.68 × 10^4^	1.66 × 10^4^	3.48 × 10^4^	0.189	Mann–Whitney U test
Initial velocity (mm/s)	396.832	389.518	307.511	305.631	102.199	325.670	0.940	Mann–Whitney U test
Absolute initial velocity (mm/s)	396.832	389.518	307.511	305.631	102.199	325.670	0.940	Mann–Whitney U test
Maximum jerk (mm/s^3^)	1.17 × 10^6^	8.59 × 10^5^	9.55 × 10^5^	3.45 × 10^6^	1.92 × 10^6^	2.99 × 10^6^	0.075	Welch’s *t*-test
Lag time (s)	3.326	4.022	2.146	31.172	47.877	6.120	0.011	Mann–Whitney U test
Event Angle (degrees)	90.497	5.985	89.763	91.902	7.083	92.057	0.678	Student’s *t*-test

**Table 3:** Comparison of fall accelerations and fall initial velocities of young and aged *w*[1118] flies to those expected from acceleration due to gravity. Fall accelerations were calculated using linear regression of frame-wise velocity values across the fall. The fall acceleration values are compared to that expected from acceleration due to gravity (9810 mm/s^2^) using a Wilcoxon signed-rank test. Fall initial velocities for each group were compared to that expected from acceleration due to gravity (250 mm/s) using a Wilcoxon signed-rank test. The young groups were 11–13 days old, and the old groups were 40–52 days old. *p*-value for significance is *p* < 0.05. Kinematic parameters are indicated in bold font.

	Young *w*[1118] Males	Young *w*[1118]Females	Old *w*[1118] Males	Old *w*[1118] Females
n*_flies_*	18	18	16	16
n*_falls_*	104	69	26	38
**Acceleration (mm/s^2^)**				
Expected	9810	9810	9810	9810
Median	1843.279	2607.147	5314.073	3488.99
W statistic	321	295	69	77
Z-score	−7.812	−5.546	−2.705	−4.256
*p* value	2.82 × 10^−15^	2.43 × 10^−9^	0.003	2.07 × 10^−6^
**Initial velocity (mm/s)**				
Expected	250	250	250	250
Median	211.838	227.233	240.798	244.346
W statistic	1411.0	1062.0	151.0	344.5
Z-score	−4.277	−0.87	−0.622	−0.377
*p* value	1.89 × 10^−5^	0.384	0.548	0.706

**Table 4:** Comparing event counts of 2-day-old *w*[1118] males (n = 6) to 2-day-old dehydrated *w*[1118] males (n = 7). Pooled data from Supplementary Tables S13 and S14. *p*-value for significance is *p* < 0.05. Drop metrics presented in Supplementary Table S48. Metric sub-type is indicated in bold font.

	*w*[1118] Male			Dehydrated *w*[1118] Male				
Metric	Mean	SD	Median	Mean	SD	Median	*p* Value	Test
**Event Counts**								
Upward Jumps	0.000	0.000	0.000	0.429	0.495	0.000	0.097	Mann–Whitney U test
Arcjumps	0.000	0.000	0.000	0.143	0.350	0.000	0.440	Mann–Whitney U test
Falls	7.333	7.386	5.500	17.857	24.439	10.000	0.517	Mann–Whitney U test
Downward Jumps	0.167	0.373	0.000	1.571	1.050	2.000	0.033	Mann–Whitney U test
Drops	7.500	7.228	5.500	19.429	24.761	12.000	0.431	Mann–Whitney U test
Total Events	7.500	7.228	5.500	20.000	24.646	13.000	0.391	Mann–Whitney U test
Total Movement (mm)	7.61 × 10^3^	5.08 × 10^3^	8.14 × 10^3^	7.43 × 10^3^	3.98 × 10^3^	7.52 × 10^3^	0.949	Student’s *t*-test
Time spent in upper half of vial	0.579	0.066	0.557	0.526	0.189	0.609	0.565	Student’s *t*-test
Event–Movement ratio	7.52 × 10^−4^	5.77 × 10^−4^	7.39 × 10^−4^	1.97 × 10^−3^	1.94 × 10^−3^	1.81 × 10^−3^	0.200	Student’s *t*-test

## Data Availability

The raw data supporting the conclusions of this article will be made available by the authors on request.

## Supplementary Materials

The following supporting information can be downloaded at: https://www.mdpi.com/article/10.3390/insects17060624/s1, Supplementary Materials: Supplementary Table S1. Definitions of derived kinematic metrics provided for each event. Supplementary Table S2. Mean absolute SHAP values and Spearman correlation of features for Cluster 0, as defined by HDBSCAN clustering on UMAP-transformed drop data. Supplementary Table S3. Mean absolute SHAP values and Spearman correlation of features for Cluster 2, as defined by HDBSCAN clustering on UMAP-transformed drop data. Supplementary Table S4. Mean absolute SHAP values and Spearman correlation of features for Cluster 3, as defined by HDBSCAN clustering on UMAP-transformed drop data. Supplementary Table S5. Mean absolute SHAP values and Spearman correlation of features for Cluster 1, as defined by HDBSCAN clustering on UMAP-transformed drop data. Supplementary Table S6. Comparing event counts and drop metrics of 12-day-old *w*[1118] males (n = 6) to 12-day-old *w*[1118] females (n = 6). Supplementary Table S7. Comparing event counts and drop metrics of 11-day-old *w*[1118] males (n = 6) to 11-day-old *w*[1118] females (n = 6). Supplementary Table S8. Comparing event counts and drop metrics of 13-day-old *w*[1118] males (n = 6) to 13-day-old *w*[1118] females (n = 6). Supplementary Table S9. Comparing event counts and drop metrics of 52-day-old *w*[1118] males (n = 8) to 52-day-old *w*[1118] females (n = 8). Supplementary Table S10. Comparing event counts and drop metrics of 40-day-old *w*[1118] males (n = 8) to 40-day-old *w*[1118] females (n = 8). Supplementary Table S11. Comparing event counts and drop metrics of 11-13 day old *w*[1118] males (n = 18) to 11-13 day old *w*[1118] females (n = 18). Supplementary Table S12. Comparing event counts and drop metrics of 40-52 day old *w*[1118] males (n = 16) to 40-52 day old *w*[1118] females (n = 16). Supplementary Table S13. Comparing event counts and drop metrics of 2-day-old *w*[1118] males (n = 3) to 2-day-old dehydrated *w*[1118] males (n = 4), Cohort 1. Supplementary Table S14. Comparing event counts and drop metrics of 2-day-old *w*[1118] males (n = 3) to 2-day-old dehydrated *w*[1118] males (n = 3), Cohort 2. Supplementary Table S15. Comparing event counts and drop metrics of 7-day-old control females (n = 4) to 7-day-old Abeta females (n = 4). Supplementary Table S16. Comparing event counts and drop metrics of 7-day-old control males (n = 8) to 7-day-old Abeta males (n = 7), Cohort 1. Supplementary Table S17. Comparing event counts and drop metrics of 7-day-old control males (n = 4) to 7-day-old Abeta males (n = 4), Cohort 2. Supplementary Table S18. Comparing event counts and drop metrics of 7-day-old control males (n = 12) to 7-day-old Abeta males (n = 11). Supplementary Table S19. Comparing event counts and drop metrics of 7-day-old Abeta42 males (n = 12) to 7-day-old Abeta females (n = 4). Supplementary Table S20. Comparing event counts of 7-day-old *w*[1118] males (n = 12) to 7-day-old *w*[1118] females (n = 4). Supplementary Table S21. Comparing event counts and drop metrics of 15-day-old control females (n = 4) to 15-day-old Abeta females (n = 4). Supplementary Table S22. Comparing event counts and drop metrics of 35-day-old control females (n = 4) to 35-day-old Abeta females (n = 4). Supplementary Table S23. Comparing event counts and drop metrics of 7-day-old Abeta females (n = 4) to 15-day-old Abeta females (n = 4). Supplementary Table S24. Comparing event counts and drop metrics of 7-day-old *w*[1118] females (n = 4) to 15-day-old *w*[1118] females (n = 4). Supplementary Table S25. Comparing event counts and drop metrics of 15-day-old Abeta females (n = 4) to 35-day-old Abeta females (n = 4). Supplementary Table S26. Comparing event counts and drop metrics of 15-day-old *w*[1118] females (n = 4) to 35-day-old *w*[1118] females (n = 4). Supplementary Table S27. Comparing event counts and drop metrics of 15-day-old Abeta females (n = 4) to 35-day-old Abeta females (n = 4). Supplementary Table S28. Comparing event counts and drop metrics of 7-day-old *w*[1118] females (n = 4) to 35-day-old *w*[1118] females (n = 4). Supplementary Table S29. Comparing event counts and drop metrics of 2-week-old control males (n = 8) to 2-week-old SNCA males (n = 8), Cohort 1. Supplementary Table S30. Comparing event counts and drop metrics of 2-week-old control males (n = 8) to 2-week-old SNCA males (n = 8), Cohort 2. Supplementary Table S31. Comparing event counts and drop metrics of 2-week-old control males (n = 8) to 2-week-old SNCA males (n = 8), Cohort 3. Supplementary Table S32. Comparing event counts and drop metrics of 3-week-old control males (n = 4) to 3-week-old SNCA males (n = 8), Cohort 1. Supplementary Table S33. Comparing event counts and drop metrics of 3-week-old control males (n = 8) to 3-week-old SNCA males (n = 8), Cohort 2. Supplementary Table S34. Comparing event counts and drop metrics of 3-week-old control males (n = 8) to 3-week-old SNCA males (n = 8), Cohort 3. Supplementary Table S35. Comparing event counts and drop metrics of 4-week-old control males (n = 6) to 4-week-old SNCA males (n = 6). Supplementary Table S36. Comparing event counts and drop metrics of 2-week-old control males (n = 24) to 2-week-old SNCA males (n = 24). Supplementary Table S37. Comparing event counts and drop metrics of 3-week-old control males (n = 20) to 3-week-old SNCA males (n = 24). Supplementary Table S38. Comparing event counts and drop metrics of 2-week-old SNCA males (n = 24) to 3-week-old SNCA males (n = 24). Supplementary Table S39. Comparing event counts and drop metrics of 2-week-old *w*[1118] males (n = 24) to 3-week-old *w*[1118] males (n = 20). Supplementary Table S40. Comparing event counts and drop metrics of 3-week-old SNCA males (n = 24) to 4-week-old SNCA males (n = 6). Supplementary Tables S41. Comparing event counts and drop metrics of 3-week-old *w*[1118] males (n = 20) to 4-week-old *w*[1118] males (n = 6). Supplementary Table S42. Comparing event counts and drop metrics of 2-week-old SNCA males (n = 24) to 4-week-old SNCA males (n = 6). Supplementary Table S43. Comparing event counts and drop metrics of 2-week-old *w*[1118] males (n = 24) to 4-week-old *w*[1118] males (n = 6). Supplementary Table S44. Kruskal-Wallis test comparing drop metrics between HDBSCAN-defined clusters. Supplementary Table S45. Post-Hoc Dunn’s Test comparing drop metrics between HDBSCAN-derived clusters. Supplementary Table S46. Comparison of manually-annotated events and events detected by MDv2.1 on reference video. Supplementary Table S47. Comparison of manually-annotated events and events detected by MDv2.1 on novel data. Supplementary Table S48. Comparing drop metrics of 2 day old *w*[1118] males (n = 6) to 2 day old dehydrated *w*[1118] males (n = 7). Video clips 1–4.

## Abbreviations

The following abbreviations are used in this manuscript:

PCA	Principal Component Analysis
UMAP	Uniform Manifold Approximation and Projection
HDBSCAN	Hierarchical Density-Based Spatial Clustering with Applications to Noise
SHAP	Shapley Additive Explanations
MD	Motion Detection
ND	Neurodegenerative disease
AD	Alzheimer’s disease
PD	Parkinson’s disease
