# Supplementary material for: Analysis of Fall and Jump Behaviors in Freely Moving Drosophila melanogaster Using 58 fps Video
Source: Insects. 2026 Jun 13;17(6):624. doi: 10.3390/insects17060624 (PMC13299798; doi:10.3390/insects17060624)
Supplement: Supplementary file 1 [file insects-17-00624-s001.zip › Das et al Supplementary Materials Revised.pdf]

Analysis of fall and jump behaviors in freely-moving *Drosophila melanogaster* using 58fps video

Shoham Das , Yash Patel , Kyle Wang , John Tower

Supplementary Materials
Supplemental Tables S1-S48

Supplementary Table S1. Definitions of derived kinematic metrics provided for each event.

Metric	*Definition*	*Calculation*	*Units*
Temporal bounds of an event			
Time at event trigger	The time at which the fly's vertical displacement first exceeds an event displacement threshold.<br><br>For downward events, the fly must move a minimum of 60 pixels downwards from $frame_{n}$ to $frame_{n+2}$. These first three frames are numbered as 0, 1, 2. For upward events, the fly must move a minimum of 50 pixels upward from $frame_{n}$ to $frame_{n+1}$. These first three frames are numbered as 0 and 1.	$t_{event\ trigger}$	s
Time at event start	Time at event trigger is adjusted back to $frame_{n-1}$ if $frame_{n-1}$ is consecutive and the vertical displacement from $frame_{n}$ to $frame_{n-1}$ is greater than 20 pixels.	$t_{event\ start} = t_{event\ trigger} - 1$	s
Time at event end	The time at which either three consecutive frames show less than 5 pixels of vertical movement, or the cumulative vertical displacement across three frames is less than 10 pixels.	$t_{event\ end}$	s
Estimated height at event trigger	The vertical position of the fly at event trigger	$y_{event\ trigger} = y\ at\ t_{event\ trigger}$	mm
Estimated height at event start	The vertical position of the fly at event start	$y_{event\ start} = y\ at\ t_{event\ start}$	mm
Estimated X position at event start	The horizontal position of the fly at event start	$x_{event\ start} = x\ at\ t_{event\ start}$	mm
Estimated height at event end	The vertical position of the fly at the end of the event	$y_{event\ end} = y\ at\ t_{event\ end}$	mm
Estimated X position at event end	The horizontal position of the fly at the end of the event	$x_{event\ end} = x_{event\ end}$	mm
Event-level kinematic measures			
Event duration	The length of time between the start and end of the event	$\Delta t = t_{event\ end} - t_{event\ start}$	frames or seconds
Vertical displacement	The vertical displacement of the fly between the start and end of the event	$\Delta y = y_{event\ end} - y_{event\ start}$	mm
Horizontal displacement	The horizontal displacement of the fly between the start and end of the event	$\Delta x = x_{event\ end} - x_{event\ start}$	mm
Event velocity	The velocity calculated between event start and event end	$v_x = \frac{\Delta x}{\Delta t}$<br>$v_y = \frac{\Delta y}{\Delta t}$<br>$v = \sqrt{v_y^2 + v_x^2}$	mm/s
Event angle	The angle calculated across the entire event.	$\theta = \arctan\left(\frac{\Delta y}{\Delta x}\right)$	degrees
Kinematic patterns			
Vertical displacement pattern	Vertical movement for each frame across the event. If a detection is missing in a frame, the data for that frame is not utilized.	$\Delta y_t = y_t - y_{t-1}, t = 1,2,\ldots,n$<br>The vertical movement pattern ($y_{pattern}$) contains all of the instantaneous vertical movements across an event, as calculated using the equation above.	mm
Horizontal displacement pattern	Horizontal movement for each frame across the event. If a detection is missing in a frame, the data for that frame is not utilized.	$\Delta x_t = x_t - x_{t-1}, t = 1,2,\ldots,n$<br>The horizontal movement pattern ($x_{pattern}$) contains all of the instantaneous horizontal movements across an event, as calculated using the equation above.	mm
Vertical velocity pattern	Vertical velocity at each frame across the event. If a detection is missing in a frame, the data for that frame is not utilized.	$v_{y,t} = \frac{\Delta y_t}{\Delta t}, t = 1,2,\ldots,n$	mm/s

		The vertical velocity pattern ($v_{ypattern}$) contains all of the vertical velocities across an event, as calculated using the equation above.	
Velocity pattern	Velocity at each frame across the event. If a detection is missing in a frame, the data for that frame is not utilized.	$v_{x,t} = \frac{\Delta x_t}{\Delta t}, t = 1,2, \dots n$<br><br>$v_t = \sqrt{v_{y,t}^2 + v_{x,t}^2}, t = 1,2, \dots n$<br>The velocity pattern ($v_{pattern}$) contains all of the velocities across an event, as calculated using the equation above.	mm/s
Acceleration pattern	Acceleration at each frame across the event. If a detection is missing in a frame, the data for that frame is not utilized.	$a_t = \frac{v_t - v_{t-1}}{\Delta t}, t = 1,2, \dots, n$<br><br>The acceleration pattern ($a_{pattern}$) contains all of the accelerations across an event, as calculated using the equation above.	mm/s^2
Jerk pattern	Jerk at each frame across the event. If a detection is missing in a frame, the data for that frame is not utilized. Jerk is defined by the change in acceleration with respect to time. This metric is calculated internally but not included in the final CSV files.	$j_t = \frac{a_t - a_{t-1}}{\Delta t}, t = 1,2, \dots, n$<br><br>The jerk pattern ($j_{pattern}$) contains all of the accelerations across an event, as calculated using the equation above.	mm/s^3
Angle pattern	Movement angle at each frame across the event. If a detection is missing in a frame, the data for that frame is not utilized.	$\theta = tan^{-1}(\frac{\Delta y_t}{\Delta x_t})$<br>The angle pattern ($angle_{pattern}$) contains all of the movement angles across an event, as calculated using the equation above. The horizontal is considered 0° and the downward vertical is considered 90°	°
Frame-wise kinematic metrics			
Maximum velocity	The maximum velocity observed during the event.	$v_{max} = max\ (v_{pattern})$	mm/s
Average velocity	The average frame-wise velocity observed across the event.	$\bar{v}_{inst} = \frac{1}{n}\sum_{t=0}^{n} v_{pattern}$	mm/s
Initial vertical velocity	The median of the vertical velocities calculated at frame 1 and at frame 2. across the first three frames, from the event start (frame 0) to frame 2. If a fly is not detected in frame 2, initial vertical velocity is calculated from event start (frame 0) to frame 1.	$v_{y_0} = median(v_{y,t_1}, v_{y,t_2})$<br><br>$if\ v_{y,t_2}\ unavailable, use$<br>$v_{y_0} = v_{y,t_1}$	mm/s
Initial velocity	The median velocity across the first three frames, from the event start (frame 0) to frame 2. If a fly is not detected in frame 2, initial vertical velocity is calculated from event start (frame 0) to frame 1.	$v_0 = median(v_{t_1}, v_{t_2})$<br><br>$if\ v_{t_2}\ unavailable, use$<br>$v_0 = v_{t_1}$	mm/s
Absolute initial velocity	The absolute value of initial velocity	$v_{0,abs} = \lvert v_0 \rvert$	mm/s
Maximum acceleration	The maximum acceleration observed during the event.	$a_{max} = max\ (a_{pattern})$	mm/s^2
Maximum jerk	The maximum jerk observed during the event.	$j_{max} = max\ (j_{pattern})$	mm/s^3
Lag time	The number of seconds after the end of the event required for the fly to move two body lengths.	$lag\ time = k - t_{event\ end}$<br><br>$where\ k\ satisfies$<br><br>$i = \sum_{t_{event\ end}+1}^{k} \sqrt{(x_i - x_{i-1})^2 + (y_i - y_{i-1})^2} \geq 2L$<br><br>*where L is the approximate body length of a Drosophila melanogaster fly (3.0mm)*	s

Supplementary Table S2. Mean absolute SHAP values and Spearman correlation of features for Cluster 0, as defined by HDBSCAN clustering on UMAP-transformed drop data.

Feature	*Mean Absolute SHAP Value*	*Spearman Correlation*
Initial velocity (mm/s)	0.076	0.033
Absolute initial velocity (mm/s)	0.067	0.009
Initial vertical velocity (mm/s)	0.057	0.409
Maximum acceleration (mm/s^2)	0.042	0.371
Maximum velocity (mm/s)	0.036	0.539
Average velocity (mm/s)	0.007	0.792
Estimated height at event start (mm)	0.005	0.506
Estimated height at event trigger (mm)	0.005	0.427
Maximum jerk (mm/s^3)	0.004	0.581
Vertical displacement (mm)	0.004	0.381
Event velocity (mm/s)	0.002	0.300
Event duration (frames)	0.002	-0.639
Event duration (s)	0.001	-0.637
Estimated height at event end (mm)	0.001	0.347
Estimated X position at event end (mm)	0.000	0.247
Estimated X position at event start (mm)	0.000	0.186
Horizontal displacement (mm)	0.000	-0.229
Event angle (degrees)	0.000	-0.620

Supplementary Table S3. Mean absolute SHAP values and Spearman correlation of features for Cluster 2, as defined by HDBSCAN clustering on UMAP-transformed drop data.

Feature	*Mean Absolute SHAP Value*	*Spearman Correlation*
Estimated height at event start (mm)	0.140	-0.463
Estimated height at event trigger (mm)	0.126	-0.453
Vertical displacement (mm)	0.041	-0.662
Event Velocity (mm/s)	0.020	-0.758
Initial velocity (mm/s)	0.015	-0.079
Absolute initial velocity (mm/s)	0.014	-0.050
Maximum acceleration (mm/s^2)	0.011	-0.637
Maximum velocity (mm/s)	0.010	-0.860
Initial vertical velocity (mm/s)	0.008	-0.155
Event duration (s)	0.008	-0.868
Estimated height at event end (mm)	0.008	-0.783
Event duration (frames)	0.007	-0.838
Maximum jerk (mm/s^3)	0.006	-0.088
Average velocity (mm/s)	0.002	-0.902
Horizontal displacement (mm)	0.001	0.664
Estimated X position at event end (mm)	0.001	0.851
Event angle (degrees)	0.001	0.698
Estimated X position at event start (mm)	0.000	-0.562

Supplementary Table S4. Mean absolute SHAP values and Spearman correlation of features for Cluster 3, as defined by HDBSCAN clustering on UMAP-transformed drop data.

Feature	***Mean Absolute SHAP Value***	***Spearman Correlation***
Event Velocity (mm/s)	0.153	0.193
Estimated height at event start (mm)	0.110	0.590
Estimated height at event trigger (mm)	0.109	0.483
Maximum jerk (mm/s^3)	0.057	0.081
Initial velocity (mm/s)	0.046	-0.196
Absolute initial velocity (mm/s)	0.044	-0.156
Maximum velocity (mm/s)	0.041	0.458
Initial vertical velocity (mm/s)	0.040	-0.208
Vertical displacement (mm)	0.027	0.633
Maximum acceleration (mm/s^2)	0.024	0.128
Event duration (s)	0.011	0.909
Estimated height at event end (mm)	0.009	0.796
Event duration (frames)	0.008	0.849
Average velocity (mm/s)	0.006	0.532
Estimated X position at event end (mm)	0.001	-0.129
Estimated X position at event start (mm)	0.001	-0.168
Horizontal displacement (mm)	0.001	-0.294
Event angle (degrees)	0.001	-0.028

Supplementary Table S5. Mean absolute SHAP values and Spearman correlation of features for Cluster 1, as defined by HDBSCAN clustering on UMAP-transformed drop data.

Feature	*Mean Absolute SHAP Value*	*Spearman Correlation*
Event Velocity (mm/s)	0.179	-0.077
Maximum jerk (mm/s^3)	0.060	-0.072
Maximum velocity (mm/s)	0.034	-0.815
Estimated height at event start (mm)	0.025	0.449
Estimated height at event trigger (mm)	0.022	0.551
Initial velocity (mm/s)	0.014	-0.439
Maximum acceleration (mm/s^2)	0.012	-0.911
Absolute initial velocity (mm/s)	0.012	-0.450
Vertical displacement (mm)	0.011	0.711
Initial vertical velocity (mm/s)	0.009	-0.313
Event duration (s)	0.006	-0.438
Event duration (frames)	0.006	-0.272
Average velocity (mm/s)	0.005	-0.928
Horizontal displacement (mm)	0.001	-0.692
Estimated height at event end (mm)	0.001	-0.030
Estimated X position at event end (mm)	0.001	-0.639
Event angle (degrees)	0.001	-0.834
Estimated X position at event start (mm)	0.000	-0.425

Supplementary Table S6. Comparing event counts and drop metrics of 12-day-old *w[1118]* males (n = 6) to 12-day-old *w[1118]* females (n = 6). *p*-value for significance is $p < 0.05$.

	w[1118] male			*w[1118]* female				
**Metric**	**Mean**	**SD**	**Median**	**Mean**	**SD**	**Median**	***p* value**	**Test**
Event Counts								
Upward Jumps	0	0	0	0.167	0.373	0	0.405	Mann–Whitney U test
Arcjumps	0.167	0.373	0	0	0	0	0.405	Mann–Whitney U test
Falls	6.167	4.74	6.5	4.833	3.484	4.5	0.623	Student's t-test
Downward Jumps	0.167	0.373	0	0.5	0.5	0.5	0.282	Mann–Whitney U test
Drops	6.333	4.853	6.5	5.333	3.399	4.5	0.714	Student's t-test
Total Events	6.5	4.682	6.5	5.5	3.202	4.5	0.702	Student's t-test
Total Movement (mm)	6.72E+03	3.20E+03	7.84E+03	7.04E+03	1.96E+03	6.42E+03	0.589	Mann–Whitney U test
Time spent in upper half of vial	0.564	0.264	0.646	0.664	0.085	0.686	0.937	Mann–Whitney U test
Event-Movement ratio	7.74E-04	5.12E-04	8.13E-04	8.21E-04	5.28E-04	6.01E-04	0.888	Student's t-test
Drop Metrics								
Event duration (frames)	4.457	0.455	4.286	3.66	1.314	4	0.272	Student's t-test
Event duration (s)	0.077	7.85E-03	0.074	0.063	0.023	0.069	0.272	Student's t-test
Vertical displacement (mm)	23.476	5.75	25.882	25.474	3.232	25.024	0.662	Mann–Whitney U test
Horizontal displacement (mm)	2.982	1.295	2.591	4.318	1.557	3.885	0.2	Student's t-test
Estimated height at event trigger (mm)	14.933	8.784	12.412	13.9	5.226	15.128	0.832	Student's t-test
Estimated height at event start (mm)	13.548	7.24	11.231	13.124	4.788	14.478	0.792	Mann–Whitney U test
Estimated X position at event start (mm)	23.007	0.751	22.71	20.92	1.339	20.821	0.021	Student's t-test
Estimated height at event end (mm)	37.024	1.781	37.024	38.599	3.275	38.838	0.406	Student's t-test
Estimated X position at event end (mm)	22.358	1.388	22.284	22.603	2.824	21.302	0.877	Student's t-test
Event velocity (mm/s)	558.012	194.892	602.501	774.302	513.395	571.98	1	Mann–Whitney U test
Maximum velocity (mm/s)	435.779	97.165	471.276	811.842	519.639	593.409	0.329	Mann–Whitney U test
Average velocity (mm/s)	262.357	51.382	267.914	609.555	581.118	349.959	0.126	Mann–Whitney U test
Initial vertical velocity (mm/s)	280.18	63.868	306.322	592.202	576.187	357.159	0.429	Mann–Whitney U test
Maximum acceleration (mm/s^2)	1.86E+04	4.21E+03	2.11E+04	3.90E+04	3.31E+04	2.59E+04	0.329	Mann–Whitney U test
Initial velocity (mm/s)	286.165	62.96	309.468	612.596	583.52	373.148	0.329	Mann–Whitney U test
Absolute initial velocity (mm/s)	286.165	62.96	309.468	612.596	583.52	373.148	0.329	Mann–Whitney U test
Maximum jerk (mm/s^3)	1.00E+06	7.11E+05	1.15E+06	8.04E+05	6.56E+05	6.95E+05	0.675	Student's t-test
Lag time (s)	1.437	0.446	1.152	2.824	1.899	2.291	0.183	Student's t-test
Event Angle (degrees)	94.663	7.784	92.287	87.995	8.26	86.373	0.247	Mann–Whitney U test

Supplementary Table S7. Comparing event counts and drop metrics of 11-day-old *w[1118]* males (n = 6) to 11-day-old *w[1118]* females (n = 6). *p*-value for significance is $p < 0.05$.

	***w[1118]* male**			***w[1118]* female**				
**Metric**	**Mean**	**SD**	**Median**	**Mean**	**SD**	**Median**	***p* value**	**Test**
Event Counts								
Upward Jumps	0	0	0	0.167	0.373	0	0.405	Mann–Whitney U test
Arcjumps	0.5	1.118	0	0.167	0.373	0	1	Mann–Whitney U test
Falls	14.833	13.631	8.5	9.667	5.706	9.5	1	Mann–Whitney U test
Downward Jumps	0.167	0.373	0	0.333	0.745	0	1	Mann–Whitney U test
Drops	15	13.54	8.5	10	6.377	9.5	1	Mann–Whitney U test
Total Events	15.5	13.66	8.5	10.333	6.236	10	1	Mann–Whitney U test
Total Movement (mm)	1.12E+04	855.834	1.13E+04	9.09E+03	3.35E+03	1.07E+04	0.209	Student's t-test
Time spent in upper half of vial	0.657	0.073	0.642	0.647	0.08	0.683	0.846	Student's t-test
Event-Movement ratio	1.38E-03	1.22E-03	8.06E-04	1.11E-03	4.49E-04	9.15E-04	0.394	Mann–Whitney U test
Drop Metrics								
Event duration (frames)	4.754	1.099	5.293	4.462	0.738	4.667	0.632	Student's t-test
Event duration (s)	0.082	0.019	0.091	0.077	0.013	0.08	0.632	Student's t-test
Vertical displacement (mm)	25.474	4.228	26.883	22.448	4.816	22.923	0.316	Student's t-test
Horizontal displacement (mm)	4.028	1.602	4.558	3.38	1.012	3.714	0.462	Student's t-test
Estimated height at event trigger (mm)	12.513	5.079	9.749	16.338	7.01	14.78	0.394	Mann–Whitney U test
Estimated height at event start (mm)	11.956	4.867	9.291	14.864	6.086	13.498	0.485	Mann–Whitney U test
Estimated X position at event start (mm)	20.695	1.939	20.056	21.769	4.895	21.4	0.818	Mann–Whitney U test
Estimated height at event end (mm)	37.43	1.716	38.037	37.312	3.565	37.891	0.949	Student's t-test
Estimated X position at event end (mm)	20.27	1.434	19.728	20.843	3.607	20.739	0.752	Welch's t-test
Event velocity (mm/s)	463.231	66.297	476.861	379.634	115.491	362.804	0.191	Student's t-test
Maximum velocity (mm/s)	512.858	62.908	518.83	524.218	121.755	494.254	1	Mann–Whitney U test
Average velocity (mm/s)	303.466	22.943	314.178	295.517	72.421	271.242	0.82	Student's t-test
Initial vertical velocity (mm/s)	237.308	61.404	211.797	286.139	58.05	277.638	0.225	Student's t-test
Maximum acceleration (mm/s^2)	2.00E+04	3.62E+03	1.91E+04	2.19E+04	7.17E+03	2.01E+04	0.611	Student's t-test
Initial velocity (mm/s)	261.224	49.732	240.974	295.293	61.784	281.384	0.359	Student's t-test
Absolute initial velocity (mm/s)	261.224	49.732	240.974	295.293	61.784	281.384	0.359	Student's t-test
Maximum jerk (mm/s^3)	1.05E+06	4.70E+05	1.11E+06	1.47E+06	5.65E+05	1.23E+06	0.232	Student's t-test
Lag time (s)	1.824	0.559	1.819	2.373	1.081	2.882	0.337	Student's t-test
Event Angle (degrees)	92.833	2.837	93.391	93.777	4.074	93.998	0.68	Student's t-test

Supplementary Table S8. Comparing event counts and drop metrics of 13-day-old *w[1118]* males (n = 6) to 13-day-old *w[1118]* females (n = 6). *p*-value for significance is $p < 0.05$.

	***w[1118]* male**			***w[1118]* female**				
**Metric**	**Mean**	**SD**	**Median**	**Mean**	**SD**	**Median**	***p* value**	**Test**
Event Counts								
Upward Jumps	0	0	0	0	0	0	-	Welch's t-test
Arcjumps	0	0	0	0.167	0.373	0	0.405	Mann–Whitney U test
Falls	6.333	2.867	5.5	3.833	1.772	3.5	0.128	Student's t-test
Downward Jumps	0.167	0.373	0	0.167	0.373	0	1	Mann–Whitney U test
Drops	6.5	3.096	5.5	4	1.732	4	0.146	Student's t-test
Total Events	6.5	3.096	5.5	4.167	2.034	4	0.189	Student's t-test
Total Movement (mm)	8.96E+03	2.22E+03	9.84E+03	5.45E+03	3.87E+03	5.00E+03	0.109	Student's t-test
Time spent in upper half of vial	0.599	0.131	0.614	0.646	0.099	0.652	0.538	Student's t-test
Event-Movement ratio	7.78E-04	3.54E-04	8.55E-04	1.02E-03	3.95E-04	1.13E-03	0.332	Student's t-test
Drop Metrics								
Event duration (frames)	4.686	0.859	4.682	5.514	1.609	4.875	0.688	Mann–Whitney U test
Event duration (s)	0.081	0.015	0.081	0.095	0.028	0.084	0.699	Mann–Whitney U test
Vertical displacement (mm)	21.669	4.436	21.072	28.774	4.968	31.4	0.038	Student's t-test
Horizontal displacement (mm)	3.428	1.21	3.052	3	1.064	3.143	0.566	Student's t-test
Estimated height at event trigger (mm)	15.252	4.903	15.5	10.614	6.355	8.156	0.18	Mann–Whitney U test
Estimated height at event start (mm)	14.382	4.206	14.605	10.114	5.475	8.13	0.18	Mann–Whitney U test
Estimated X position at event start (mm)	23.097	2.031	23.767	22.839	5.887	24.69	0.929	Welch's t-test
Estimated height at event end (mm)	36.05	3.175	36.208	38.889	3.458	39.111	0.206	Student's t-test
Estimated X position at event end (mm)	21.704	1.918	21.756	22.215	5.047	23.768	0.837	Student's t-test
Event velocity (mm/s)	458.291	165.729	434.016	555.179	157.726	562.539	0.366	Student's t-test
Maximum velocity (mm/s)	403.378	115.733	395.524	488.858	324.917	357.85	0.592	Student's t-test
Average velocity (mm/s)	230.482	34.451	240.482	270.051	123.94	239.492	0.518	Welch's t-test
Initial vertical velocity (mm/s)	215.304	38.736	220.267	252.266	115.205	252.397	0.521	Welch's t-test
Maximum acceleration (mm/s^2)	1.84E+04	5.45E+03	1.74E+04	1.97E+04	1.05E+04	1.64E+04	0.815	Student's t-test
Initial velocity (mm/s)	223.152	39.186	229.023	258.689	114.268	259.978	0.534	Welch's t-test
Absolute initial velocity (mm/s)	223.152	39.186	229.023	258.689	114.268	259.978	0.534	Welch's t-test
Maximum jerk (mm/s^3)	1.21E+06	7.43E+05	1.12E+06	1.11E+06	1.11E+06	7.07E+05	0.861	Student's t-test
Lag time (s)	2.22	1.445	1.602	5.348	6.143	1.309	0.937	Mann–Whitney U test
Event Angle (degrees)	92.407	5.309	92.114	90.611	2.1	90.681	0.498	Student's t-test

Supplementary Table S9. Comparing event counts and drop metrics of 52-day-old *w[1118]* males (n = 8) to 52-day-old *w[1118]* females (n = 8). *p*-value for significance is $p < 0.05$.

	***w[1118]* male**			***w[1118]* female**				
**Metric**	**Mean**	**SD**	**Median**	**Mean**	**SD**	**Median**	***p* value**	**Test**
Event Counts								
Upward Jumps	0	0	0	0.25	0.433	0	0.17	Mann–Whitney U test
Arcjumps	0.125	0.331	0	0.125	0.331	0	1	Mann–Whitney U test
Falls	0.875	1.364	0	1.75	0.829	2	0.096	Mann–Whitney U test
Downward Jumps	0.25	0.661	0	0.5	0.707	0	0.369	Mann–Whitney U test
Drops	1.125	1.691	0	2.25	1.392	2	0.143	Mann–Whitney U test
Total Events	1.25	1.639	0.5	2.625	1.867	2	0.146	Mann–Whitney U test
Total Movement (mm)	1.60E+03	631.533	1.89E+03	2.80E+03	2.33E+03	2.23E+03	0.225	Welch's t-test
Time spent in upper half of vial	0.405	0.232	0.398	0.561	0.268	0.595	0.264	Student's t-test
Event-Movement ratio	8.57E-04	1.10E-03	2.60E-04	1.23E-03	9.96E-04	8.79E-04	0.365	Mann–Whitney U test
Drop Metrics								
Event duration (frames)	5.667	1.65	4.5	3.667	1.08	4	0.169	Mann–Whitney U test
Event duration (s)	0.098	0.028	0.078	0.063	0.019	0.069	0.183	Mann–Whitney U test
Vertical displacement (mm)	27.488	6.498	29.455	19.456	9.88	20.849	0.281	Student's t-test
Horizontal displacement (mm)	5.19	2.365	6.63	4.055	2.014	3.969	0.509	Student's t-test
Estimated height at event trigger (mm)	11.819	4.373	9.533	15.064	10.658	11.358	1	Mann–Whitney U test
Estimated height at event start (mm)	9.744	1.873	9.533	13.944	8.199	11.136	0.833	Mann–Whitney U test
Estimated X position at event start (mm)	23.263	5.435	25.828	23.646	3.805	21.753	1	Mann–Whitney U test
Estimated height at event end (mm)	37.232	7.788	41.591	33.399	10.313	37.452	0.383	Mann–Whitney U test
Estimated X position at event end (mm)	23.366	2.312	22.335	21.511	4.503	20.989	0.562	Student's t-test
Event velocity (mm/s)	487.603	112.493	516.997	473.863	206.447	435.376	0.925	Student's t-test
Maximum velocity (mm/s)	971.227	466.284	753.031	775.328	225.361	744.96	0.447	Student's t-test
Average velocity (mm/s)	438.166	196.271	405.257	492.176	199.922	435.941	0.734	Student's t-test
Initial vertical velocity (mm/s)	317.952	253.453	218.948	405.23	208.759	366.779	0.626	Student's t-test
Maximum acceleration (mm/s^2)	4.89E+04	3.16E+04	4.04E+04	3.81E+04	1.32E+04	3.25E+04	0.514	Student's t-test
Initial velocity (mm/s)	374.06	240.003	372.409	490.722	194.072	448.325	0.49	Student's t-test
Absolute initial velocity (mm/s)	374.06	240.003	372.409	490.722	194.072	448.325	0.49	Student's t-test
Maximum jerk (mm/s^3)	3.34E+06	2.65E+06	2.76E+06	2.20E+06	1.52E+06	2.18E+06	0.465	Student's t-test
Lag time (s)	9.021	9.684	2.864	22.415	43.108	2.079	0.833	Mann–Whitney U test
Event Angle (degrees)	95.313	14.651	93.72	99.609	23.108	93.42	0.797	Student's t-test

Supplementary Table S10. Comparing event counts and drop metrics of 40-day-old *w[1118]* males (n = 8) to 40-day-old *w[1118]* females (n = 8). *p*-value for significance is $p < 0.05$.

	***w[1118]* male**			***w[1118]* female**				
**Metric**	**Mean**	**SD**	**Median**	**Mean**	**SD**	**Median**	***p* value**	**Test**
Event Counts								
Upward Jumps	0	0	0	0	0	0	-	Welch's t-test
Arcjumps	0	0	0	0	0	0	-	Welch's t-test
Falls	3	4.637	1.5	3.875	3.551	4	0.708	Mann–Whitney U test
Downward Jumps	0.375	0.696	0	0.375	0.696	0	1	Mann–Whitney U test
Drops	3.375	5.243	2	4.25	4.085	4	0.747	Mann–Whitney U test
Total Events	3.375	5.243	2	4.25	4.085	4	0.747	Mann–Whitney U test
Total Movement (mm)	1.84E+03	871.99	2.00E+03	2.51E+03	1.61E+03	2.78E+03	0.353	Student's t-test
Time spent in upper half of vial	0.441	0.099	0.461	0.389	0.265	0.529	0.636	Student's t-test
Event-Movement ratio	1.25E-03	1.43E-03	9.24E-04	1.21E-03	1.12E-03	1.15E-03	0.957	Mann–Whitney U test
Drop Metrics								
Event duration (frames)	5.583	1.17	5.5	4.693	1.784	4.222	0.393	Student's t-test
Event duration (s)	0.096	0.02	0.095	0.081	0.031	0.073	0.393	Student's t-test
Vertical displacement (mm)	27.692	5.306	30.341	21.415	8.679	23.841	0.216	Student's t-test
Horizontal displacement (mm)	4.43	2.85	3.964	3.807	2.231	2.596	0.727	Student's t-test
Estimated height at event trigger (mm)	12.346	5.534	8.754	17.397	10.798	16.32	1	Mann–Whitney U test
Estimated height at event start (mm)	11.99	5.267	8.501	15.999	9.851	15.445	0.931	Mann–Whitney U test
Estimated X position at event start (mm)	21.333	2.822	21.326	20.307	2.133	19.848	0.56	Student's t-test
Estimated height at event end (mm)	39.682	1.435	39.58	37.414	3.149	37.739	0.187	Student's t-test
Estimated X position at event end (mm)	20.883	3.675	20.987	18.888	4.11	18.57	0.462	Student's t-test
Event velocity (mm/s)	317.823	83.051	299.769	280.963	77.824	295.06	0.512	Student's t-test
Maximum velocity (mm/s)	970.629	392.529	909.862	792.406	313.828	791.43	0.477	Student's t-test
Average velocity (mm/s)	341.575	69.136	328.164	312.893	76.292	309.973	0.569	Student's t-test
Initial vertical velocity (mm/s)	308.894	151.449	241.449	237.019	76.148	190.622	0.406	Student's t-test
Maximum acceleration (mm/s^2)	4.80E+04	2.26E+04	4.66E+04	3.85E+04	1.89E+04	3.48E+04	0.517	Student's t-test
Initial velocity (mm/s)	316.517	149.529	251.104	259.21	70.686	231.164	0.495	Student's t-test
Absolute initial velocity (mm/s)	316.517	149.529	251.104	259.21	70.686	231.164	0.495	Student's t-test
Maximum jerk (mm/s^3)	4.67E+06	2.77E+06	4.31E+06	3.57E+06	2.11E+06	2.99E+06	0.526	Student's t-test
Lag time (s)	10.69	8.639	9.355	13.974	10.171	10.215	0.613	Student's t-test
Event Angle (degrees)	92.9	10.33	92.9	90.595	8.041	89.737	0.721	Student's t-test

Supplementary Table S11. Comparing event counts and drop metrics of 11-13 day old *w[1118]* males (n = 18) to 11-13 day old *w[1118]* females (n = 18). Pooled data from Tables S6-S8. *p*-value for significance is $p < 0.05$.

	***w[1118]* male**			***w[1118]* female**				
**Metric**	**Mean**	**SD**	**Median**	**Mean**	**SD**	**Median**	***p* value**	**Test**
Event Counts								
Upward Jumps	0.000	0.000	0.000	0.111	0.314	0.000	0.163	Mann–Whitney U test
Arcjumps	0.222	0.711	0.000	0.111	0.314	0.000	0.977	Mann–Whitney U test
Falls	9.111	9.410	7.000	6.111	4.736	5.000	0.287	Mann–Whitney U test
Downward Jumps	0.167	0.373	0.000	0.333	0.577	0.000	0.406	Mann–Whitney U test
Drops	9.278	9.409	7.500	6.444	5.002	5.000	0.274	Mann–Whitney U test
Total Events	9.500	9.523	7.500	6.667	4.978	5.500	0.340	Mann–Whitney U test
Total Movement (mm)	8.95E+03	2.93E+03	9.50E+03	7.19E+03	3.50E+03	6.91E+03	0.159	Mann–Whitney U test
Time spent in upper half of vial	0.607	0.179	0.637	0.652	0.089	0.683	0.837	Mann–Whitney U test
Event-Movement ratio	9.77E-04	8.43E-04	8.06E-04	9.84E-04	4.76E-04	9.15E-04	0.558	Mann–Whitney U test
Drop Metrics								
Event duration (frames)	4.588	0.878	4.682	4.597	1.509	4.667	0.830	Mann–Whitney U test
Event duration (s)	0.079	0.015	0.081	0.079	0.026	0.080	0.754	Mann–Whitney U test
Vertical displacement (mm)	23.419	4.938	24.375	25.817	5.150	24.857	0.181	Student's t-test
Horizontal displacement (mm)	3.658	1.536	3.838	3.411	1.228	3.323	0.614	Student's t-test
Estimated height at event trigger (mm)	14.498	6.412	12.802	13.259	6.690	10.604	0.419	Mann–Whitney U test
Estimated height at event start (mm)	13.577	5.555	11.765	12.352	5.799	9.986	0.400	Mann–Whitney U test
Estimated X position at event start (mm)	22.139	2.027	22.694	21.909	4.679	21.344	0.858	Welch's t-test
Estimated height at event end (mm)	36.996	2.462	36.809	38.169	3.580	38.142	0.278	Student's t-test
Estimated X position at event end (mm)	21.577	1.937	21.860	21.718	4.061	21.833	0.901	Welch's t-test
Event velocity (mm/s)	495.790	154.051	502.506	567.627	366.109	469.709	0.961	Mann–Whitney U test
Maximum velocity (mm/s)	447.444	103.866	459.661	621.872	395.772	493.323	0.204	Mann–Whitney U test
Average velocity (mm/s)	266.253	46.940	270.112	398.451	388.433	279.501	0.204	Mann–Whitney U test
Initial vertical velocity (mm/s)	240.869	59.731	219.909	386.151	382.422	306.397	0.042	Mann–Whitney U test
Maximum acceleration (mm/s^2)	1.88E+04	4.52E+03	1.77E+04	2.76E+04	2.27E+04	2.08E+04	0.419	Mann–Whitney U test
Initial velocity (mm/s)	255.023	55.302	243.071	396.832	389.518	307.511	0.089	Mann–Whitney U test
Absolute initial velocity (mm/s)	255.023	55.302	243.071	396.832	389.518	307.511	0.089	Mann–Whitney U test
Maximum jerk (mm/s^3)	1.05E+06	6.60E+05	1.06E+06	1.17E+06	8.59E+05	9.55E+05	0.654	Student's t-test
Lag time (s)	2.122	1.483	1.662	3.326	4.022	2.146	0.754	Mann–Whitney U test
Event Angle (degrees)	93.367	5.488	92.508	90.497	5.985	89.763	0.204	Mann–Whitney U test

Supplementary Table S12. Comparing event counts and drop metrics of 40-52 day old *w[1118]* males (n = 16) to 40-52 day old *w[1118]* females (n = 16). Pooled data from Tables S9-S10. *p*-value for significance is $p < 0.05$.

	***w[1118]* male**			***w[1118]* female**				
**Metric**	**Mean**	**SD**	**Median**	**Mean**	**SD**	**Median**	***p* value**	**Test**
Event Counts								
Upward Jumps	0.000	0.000	0.000	0.125	0.331	0.000	0.164	Mann–Whitney U test
Arcjumps	0.062	0.242	0.000	0.062	0.242	0.000	1.000	Mann–Whitney U test
Falls	1.938	3.579	1.000	2.812	2.789	2.000	0.155	Mann–Whitney U test
Downward Jumps	0.312	0.682	0.000	0.438	0.704	0.000	0.502	Mann–Whitney U test
Drops	2.250	4.054	1.000	3.250	3.211	2.000	0.169	Mann–Whitney U test
Total Events	2.312	4.027	1.000	3.438	3.278	2.000	0.164	Mann–Whitney U test
Total Movement (mm)	1.72E+03	770.595	1.95E+03	2.65E+03	2.01E+03	2.62E+03	0.109	Welch's t-test
Time spent in upper half of vial	0.423	0.179	0.426	0.475	0.280	0.545	0.547	Student's t-test
Event-Movement ratio	1.05E-03	1.29E-03	8.60E-04	1.22E-03	1.06E-03	8.79E-04	0.553	Mann–Whitney U test
Drop Metrics								
Event duration (frames)	4.692	1.617	4.500	4.911	1.634	4.222	0.805	Student's t-test
Event duration (s)	0.081	0.028	0.078	0.085	0.028	0.073	0.805	Student's t-test
Vertical displacement (mm)	22.540	9.520	24.279	26.248	5.338	30.004	0.439	Student's t-test
Horizontal displacement (mm)	4.460	2.405	4.146	3.641	2.357	2.429	0.533	Student's t-test
Estimated height at event trigger (mm)	15.060	9.989	10.225	12.199	4.118	11.547	0.780	Mann–Whitney U test
Estimated height at event start (mm)	13.829	8.387	10.225	11.500	3.849	11.136	0.660	Mann–Whitney U test
Estimated X position at event start (mm)	21.912	3.799	21.326	22.850	3.651	20.762	0.649	Student's t-test
Estimated height at event end (mm)	36.369	8.163	38.726	37.748	3.099	37.739	0.780	Mann–Whitney U test
Estimated X position at event end (mm)	20.827	3.775	21.091	21.436	5.197	22.153	0.788	Student's t-test
Event velocity (mm/s)	401.789	182.902	361.466	332.595	67.137	347.093	0.440	Student's t-test
Maximum velocity (mm/s)	869.971	383.272	767.713	841.449	225.654	791.430	0.882	Student's t-test
Average velocity (mm/s)	410.761	185.553	393.163	360.295	54.367	375.237	0.575	Student's t-test
Initial vertical velocity (mm/s)	335.335	206.500	243.195	292.713	110.141	312.638	0.905	Mann–Whitney U test
Maximum acceleration (mm/s^2)	4.44E+04	2.24E+04	4.11E+04	3.68E+04	1.66E+04	3.48E+04	0.515	Student's t-test
Initial velocity (mm/s)	389.014	208.786	342.968	305.631	102.199	325.670	0.423	Student's t-test
Absolute initial velocity (mm/s)	389.014	208.786	342.968	305.631	102.199	325.670	0.423	Student's t-test
Maximum jerk (mm/s^3)	3.38E+06	2.59E+06	2.57E+06	3.45E+06	1.92E+06	2.99E+06	0.955	Student's t-test
Lag time (s)	10.132	10.354	5.505	31.172	47.877	6.120	0.445	Mann–Whitney U test
Event Angle (degrees)	95.879	18.211	93.570	91.902	7.083	92.057	0.655	Student's t-test

Supplementary Table S13. Comparing event counts and drop metrics of 2-day-old *w[1118]* males (n = 3) to 2-day-old dehydrated *w[1118]* males (n = 4), Cohort 1. *p*-value for significance is $p < 0.05$.

	***w[1118]* male**			**Dehydrated *w[1118]* male**				
**Metric**	**Mean**	**SD**	**Median**	**Mean**	**SD**	**Median**	***p* value**	**Test**
Event Counts								
Upward Jumps	0	0	0	0.75	0.433	1	0.102	Mann–Whitney U test
Arcjumps	0	0	0	0	0	0	-	Welch's t-test
Falls	1.333	1.886	0	7	5.196	6	0.208	Mann–Whitney U test
Downward Jumps	0.333	0.471	0	1.75	1.09	2	0.195	Mann–Whitney U test
Drops	1.667	1.7	1	8.75	5.54	8.5	0.111	Welch's t-test
Total Events	1.667	1.7	1	9.5	5.852	9.5	0.099	Welch's t-test
Total Movement (mm)	2.73E+03	1.93E+03	3.83E+03	6.63E+03	1.97E+03	6.47E+03	0.078	Student's t-test
Time spent in upper half of vial	0.581	0.07	0.58	0.669	0.046	0.665	0.15	Student's t-test
Event-Movement ratio	4.24E-04	4.47E-04	2.30E-04	1.37E-03	7.72E-04	1.31E-03	0.17	Student's t-test
Drop Metrics								
Event duration (frames)	3.5	2.5	3.5	4.678	0.807	4.45	1	Mann–Whitney U test
Event duration (s)	0.06	0.043	0.06	0.081	0.014	0.077	1	Mann–Whitney U test
Vertical displacement (mm)	33.244	0.119	33.244	24.636	3.185	25.419	0.133	Mann–Whitney U test
Horizontal displacement (mm)	1.196	0.832	1.196	4.189	1.37	4.181	0.133	Mann–Whitney U test
Estimated height at event trigger (mm)	6.574	0.697	6.574	10.14	6.514	7.17	1	Mann–Whitney U test
Estimated height at event start (mm)	6.06	0.184	6.06	8.931	5.532	6.277	1	Mann–Whitney U test
Estimated X position at event start (mm)	22.415	2.151	22.415	20.018	3.277	20.416	0.533	Mann–Whitney U test
Estimated height at event end (mm)	39.304	0.303	39.304	33.567	2.629	32.404	0.133	Mann–Whitney U test
Estimated X position at event end (mm)	22.753	1.449	22.753	22.219	1.592	22.204	0.533	Mann–Whitney U test
Event velocity (mm/s)	1.13E+03	795.644	1.13E+03	429.873	152.055	493.54	0.8	Mann–Whitney U test
Maximum velocity (mm/s)	1.33E+03	589.984	1.33E+03	821.73	376.328	766.935	0.533	Mann–Whitney U test
Average velocity (mm/s)	1.14E+03	786.292	1.14E+03	496.188	190.337	552.478	0.8	Mann–Whitney U test
Initial vertical velocity (mm/s)	1.06E+03	860.24	1.06E+03	427.958	168.455	469.316	0.8	Mann–Whitney U test
Maximum acceleration (mm/s^2)	7.30E+04	3.85E+04	7.30E+04	4.16E+04	2.36E+04	3.87E+04	0.8	Mann–Whitney U test
Initial velocity (mm/s)	1.06E+03	856.884	1.06E+03	528.884	247.578	539.517	0.8	Mann–Whitney U test
Absolute initial velocity (mm/s)	1.06E+03	856.884	1.06E+03	528.884	247.578	539.517	0.8	Mann–Whitney U test
Maximum jerk (mm/s^3)	1.47E+06	1.47E+06	1.47E+06	3.06E+06	2.74E+06	1.79E+06	0.8	Mann–Whitney U test
Lag time (s)	3.824	3.179	3.824	1.124	0.469	1.015	0.8	Mann–Whitney U test
Event Angle (degrees)	89.48	1.18	89.48	84.52	3.737	84.373	0.267	Mann–Whitney U test

Supplementary Table S14. Comparing event counts and drop metrics of 2-day-old *w[1118]* males (n = 3) to 2-day-old dehydrated *w[1118]* males (n = 3), Cohort 2. *p*-value for significance is $p < 0.05$.

	***w[1118]* male**			**Dehydrated *w[1118]* male**				
**Metric**	**Mean**	**SD**	**Median**	**Mean**	**SD**	**Median**	***p* value**	**Test**
Event Counts								
Upward Jumps	0	0	0	0	0	0	-	Welch's t-test
Arcjumps	0	0	0	0.333	0.471	0	0.505	Mann–Whitney U test
Falls	13.333	5.793	12	32.333	31.478	22	0.448	Student's t-test
Downward Jumps	0	0	0	1.333	0.943	2	0.188	Mann–Whitney U test
Drops	13.333	5.793	12	33.667	32.17	24	0.429	Student's t-test
Total Events	13.333	5.793	12	34	32.073	25	0.421	Student's t-test
Total Movement (mm)	1.25E+04	492.795	1.24E+04	8.50E+03	5.46E+03	1.23E+04	0.7	Mann–Whitney U test
Time spent in upper half of vial	0.576	0.062	0.534	0.335	0.13	0.418	0.1	Mann–Whitney U test
Event-Movement ratio	1.08E-03	5.02E-04	9.14E-04	2.76E-03	2.62E-03	2.01E-03	0.422	Student's t-test
Drop Metrics								
Event duration (frames)	4.741	0.866	4.45	4.363	0.012	4.363	0.8	Mann–Whitney U test
Event duration (s)	0.082	0.015	0.077	0.075	2.04E-04	0.075	0.8	Mann–Whitney U test
Vertical displacement (mm)	25.752	2.641	25.8	21.893	1.888	21.893	0.4	Mann–Whitney U test
Horizontal displacement (mm)	3.039	0.269	3.018	2.792	0.141	2.792	0.4	Mann–Whitney U test
Estimated height at event trigger (mm)	10.735	2.088	11.489	16.232	1.401	16.232	0.2	Mann–Whitney U test
Estimated height at event start (mm)	9.64	2.217	11.079	14.693	1.828	14.693	0.2	Mann–Whitney U test
Estimated X position at event start (mm)	22.4	1.67	23.358	22.14	0.114	22.14	0.8	Mann–Whitney U test
Estimated height at event end (mm)	35.392	1.248	35.471	36.586	0.059	36.586	0.8	Mann–Whitney U test
Estimated X position at event end (mm)	21.855	0.824	21.589	21.736	0.168	21.736	1	Mann–Whitney U test
Event velocity (mm/s)	385.251	65.962	416.64	373.639	44.515	373.639	1	Mann–Whitney U test
Maximum velocity (mm/s)	617.836	71.764	637.393	578.597	48.59	578.597	0.8	Mann–Whitney U test
Average velocity (mm/s)	322.054	20.898	315.673	348.049	86.284	348.049	1	Mann–Whitney U test
Initial vertical velocity (mm/s)	248.499	28.557	265.232	307.778	81.066	307.778	0.8	Mann–Whitney U test
Maximum acceleration (mm/s^2)	2.58E+04	3.12E+03	2.68E+04	2.49E+04	1.03E+03	2.49E+04	0.8	Mann–Whitney U test
Initial velocity (mm/s)	261.342	23.718	275.962	326.524	87.019	326.524	0.8	Mann–Whitney U test
Absolute initial velocity (mm/s)	261.342	23.718	275.962	326.524	87.019	326.524	0.8	Mann–Whitney U test
Maximum jerk (mm/s^3)	1.97E+06	3.65E+05	2.07E+06	1.60E+06	3.14E+05	1.60E+06	0.4	Mann–Whitney U test
Lag time (s)	1.123	0.074	1.119	0.841	0.024	0.841	0.2	Mann–Whitney U test
Event Angle (degrees)	88.35	2.99	86.413	90.198	0.022	90.198	0.8	Mann–Whitney U test

Supplementary Table S15. Comparing event counts and drop metrics of 7-day-old control females (n = 4) to 7-day-old *Abeta* females (n = 4). *p*-value for significance is $p < 0.05$.

	Control female			*Abeta* female				
**Metric**	**Mean**	**SD**	**Median**	**Mean**	**SD**	**Median**	***p* value**	**Test**
Event Counts								
Upward Jumps	0.000	0	0	0.5	0.5	0.5	0.181	Mann–Whitney U test
Arcjumps	0.75	1.299	0	0	0	0	0.453	Mann–Whitney U test
Falls	13.75	11.691	9.5	7.5	6.344	7	0.447	Student's t-test
Downward Jumps	0.5	0.866	0	0.75	0.829	0.5	0.739	Mann–Whitney U test
Drops	14.25	12.517	9.5	8.25	6.18	8.5	0.485	Student's t-test
Total Events	15	13.766	9.5	8.75	6.22	9.5	0.501	Student's t-test
Total Movement (mm)	8.47E+03	3.32E+03	6.83E+03	4.24E+03	2.49E+03	5.20E+03	0.057	Mann–Whitney U test
Time spent in upper half of vial	0.666	0.154	0.652	0.649	0.119	0.645	0.887	Student's t-test
Event-Movement ratio	1.50E-03	8.56E-04	1.45E-03	1.63E-03	1.23E-03	1.70E-03	0.892	Student's t-test
Drop Metrics								
Event duration (frames)	4.506	1.126	4.833	4.525	0.971	4.875	0.985	Student's t-test
Event duration (s)	0.078	0.019	0.083	0.078	0.017	0.084	0.985	Student's t-test
Vertical displacement (mm)	23.086	4.507	22.924	28.236	1.366	28.035	0.167	Student's t-test
Horizontal displacement (mm)	4.212	2.347	3.727	3.407	1.063	3.59	0.66	Student's t-test
Estimated height at event trigger (mm)	12.232	3.757	11.415	8.662	0.948	9.025	0.232	Student's t-test
Estimated height at event start (mm)	10.979	4.103	9.662	7.145	0.411	7.352	0.231	Student's t-test
Estimated X position at event start (mm)	17.638	3.763	19.189	16.756	3.271	17.344	0.795	Student's t-test
Estimated height at event end (mm)	34.065	3.304	34.681	35.381	1.743	35.387	0.619	Student's t-test
Estimated X position at event end (mm)	16.819	2.492	16.379	15.059	2.757	15.32	0.489	Student's t-test
Event velocity (mm/s)	408.704	131.198	393.551	489.437	193.965	363.034	0.603	Student's t-test
Maximum velocity (mm/s)	599.582	87.401	581.815	773.807	109.443	717.401	0.105	Student's t-test
Average velocity (mm/s)	364.465	98.825	334.12	502.199	190.845	367.422	0.229	Mann–Whitney U test
Initial vertical velocity (mm/s)	379.949	80.139	373.487	459.357	196.516	342.022	0.564	Student's t-test
Maximum acceleration (mm/s^2)	2.64E+04	4.41E+03	2.59E+04	3.64E+04	9.46E+03	3.04E+04	0.174	Student's t-test
Initial velocity (mm/s)	410.284	88.87	376.844	484.466	183.941	411.106	0.578	Student's t-test
Absolute initial velocity (mm/s)	410.284	88.87	376.844	484.466	183.941	411.106	0.578	Student's t-test
Maximum jerk (mm/s^3)	1.55E+06	5.60E+05	1.54E+06	2.18E+06	4.59E+05	2.32E+06	0.238	Student's t-test
Lag time (s)	1.658	0.298	1.67	1.218	0.162	1.108	0.114	Mann–Whitney U test
Event Angle (degrees)	90.341	9.322	92.206	92.768	0.827	92.649	0.719	Student's t-test

Supplementary Table S16. Comparing event counts and drop metrics of 7-day-old control males (n = 8) to 7-day-old *Abeta* males (n = 7), Cohort 1. *p*-value for significance is $p < 0.05$.

	**Control male**			***Abeta* male**				
**Metric**	**Mean**	**SD**	**Median**	**Mean**	**SD**	**Median**	***p* value**	**Test**
Event Counts								
Upward Jumps	0.625	1.317	0	0.125	0.331	0	0.537	Mann–Whitney U test
Arcjumps	0.875	1.615	0	0	0	0	0.076	Mann–Whitney U test
Falls	10.25	7.806	9.5	15.75	26.447	5	0.674	Mann–Whitney U test
Downward Jumps	0.625	0.992	0	2.625	4.608	0	0.717	Mann–Whitney U test
Drops	10.875	8.536	10	18.375	30.883	5	0.874	Mann–Whitney U test
Total Events	12.375	11.157	10.5	18.5	30.826	5.5	0.792	Mann–Whitney U test
Total Movement (mm)	5.00E+03	1.18E+03	5.11E+03	5.24E+03	3.75E+03	3.89E+03	0.874	Student's t-test
Time spent in upper half of vial	0.531	0.063	0.53	0.377	0.193	0.411	0.064	Student's t-test
Event-Movement ratio	2.46E-03	1.80E-03	2.26E-03	2.64E-03	2.13E-03	1.84E-03	0.959	Mann–Whitney U test
Drop Metrics								
Event duration (frames)	4.291	0.472	4.204	4.425	0.948	4.381	0.743	Student's t-test
Event duration (s)	0.074	8.14E-03	0.072	0.076	0.016	0.076	0.743	Student's t-test
Vertical displacement (mm)	24.053	4.463	25.423	23.43	8.346	24.023	0.864	Student's t-test
Horizontal displacement (mm)	3.502	1.143	3.783	2.865	1.045	2.836	0.295	Student's t-test
Estimated height at event trigger (mm)	12.166	3.514	11.787	14.566	7.806	13.095	0.645	Mann–Whitney U test
Estimated height at event start (mm)	10.286	3.557	9.413	12.28	7.565	11.354	0.959	Mann–Whitney U test
Estimated X position at event start (mm)	20.169	5.764	20.167	19.212	4.723	19.728	0.739	Student's t-test
Estimated height at event end (mm)	34.339	2.668	35.302	35.711	1.76	35.564	0.275	Student's t-test
Estimated X position at event end (mm)	18.71	6.015	17.899	18.729	5.11	19.201	0.995	Student's t-test
Event velocity (mm/s)	389.101	68.577	408.725	340.449	101.256	333.586	0.31	Student's t-test
Maximum velocity (mm/s)	631.816	145.766	639.672	622.29	268.432	636.99	0.936	Welch's t-test
Average velocity (mm/s)	392.28	116.914	364.112	316.571	88.034	332.96	0.328	Mann–Whitney U test
Initial vertical velocity (mm/s)	307.159	89.41	281.626	318.627	118.529	288.82	0.841	Student's t-test
Maximum acceleration (mm/s^2)	2.83E+04	1.06E+04	2.53E+04	2.91E+04	1.38E+04	2.78E+04	0.912	Student's t-test
Initial velocity (mm/s)	373.375	137.546	325.702	330.275	114.157	294.356	0.534	Student's t-test
Absolute initial velocity (mm/s)	373.375	137.546	325.702	330.275	114.157	294.356	0.534	Student's t-test
Maximum jerk (mm/s^3)	2.21E+06	1.15E+06	1.83E+06	2.42E+06	1.43E+06	2.20E+06	0.772	Student's t-test
Lag time (s)	3.274	2.759	2.297	4.455	4.302	2.061	0.959	Mann–Whitney U test
Event Angle (degrees)	94.043	5.174	92.271	93.255	5.62	93.362	0.959	Mann–Whitney U test

Supplementary Table S17. Comparing event counts and drop metrics of 7-day-old control males (n = 4) to 7-day-old *Abeta* males (n = 4), Cohort 2. *p*-value for significance is $p < 0.05$.

	**Control male**			***Abeta* male**				
**Metric**	**Mean**	**SD**	**Median**	**Mean**	**SD**	**Median**	***p* value**	**Test**
Event Counts								
Upward Jumps	0	0	0	0	0	0	-	Welch's t-test
Arcjumps	1.25	2.165	0	0.25	0.433	0	1	Mann–Whitney U test
Falls	11.75	3.961	13.5	14	5.339	13.5	0.579	Student's t-test
Downward Jumps	1.25	1.09	1	0.75	0.829	0.5	0.55	Student's t-test
Drops	13	4.743	15	14.75	6.057	14	0.707	Student's t-test
Total Events	14.25	6.098	15	15	6.442	14	0.888	Student's t-test
Total Movement (mm)	6.80E+03	893.383	6.55E+03	9.00E+03	2.83E+03	7.69E+03	0.245	Student's t-test
Time spent in upper half of vial	0.578	0.055	0.597	0.668	0.083	0.689	0.17	Student's t-test
Event-Movement ratio	2.20E-03	1.00E-03	2.46E-03	1.64E-03	4.13E-04	1.77E-03	0.402	Student's t-test
Drop Metrics								
Event duration (frames)	5.203	0.57	5.259	4.49	0.317	4.479	0.107	Student's t-test
Event duration (s)	0.09	9.83E-03	0.091	0.077	5.47E-03	0.077	0.107	Student's t-test
Vertical displacement (mm)	28.435	2.364	28.52	25.24	2.302	25.237	0.145	Student's t-test
Horizontal displacement (mm)	3.45	0.854	3.18	4.616	0.731	4.848	0.123	Student's t-test
Estimated height at event trigger (mm)	9.024	3.342	8.012	10.79	1.779	11.084	0.45	Student's t-test
Estimated height at event start (mm)	8.062	3.089	7.093	9.175	1.803	9.391	0.609	Student's t-test
Estimated X position at event start (mm)	22.35	0.931	22.16	21.186	1.564	20.732	0.31	Student's t-test
Estimated height at event end (mm)	36.497	1.588	37.116	34.415	2.791	33.188	0.304	Student's t-test
Estimated X position at event end (mm)	22.515	1.384	22.034	21.036	0.961	21.116	0.179	Student's t-test
Event velocity (mm/s)	411.501	16.271	408.691	429.848	60.276	455.261	0.629	Student's t-test
Maximum velocity (mm/s)	820.905	104.99	813.906	632.475	65.087	653.235	0.038	Student's t-test
Average velocity (mm/s)	447.627	30.063	447.543	400.642	59.597	389.755	0.269	Student's t-test
Initial vertical velocity (mm/s)	401.178	65.357	387.71	325.659	65.13	324.715	0.206	Student's t-test
Maximum acceleration (mm/s^2)	3.84E+04	8.99E+03	4.12E+04	2.53E+04	4.25E+03	2.46E+04	0.063	Student's t-test
Initial velocity (mm/s)	409.314	62.744	392.759	352.611	68.817	368.823	0.332	Student's t-test
Absolute initial velocity (mm/s)	409.314	62.744	392.759	352.611	68.817	368.823	0.332	Student's t-test
Maximum jerk (mm/s^3)	2.21E+06	1.15E+06	1.83E+06	2.42E+06	1.43E+06	2.20E+06	0.772	Student's t-test
Lag time (s)	3.274	2.759	2.297	4.455	4.302	2.061	0.959	Mann–Whitney U test
Event Angle (degrees)	94.043	5.174	92.271	93.255	5.62	93.362	0.959	Mann–Whitney U test

Supplementary Table S18. Comparing event counts and drop metrics of 7-day-old control males (n = 12) to 7-day-old *Abeta* males (n = 11). Pooled data from Tables S16 and S17. *p*-value for significance is $p < 0.05$.

	**Control male**			***Abeta* male**				
**Metric**	**Mean**	**SD**	**Median**	**Mean**	**SD**	**Median**	***p* value**	**Test**
Event Counts								
Upward Jumps	0	0	0	0.5	0.5	0.5	0.181	Mann–Whitney U test
Arcjumps	0.417	1.115	0.000	0.083	0.276	0.000	0.546	Mann–Whitney U test
Falls	1.000	1.826	0.000	0.083	0.276	0.000	0.132	Mann–Whitney U test
Downward Jumps	10.750	6.808	12.500	15.167	21.828	8.000	0.817	Mann–Whitney U test
Drops	0.833	1.067	0.500	2.000	3.894	0.000	0.975	Mann–Whitney U test
Total Events	11.583	7.555	13.000	17.167	25.514	10.500	0.954	Mann–Whitney U test
Total Movement (mm)	13.000	9.806	13.500	17.333	25.496	10.500	0.816	Mann–Whitney U test
Time spent in upper half of vial	5.60E+03	1.38E+03	5.66E+03	6.50E+03	3.90E+03	7.06E+03	0.485	Welch's t-test
Event-Movement ratio	0.547	0.064	0.560	0.474	0.215	0.513	0.300	Welch's t-test
Drop Metrics								
Event duration (frames)	4.595	0.665	4.561	4.447	0.796	4.381	0.639	Student's t-test
Event duration (s)	0.079	0.011	0.079	0.077	0.014	0.076	0.639	Student's t-test
Vertical displacement (mm)	25.514	4.405	25.889	24.033	6.995	24.023	0.583	Mann–Whitney U test
Horizontal displacement (mm)	3.485	1.056	3.455	3.449	1.260	3.420	0.943	Student's t-test
Estimated height at event trigger (mm)	11.119	3.762	10.848	13.307	6.697	12.519	0.470	Mann–Whitney U test
Estimated height at event start (mm)	9.544	3.566	8.871	11.245	6.433	10.238	0.707	Mann–Whitney U test
Estimated X position at event start (mm)	20.896	4.848	22.160	19.870	4.068	20.732	0.596	Student's t-test
Estimated height at event end (mm)	35.058	2.573	35.888	35.279	2.244	35.360	0.832	Student's t-test
Estimated X position at event end (mm)	19.978	5.290	21.632	19.498	4.347	21.093	0.818	Student's t-test
Event velocity (mm/s)	396.568	57.749	408.691	370.249	99.108	368.318	0.455	Student's t-test
Maximum velocity (mm/s)	694.846	160.577	676.847	625.685	222.424	653.235	0.412	Student's t-test
Average velocity (mm/s)	410.729	100.472	399.518	344.594	89.001	342.373	0.116	Student's t-test
Initial vertical velocity (mm/s)	338.499	93.369	335.143	320.971	103.880	307.218	0.681	Student's t-test
Maximum acceleration (mm/s^2)	3.17E+04	1.11E+04	2.91E+04	2.78E+04	1.17E+04	2.46E+04	0.437	Student's t-test
Initial velocity (mm/s)	385.355	119.214	369.365	337.720	101.870	325.985	0.325	Student's t-test
Absolute initial velocity (mm/s)	385.355	119.214	369.365	337.720	101.870	325.985	0.325	Student's t-test
Maximum jerk (mm/s^3)	2.40E+06	1.08E+06	2.09E+06	2.06E+06	1.28E+06	1.51E+06	0.312	Mann–Whitney U test
Lag time (s)	3.370	2.357	2.578	4.017	3.637	2.317	0.977	Mann–Whitney U test
Event Angle (degrees)	92.868	5.243	92.271	91.551	6.361	93.362	0.601	Student's t-test

Supplementary Table S19. Comparing event counts and drop metrics of 7-day-old *Abeta42* males (n = 12) to 7-day-old *Abeta* females (n = 4). Pooled data from Tables S15-17. *p*-value for significance is $p < 0.05$.

	***Abeta* male**			***Abeta* female**				
**Metric**	**Mean**	**SD**	**Median**	**Mean**	**SD**	**Median**	***p* value**	**Test**
Event Counts								
Upward Jumps	0.083	0.276	0.000	0.500	0.500	0.500	0.089	Mann–Whitney U test
Arcjumps	0.083	0.276	0.000	0.000	0.000	0.000	0.665	Mann–Whitney U test
Falls	15.167	21.828	8.000	7.500	6.344	7.000	0.543	Mann–Whitney U test
Downward Jumps	2.000	3.894	0.000	0.750	0.829	0.500	1.000	Mann–Whitney U test
Drops	17.167	25.514	10.500	8.250	6.180	8.500	0.626	Mann–Whitney U test
Total Events	17.333	25.496	10.500	8.750	6.220	9.500	0.670	Mann–Whitney U test
Total Movement (mm)	6.50E+03	3.90E+03	7.06E+03	4.24E+03	2.49E+03	5.20E+03	0.327	Student's t-test
Time spent in upper half of vial	0.474	0.215	0.513	0.649	0.119	0.645	0.168	Student's t-test
Event-Movement ratio	2.31E-03	1.82E-03	1.81E-03	1.63E-03	1.23E-03	1.70E-03	0.770	Mann–Whitney U test
Drop Metrics								
Event duration (frames)	4.447	0.796	4.381	4.525	0.971	4.875	0.894	Student's t-test
Event duration (s)	0.077	0.014	0.076	0.078	0.017	0.084	0.894	Student's t-test
Vertical displacement (mm)	24.033	6.995	24.023	28.236	1.366	28.035	0.233	Mann–Whitney U test
Horizontal displacement (mm)	3.449	1.260	3.420	3.407	1.063	3.590	0.962	Student's t-test
Estimated height at event trigger (mm)	13.307	6.697	12.519	8.662	0.948	9.025	0.101	Mann–Whitney U test
Estimated height at event start (mm)	11.245	6.433	10.238	7.145	0.411	7.352	0.365	Mann–Whitney U test
Estimated X position at event start (mm)	19.870	4.068	20.732	16.756	3.271	17.344	0.273	Student's t-test
Estimated height at event end (mm)	35.279	2.244	35.360	35.381	1.743	35.387	0.947	Student's t-test
Estimated X position at event end (mm)	19.498	4.347	21.093	15.059	2.757	15.320	0.141	Student's t-test
Event velocity (mm/s)	370.249	99.108	368.318	489.437	193.965	363.034	0.189	Student's t-test
Maximum velocity (mm/s)	625.685	222.424	653.235	773.807	109.443	717.401	0.316	Student's t-test
Average velocity (mm/s)	344.594	89.001	342.373	502.199	190.845	367.422	0.180	Mann–Whitney U test
Initial vertical velocity (mm/s)	320.971	103.880	307.218	459.357	196.516	342.022	0.143	Student's t-test
Maximum acceleration (mm/s^2)	2.78E+04	1.17E+04	2.46E+04	3.64E+04	9.46E+03	3.04E+04	0.291	Student's t-test
Initial velocity (mm/s)	337.720	101.870	325.985	484.466	183.941	411.106	0.108	Student's t-test
Absolute initial velocity (mm/s)	337.720	101.870	325.985	484.466	183.941	411.106	0.108	Student's t-test
Maximum jerk (mm/s^3)	2.06E+06	1.28E+06	1.51E+06	2.18E+06	4.59E+05	2.32E+06	0.536	Mann–Whitney U test
Lag time (s)	4.017	3.637	2.317	1.218	0.162	1.108	0.018	Mann–Whitney U test
Event Angle (degrees)	91.551	6.361	93.362	92.768	0.827	92.649	0.763	Student's t-test

Supplementary Table S20. Comparing event counts of 7-day-old *w[1118]* males (n = 12) to 7-day-old *w[1118]* females (n = 4). Pooled data from Tables S15-17. *p*-value for significance is $p < 0.05$.

	***w[1118]* male**			***w[1118]* female**				
**Metric**	**Mean**	**SD**	**Median**	**Mean**	**SD**	**Median**	***p* value**	**Test**
Event Counts								
Upward Jumps	0.417	1.115	0.000	0.000	0.000	0.000	0.461	Mann–Whitney U test
Arcjumps	1.000	1.826	0.000	0.750	1.299	0.000	0.825	Mann–Whitney U test
Falls	10.750	6.808	12.500	13.750	11.691	9.500	0.568	Student's t-test
Downward Jumps	0.833	1.067	0.500	0.500	0.866	0.000	0.544	Mann–Whitney U test
Drops	11.583	7.555	13.000	14.250	12.517	9.500	0.641	Student's t-test
Total Events	13.000	9.806	13.500	15.000	13.766	9.500	0.771	Student's t-test
Total Movement (mm)	5.60E+03	1.38E+03	5.66E+03	8.47E+03	3.32E+03	6.83E+03	0.042	Mann–Whitney U test
Time spent in upper half of vial	0.547	0.064	0.560	0.666	0.154	0.652	0.277	Welch's t-test
Event-Movement ratio	2.38E-03	1.58E-03	2.36E-03	1.50E-03	8.56E-04	1.45E-03	0.340	Student's t-test
Drop Metrics								
Event duration (frames)	4.595	0.665	4.561	4.506	1.126	4.833	0.861	Student's t-test
Event duration (s)	0.079	0.011	0.079	0.078	0.019	0.083	0.861	Student's t-test
Vertical displacement (mm)	25.514	4.405	25.889	23.086	4.507	22.924	0.390	Student's t-test
Horizontal displacement (mm)	3.485	1.056	3.455	4.212	2.347	3.727	0.634	Welch's t-test
Estimated height at event trigger (mm)	11.119	3.762	10.848	12.232	3.757	11.415	0.639	Student's t-test
Estimated height at event start (mm)	9.544	3.566	8.871	10.979	4.103	9.662	0.541	Student's t-test
Estimated X position at event start (mm)	20.896	4.848	22.160	17.638	3.763	19.189	0.270	Student's t-test
Estimated height at event end (mm)	35.058	2.573	35.888	34.065	3.304	34.681	0.571	Student's t-test
Estimated X position at event end (mm)	19.978	5.290	21.632	16.819	2.492	16.379	0.299	Student's t-test
Event velocity (mm/s)	396.568	57.749	408.691	408.704	131.198	393.551	0.885	Welch's t-test
Maximum velocity (mm/s)	694.846	160.577	676.847	599.582	87.401	581.815	0.308	Student's t-test
Average velocity (mm/s)	410.729	100.472	399.518	364.465	98.825	334.120	0.466	Student's t-test
Initial vertical velocity (mm/s)	338.499	93.369	335.143	379.949	80.139	373.487	0.469	Student's t-test
Maximum acceleration (mm/s^2)	3.17E+04	1.11E+04	2.91E+04	2.64E+04	4.41E+03	2.59E+04	0.399	Student's t-test
Initial velocity (mm/s)	385.355	119.214	369.365	410.284	88.870	376.844	0.725	Student's t-test
Absolute initial velocity (mm/s)	385.355	119.214	369.365	410.284	88.870	376.844	0.725	Student's t-test
Maximum jerk (mm/s^3)	2.40E+06	1.08E+06	2.09E+06	1.55E+06	5.60E+05	1.54E+06	0.178	Student's t-test
Lag time (s)	3.370	2.357	2.578	1.658	0.298	1.670	0.042	Mann–Whitney U test
Event Angle (degrees)	92.868	5.243	92.271	90.341	9.322	92.206	0.539	Student's t-test

Supplementary Table S21. Comparing event counts and drop metrics of 15-day-old control females (n = 4) to 15-day-old *Abeta* females (n = 4). *p*-value for significance is $p < 0.05$.

	Control female			*Abeta* female				
**Metric**	**Mean**	**SD**	**Median**	**Mean**	**SD**	**Median**	***p* value**	**Test**
Event Counts								
Upward Jumps	0.250	0.433	0.000	0.000	0.000	0.000	0.453	Mann–Whitney U test
Arcjumps	0.000	0.000	0.000	0.000	0.000	0.000	-	Welch's t-test
Falls	24.500	10.012	20.500	6.000	2.236	6.000	0.020	Student's t-test
Downward Jumps	1.000	0.707	1.000	0.250	0.433	0.000	0.206	Mann–Whitney U test
Drops	25.500	10.642	21.500	6.250	1.920	6.000	0.022	Student's t-test
Total Events	25.750	10.473	22.000	6.250	1.920	6.000	0.019	Student's t-test
Total Movement (mm)	1.27E+04	1.65E+03	1.27E+04	5.65E+03	1.28E+03	5.66E+03	1.08E-03	Student's t-test
Time spent in upper half of vial	0.821	9.48E-03	0.821	0.737	0.101	0.761	0.242	Welch's t-test
Event-Movement ratio	2.02E-03	7.60E-04	1.79E-03	1.14E-03	3.47E-04	1.12E-03	0.117	Student's t-test
Drop Metrics								
Event duration (frames)	5.109	0.365	5.243	5.389	0.988	5.583	0.662	Student's t-test
Event duration (s)	0.088	6.30E-03	0.090	0.093	0.017	0.096	0.662	Student's t-test
Vertical displacement (mm)	30.370	1.394	30.305	26.597	2.871	27.047	0.087	Student's t-test
Horizontal displacement (mm)	3.675	1.157	3.891	4.940	1.683	5.054	0.325	Student's t-test
Estimated height at event trigger (mm)	8.867	1.086	8.639	13.055	3.648	11.696	0.105	Student's t-test
Estimated height at event start (mm)	8.515	0.953	8.409	12.097	3.593	10.893	0.146	Student's t-test
Estimated X position at event start (mm)	21.092	1.583	21.334	23.518	3.094	23.259	0.272	Student's t-test
Estimated height at event end (mm)	38.885	1.504	38.925	38.694	0.981	38.416	0.860	Student's t-test
Estimated X position at event end (mm)	20.552	1.104	20.673	21.010	1.557	20.947	0.692	Student's t-test
Event velocity (mm/s)	513.991	80.348	493.755	336.780	74.090	332.721	0.031	Student's t-test
Maximum velocity (mm/s)	681.639	121.380	697.718	725.078	54.547	710.893	0.592	Student's t-test
Average velocity (mm/s)	348.868	25.047	361.042	321.694	69.408	343.775	0.686	Mann–Whitney U test
Initial vertical velocity (mm/s)	307.677	34.451	290.375	260.458	15.892	257.559	0.114	Mann–Whitney U test
Maximum acceleration (mm/s^2)	2.96E+04	6.48E+03	2.99E+04	3.39E+04	5.63E+03	3.34E+04	0.417	Student's t-test
Initial velocity (mm/s)	313.543	33.685	297.607	284.769	19.091	291.983	0.245	Student's t-test
Absolute initial velocity (mm/s)	313.543	33.685	297.607	284.769	19.091	291.983	0.245	Student's t-test
Maximum jerk (mm/s^3)	2.43E+06	7.20E+05	2.46E+06	2.91E+06	8.15E+05	3.01E+06	0.477	Student's t-test
Lag time (s)	3.108	1.687	2.714	9.084	9.435	4.366	0.486	Mann–Whitney U test
Event Angle (degrees)	91.390	1.664	90.725	96.291	5.604	96.813	0.197	Student's t-test

Supplementary Table S22. Comparing event counts and drop metrics of 35-day-old control females (n = 4) to 35-day-old *Abeta* females (n = 4). *p*-value for significance is $p < 0.05$.

	Control female			*Abeta* female				
**Metric**	**Mean**	**SD**	**Median**	**Mean**	**SD**	**Median**	***p* value**	**Test**
Event Counts								
Upward Jumps	0.000	0.000	0.000	0.250	0.433	0.000	0.453	Mann–Whitney U test
Arcjumps	0.000	0.000	0.000	0.000	0.000	0.000	-	Welch's t-test
Falls	3.750	2.586	3.000	9.500	5.408	10.500	0.148	Student's t-test
Downward Jumps	0.000	0.000	0.000	0.750	0.829	0.500	0.215	Welch's t-test
Drops	3.750	2.586	3.000	10.250	5.214	11.500	0.101	Student's t-test
Total Events	3.750	2.586	3.000	10.500	5.220	12.000	0.092	Student's t-test
Total Movement (mm)	3.64E+03	2.20E+03	3.98E+03	4.00E+03	1.65E+03	4.56E+03	0.828	Student's t-test
Time spent in upper half of vial	0.614	0.055	0.624	0.607	0.127	0.631	0.940	Student's t-test
Event-Movement ratio	2.23E-03	2.59E-03	9.44E-04	2.44E-03	5.81E-04	2.55E-03	0.343	Mann–Whitney U test
Drop Metrics								
Event duration (frames)	3.906	2.113	4.000	3.692	1.744	4.227	0.896	Student's t-test
Event duration (s)	0.067	0.036	0.069	0.064	0.030	0.073	0.896	Student's t-test
Vertical displacement (mm)	17.074	9.812	19.026	23.361	6.754	23.242	0.343	Mann–Whitney U test
Horizontal displacement (mm)	6.580	4.439	5.127	3.647	1.986	3.213	0.337	Student's t-test
Estimated height at event trigger (mm)	12.050	2.861	12.610	11.286	4.721	9.013	0.886	Mann–Whitney U test
Estimated height at event start (mm)	11.230	2.517	11.391	10.706	4.233	8.763	0.686	Mann–Whitney U test
Estimated X position at event start (mm)	26.060	7.478	22.053	19.115	3.177	19.365	0.200	Mann–Whitney U test
Estimated height at event end (mm)	28.304	11.847	32.146	34.066	5.138	36.199	0.469	Student's t-test
Estimated X position at event end (mm)	23.318	1.841	23.724	17.021	3.189	15.917	0.025	Student's t-test
Event velocity (mm/s)	457.590	222.379	377.486	627.364	390.507	466.504	0.537	Student's t-test
Maximum velocity (mm/s)	707.351	94.402	722.129	822.072	300.503	784.565	0.551	Student's t-test
Average velocity (mm/s)	468.441	215.630	386.864	593.095	401.852	412.677	0.653	Student's t-test
Initial vertical velocity (mm/s)	203.080	58.247	191.161	499.046	277.372	388.571	0.120	Student's t-test
Maximum acceleration (mm/s^2)	3.31E+04	1.26E+04	3.50E+04	4.05E+04	2.05E+04	3.39E+04	0.618	Student's t-test
Initial velocity (mm/s)	418.409	247.265	351.410	587.483	408.930	402.013	0.562	Student's t-test
Absolute initial velocity (mm/s)	418.409	247.265	351.410	587.483	408.930	402.013	0.562	Student's t-test
Maximum jerk (mm/s^3)	1.40E+06	9.79E+05	1.60E+06	1.46E+06	8.95E+05	1.81E+06	0.946	Student's t-test
Lag time (s)	12.115	15.334	4.964	8.452	4.898	5.953	0.886	Mann–Whitney U test
Event Angle (degrees)	105.419	38.441	90.962	101.376	16.675	92.464	0.886	Mann–Whitney U test

Supplementary Table S23. Comparing event counts and drop metrics of 7-day-old *Abeta* females (n = 4) to 15-day-old *Abeta* females (n = 4). Pooled data from Tables S15 and S21. *p*-value for significance is $p < 0.05$.

	**7-day old *Abeta* female**			**15-day old *Abeta* female**				
**Metric**	**Mean**	**SD**	**Median**	**Mean**	**SD**	**Median**	***p* value**	**Test**
Event Counts								
Upward Jumps	0.500	0.500	0.500	0.000	0.000	0.000	0.181	Mann–Whitney U test
Arcjumps	0.000	0.000	0.000	0.000	0.000	0.000	-	Welch's t-test
Falls	7.500	6.344	7.000	6.000	2.236	6.000	0.720	Welch's t-test
Downward Jumps	0.750	0.829	0.500	0.250	0.433	0.000	0.505	Mann–Whitney U test
Drops	8.250	6.180	8.500	6.250	1.920	6.000	0.624	Welch's t-test
Total Events	8.750	6.220	9.500	6.250	1.920	6.000	0.546	Welch's t-test
Total Movement (mm)	4.24E+03	2.49E+03	5.20E+03	5.65E+03	1.28E+03	5.66E+03	0.416	Student's t-test
Time spent in upper half of vial	0.649	0.119	0.645	0.737	0.101	0.761	0.367	Student's t-test
Event-Movement ratio	1.63E-03	1.23E-03	1.70E-03	1.14E-03	3.47E-04	1.12E-03	0.551	Welch's t-test
Drop Metrics								
Event duration (frames)	4.525	0.971	4.875	5.389	0.988	5.583	0.375	Student's t-test
Event duration (s)	0.078	0.017	0.084	0.093	0.017	0.096	0.375	Student's t-test
Vertical displacement (mm)	28.236	1.366	28.035	26.597	2.871	27.047	0.475	Student's t-test
Horizontal displacement (mm)	3.407	1.063	3.590	4.940	1.683	5.054	0.295	Student's t-test
Estimated height at event trigger (mm)	8.662	0.948	9.025	13.055	3.648	11.696	0.146	Student's t-test
Estimated height at event start (mm)	7.145	0.411	7.352	12.097	3.593	10.893	0.101	Student's t-test
Estimated X position at event start (mm)	16.756	3.271	17.344	23.518	3.094	23.259	0.065	Student's t-test
Estimated height at event end (mm)	35.381	1.743	35.387	38.694	0.981	38.416	0.043	Student's t-test
Estimated X position at event end (mm)	15.059	2.757	15.320	21.010	1.557	20.947	0.028	Student's t-test
Event velocity (mm/s)	489.437	193.965	363.034	336.780	74.090	332.721	0.278	Student's t-test
Maximum velocity (mm/s)	773.807	109.443	717.401	725.078	54.547	710.893	0.543	Student's t-test
Average velocity (mm/s)	502.199	190.845	367.422	321.694	69.408	343.775	0.229	Mann–Whitney U test
Initial vertical velocity (mm/s)	459.357	196.516	342.022	260.458	15.892	257.559	0.149	Student's t-test
Maximum acceleration (mm/s^2)	3.64E+04	9.46E+03	3.04E+04	3.39E+04	5.63E+03	3.34E+04	0.722	Student's t-test
Initial velocity (mm/s)	484.466	183.941	411.106	284.769	19.091	291.983	0.128	Student's t-test
Absolute initial velocity (mm/s)	484.466	183.941	411.106	284.769	19.091	291.983	0.128	Student's t-test
Maximum jerk (mm/s^3)	2.18E+06	4.59E+05	2.32E+06	2.91E+06	8.15E+05	3.01E+06	0.293	Student's t-test
Lag time (s)	1.218	0.162	1.108	9.084	9.435	4.366	0.057	Mann–Whitney U test
Event Angle (degrees)	92.768	0.827	92.649	96.291	5.604	96.813	0.403	Student's t-test

Supplementary Table S24. Comparing event counts and drop metrics of 7-day-old *w[1118]* females (n = 4) to 15-day-old *w[1118]* females (n = 4). Pooled data from Tables S15 and S21. *p*-value for significance is $p < 0.05$.

	**7-day old *w[1118]* female**			**15-day old *w[1118]* female**				
**Metric**	**Mean**	**SD**	**Median**	**Mean**	**SD**	**Median**	***p* value**	**Test**
Event Counts								
Upward Jumps	0.000	0.000	0.000	0.250	0.433	0.000	0.453	Mann–Whitney U test
Arcjumps	0.750	1.299	0.000	0.000	0.000	0.000	0.453	Mann–Whitney U test
Falls	13.750	11.691	9.500	24.500	10.012	20.500	0.272	Student's t-test
Downward Jumps	0.500	0.866	0.000	1.000	0.707	1.000	0.436	Mann–Whitney U test
Drops	14.250	12.517	9.500	25.500	10.642	21.500	0.280	Student's t-test
Total Events	15.000	13.766	9.500	25.750	10.473	22.000	0.323	Student's t-test
Total Movement (mm)	8.47E+03	3.32E+03	6.83E+03	1.27E+04	1.65E+03	1.27E+04	0.200	Mann–Whitney U test
Time spent in upper half of vial	0.666	0.154	0.652	0.821	9.48E-03	0.821	0.179	Welch's t-test
Event-Movement ratio	1.50E-03	8.56E-04	1.45E-03	2.02E-03	7.60E-04	1.79E-03	0.462	Student's t-test
Drop Metrics								
Event duration (frames)	4.506	1.126	4.833	5.109	0.365	5.243	0.412	Student's t-test
Event duration (s)	0.078	0.019	0.083	0.088	6.30E-03	0.090	0.412	Student's t-test
Vertical displacement (mm)	23.086	4.507	22.924	30.370	1.394	30.305	0.063	Welch's t-test
Horizontal displacement (mm)	4.212	2.347	3.727	3.675	1.157	3.891	0.734	Student's t-test
Estimated height at event trigger (mm)	12.232	3.757	11.415	8.867	1.086	8.639	0.187	Student's t-test
Estimated height at event start (mm)	10.979	4.103	9.662	8.515	0.953	8.409	0.350	Student's t-test
Estimated X position at event start (mm)	17.638	3.763	19.189	21.092	1.583	21.334	0.193	Student's t-test
Estimated height at event end (mm)	34.065	3.304	34.681	38.885	1.504	38.925	0.061	Student's t-test
Estimated X position at event end (mm)	16.819	2.492	16.379	20.552	1.104	20.673	0.075	Welch's t-test
Event velocity (mm/s)	408.704	131.198	393.551	513.991	80.348	493.755	0.281	Student's t-test
Maximum velocity (mm/s)	599.582	87.401	581.815	681.639	121.380	697.718	0.379	Student's t-test
Average velocity (mm/s)	364.465	98.825	334.120	348.868	25.047	361.042	0.686	Mann–Whitney U test
Initial vertical velocity (mm/s)	379.949	80.139	373.487	307.677	34.451	290.375	0.486	Mann–Whitney U test
Maximum acceleration (mm/s^2)	2.64E+04	4.41E+03	2.59E+04	2.96E+04	6.48E+03	2.99E+04	0.506	Student's t-test
Initial velocity (mm/s)	410.284	88.870	376.844	313.543	33.685	297.607	0.128	Student's t-test
Absolute initial velocity (mm/s)	410.284	88.870	376.844	313.543	33.685	297.607	0.128	Student's t-test
Maximum jerk (mm/s^3)	1.55E+06	5.60E+05	1.54E+06	2.43E+06	7.20E+05	2.46E+06	0.145	Student's t-test
Lag time (s)	1.658	0.298	1.670	3.108	1.687	2.714	0.193	Student's t-test
Event Angle (degrees)	90.341	9.322	92.206	91.390	1.664	90.725	0.854	Student's t-test

Supplementary Table S25. Comparing event counts and drop metrics of 15-day-old *Abeta* females (n = 4) to 35-day-old *Abeta* females (n = 4). Pooled data from Tables S21-S22. *p*-value for significance is $p < 0.05$.

	**15-day old *Abeta* female**			**35-day old *Abeta* female**				
**Metric**	**Mean**	**SD**	**Median**	**Mean**	**SD**	**Median**	***p* value**	**Test**
Event Counts								
Upward Jumps	0.000	0.000	0.000	0.250	0.433	0.000	0.453	Mann–Whitney U test
Arcjumps	0.000	0.000	0.000	0.000	0.000	0.000	-	Welch's t-test
Falls	6.000	2.236	6.000	9.500	5.408	10.500	0.340	Student's t-test
Downward Jumps	0.250	0.433	0.000	0.750	0.829	0.500	0.505	Mann–Whitney U test
Drops	6.250	1.920	6.000	10.250	5.214	11.500	0.259	Student's t-test
Total Events	6.250	1.920	6.000	10.500	5.220	12.000	0.234	Student's t-test
Total Movement (mm)	5.65E+03	1.28E+03	5.66E+03	4.00E+03	1.65E+03	4.56E+03	0.220	Student's t-test
Time spent in upper half of vial	0.737	0.101	0.761	0.607	0.127	0.631	0.216	Student's t-test
Event-Movement ratio	1.14E-03	3.47E-04	1.12E-03	2.44E-03	5.81E-04	2.55E-03	0.016	Student's t-test
Drop Metrics								
Event duration (frames)	5.389	0.988	5.583	3.692	1.744	4.227	0.193	Student's t-test
Event duration (s)	0.093	0.017	0.096	0.064	0.030	0.073	0.193	Student's t-test
Vertical displacement (mm)	26.597	2.871	27.047	23.361	6.754	23.242	0.886	Mann–Whitney U test
Horizontal displacement (mm)	4.940	1.683	5.054	3.647	1.986	3.213	0.423	Student's t-test
Estimated height at event trigger (mm)	13.055	3.648	11.696	11.286	4.721	9.013	0.343	Mann–Whitney U test
Estimated height at event start (mm)	12.097	3.593	10.893	10.706	4.233	8.763	0.486	Mann–Whitney U test
Estimated X position at event start (mm)	23.518	3.094	23.259	19.115	3.177	19.365	0.136	Student's t-test
Estimated height at event end (mm)	38.694	0.981	38.416	34.066	5.138	36.199	0.176	Student's t-test
Estimated X position at event end (mm)	21.010	1.557	20.947	17.021	3.189	15.917	0.100	Student's t-test
Event velocity (mm/s)	336.780	74.090	332.721	627.364	390.507	466.504	0.252	Student's t-test
Maximum velocity (mm/s)	725.078	54.547	710.893	822.072	300.503	784.565	0.602	Student's t-test
Average velocity (mm/s)	321.694	69.408	343.775	593.095	401.852	412.677	0.293	Student's t-test
Initial vertical velocity (mm/s)	260.458	15.892	257.559	499.046	277.372	388.571	0.187	Student's t-test
Maximum acceleration (mm/s^2)	3.39E+04	5.63E+03	3.34E+04	4.05E+04	2.05E+04	3.39E+04	0.611	Student's t-test
Initial velocity (mm/s)	284.769	19.091	291.983	587.483	408.930	402.013	0.248	Student's t-test
Absolute initial velocity (mm/s)	284.769	19.091	291.983	587.483	408.930	402.013	0.248	Student's t-test
Maximum jerk (mm/s^3)	2.91E+06	8.15E+05	3.01E+06	1.46E+06	8.95E+05	1.81E+06	0.083	Student's t-test
Lag time (s)	9.084	9.435	4.366	8.452	4.898	5.953	0.686	Mann–Whitney U test
Event Angle (degrees)	96.291	5.604	96.813	101.376	16.675	92.464	0.886	Mann–Whitney U test

Supplementary Table S26. Comparing event counts and drop metrics of 15-day-old *w[1118]* females (n = 4) to 35-day-old *w[1118]* females (n = 4). Pooled data from Tables S21-S22. *p*-value for significance is $p < 0.05$.

	**15-day old *w[1118]* female**			**35-day old *w[1118]* female**				
**Metric**	**Mean**	**SD**	**Median**	**Mean**	**SD**	**Median**	***p* value**	**Test**
Event Counts								
Upward Jumps	0.250	0.433	0.000	0.000	0.000	0.000	0.453	Mann–Whitney U test
Arcjumps	0.000	0.000	0.000	0.000	0.000	0.000	-	Welch's t-test
Falls	24.500	10.012	20.500	3.750	2.586	3.000	0.013	Student's t-test
Downward Jumps	1.000	0.707	1.000	0.000	0.000	0.000	0.048	Student's t-test
Drops	25.500	10.642	21.500	3.750	2.586	3.000	0.014	Student's t-test
Total Events	25.750	10.473	22.000	3.750	2.586	3.000	0.012	Student's t-test
Total Movement (mm)	1.27E+04	1.65E+03	1.27E+04	3.64E+03	2.20E+03	3.98E+03	1.22E-03	Student's t-test
Time spent in upper half of vial	0.821	9.48E-03	0.821	0.614	0.055	0.624	6.56E-03	Welch's t-test
Event-Movement ratio	2.02E-03	7.60E-04	1.79E-03	2.23E-03	2.59E-03	9.44E-04	0.486	Mann–Whitney U test
Drop Metrics								
Event duration (frames)	5.389	0.988	5.583	3.692	1.744	4.227	0.193	Student's t-test
Event duration (s)	0.093	0.017	0.096	0.064	0.030	0.073	0.193	Student's t-test
Vertical displacement (mm)	26.597	2.871	27.047	23.361	6.754	23.242	0.886	Mann–Whitney U test
Horizontal displacement (mm)	4.940	1.683	5.054	3.647	1.986	3.213	0.423	Student's t-test
Estimated height at event trigger (mm)	13.055	3.648	11.696	11.286	4.721	9.013	0.343	Mann–Whitney U test
Estimated height at event start (mm)	12.097	3.593	10.893	10.706	4.233	8.763	0.486	Mann–Whitney U test
Estimated X position at event start (mm)	23.518	3.094	23.259	19.115	3.177	19.365	0.136	Student's t-test
Estimated height at event end (mm)	38.694	0.981	38.416	34.066	5.138	36.199	0.176	Student's t-test
Estimated X position at event end (mm)	21.010	1.557	20.947	17.021	3.189	15.917	0.100	Student's t-test
Event velocity (mm/s)	336.780	74.090	332.721	627.364	390.507	466.504	0.252	Student's t-test
Maximum velocity (mm/s)	725.078	54.547	710.893	822.072	300.503	784.565	0.602	Student's t-test
Average velocity (mm/s)	321.694	69.408	343.775	593.095	401.852	412.677	0.293	Student's t-test
Initial vertical velocity (mm/s)	260.458	15.892	257.559	499.046	277.372	388.571	0.187	Student's t-test
Maximum acceleration (mm/s^2)	3.39E+04	5.63E+03	3.34E+04	4.05E+04	2.05E+04	3.39E+04	0.611	Student's t-test
Initial velocity (mm/s)	284.769	19.091	291.983	587.483	408.930	402.013	0.248	Student's t-test
Absolute initial velocity (mm/s)	284.769	19.091	291.983	587.483	408.930	402.013	0.248	Student's t-test
Maximum jerk (mm/s^3)	2.91E+06	8.15E+05	3.01E+06	1.46E+06	8.95E+05	1.81E+06	0.083	Student's t-test
Lag time (s)	9.084	9.435	4.366	8.452	4.898	5.953	0.686	Mann–Whitney U test
Event Angle (degrees)	96.291	5.604	96.813	101.376	16.675	92.464	0.886	Mann–Whitney U test

Supplementary Table S27. Comparing event counts and drop metrics of 15-day-old *Abeta* females (n = 4) to 35-day-old *Abeta* females (n = 4). Pooled data from Tables S21-S22. *p*-value for significance is $p < 0.05$.

	**7-day old *Abeta* female**			**35-day old *Abeta* female**				
**Metric**	**Mean**	**SD**	**Median**	**Mean**	**SD**	**Median**	***p* value**	**Test**
Event Counts								
Upward Jumps	0.500	0.500	0.500	0.250	0.433	0.000	0.608	Mann–Whitney U test
Arcjumps	0.000	0.000	0.000	0.000	0.000	0.000	-	Welch's t-test
Falls	7.500	6.344	7.000	9.500	5.408	10.500	0.692	Student's t-test
Downward Jumps	0.750	0.829	0.500	0.750	0.829	0.500	1.000	Student's t-test
Drops	8.250	6.180	8.500	10.250	5.214	11.500	0.683	Student's t-test
Total Events	8.750	6.220	9.500	10.500	5.220	12.000	0.722	Student's t-test
Total Movement (mm)	4.24E+03	2.49E+03	5.20E+03	4.00E+03	1.65E+03	4.56E+03	0.893	Student's t-test
Time spent in upper half of vial	0.649	0.119	0.645	0.607	0.127	0.631	0.693	Student's t-test
Event-Movement ratio	1.63E-03	1.23E-03	1.70E-03	2.44E-03	5.81E-04	2.55E-03	0.339	Student's t-test
Drop Metrics								
Event duration (frames)	4.525	0.971	4.875	3.692	1.744	4.227	0.556	Student's t-test
Event duration (s)	0.078	0.017	0.084	0.064	0.030	0.073	0.556	Student's t-test
Vertical displacement (mm)	28.236	1.366	28.035	23.361	6.754	23.242	0.857	Mann–Whitney U test
Horizontal displacement (mm)	3.407	1.063	3.590	3.647	1.986	3.213	0.879	Student's t-test
Estimated height at event trigger (mm)	8.662	0.948	9.025	11.286	4.721	9.013	0.857	Mann–Whitney U test
Estimated height at event start (mm)	7.145	0.411	7.352	10.706	4.233	8.763	0.229	Mann–Whitney U test
Estimated X position at event start (mm)	16.756	3.271	17.344	19.115	3.177	19.365	0.454	Student's t-test
Estimated height at event end (mm)	35.381	1.743	35.387	34.066	5.138	36.199	0.734	Student's t-test
Estimated X position at event end (mm)	15.059	2.757	15.320	17.021	3.189	15.917	0.503	Student's t-test
Event velocity (mm/s)	489.437	193.965	363.034	627.364	390.507	466.504	0.655	Student's t-test
Maximum velocity (mm/s)	773.807	109.443	717.401	822.072	300.503	784.565	0.831	Student's t-test
Average velocity (mm/s)	502.199	190.845	367.422	593.095	401.852	412.677	0.857	Mann–Whitney U test
Initial vertical velocity (mm/s)	459.357	196.516	342.022	499.046	277.372	388.571	0.865	Student's t-test
Maximum acceleration (mm/s^2)	3.64E+04	9.46E+03	3.04E+04	4.05E+04	2.05E+04	3.39E+04	0.800	Student's t-test
Initial velocity (mm/s)	484.466	183.941	411.106	587.483	408.930	402.013	0.745	Student's t-test
Absolute initial velocity (mm/s)	484.466	183.941	411.106	587.483	408.930	402.013	0.745	Student's t-test
Maximum jerk (mm/s^3)	2.18E+06	4.59E+05	2.32E+06	1.46E+06	8.95E+05	1.81E+06	0.330	Student's t-test
Lag time (s)	1.218	0.162	1.108	8.452	4.898	5.953	0.057	Mann–Whitney U test
Event Angle (degrees)	92.768	0.827	92.649	101.376	16.675	92.464	0.857	Mann–Whitney U test

Supplementary Table S28. Comparing event counts and drop metrics of 7-day-old *w[1118]* females (n = 4) to 35-day-old *w[1118]* females (n = 4). Pooled data from Tables S15 and S22. *p*-value for significance is $p < 0.05$.

	**7-day old *w[1118]* female**			**35-day old *w[1118]* female**				
**Metric**	**Mean**	**SD**	**Median**	**Mean**	**SD**	**Median**	***p* value**	**Test**
Event Counts								
Upward Jumps	0.000	0.000	0.000	0.000	0.000	0.000	-	Welch's t-test
Arcjumps	0.750	1.299	0.000	0.000	0.000	0.000	0.453	Mann–Whitney U test
Falls	13.750	11.691	9.500	3.750	2.586	3.000	0.198	Student's t-test
Downward Jumps	0.500	0.866	0.000	0.000	0.000	0.000	0.453	Mann–Whitney U test
Drops	14.250	12.517	9.500	3.750	2.586	3.000	0.205	Student's t-test
Total Events	15.000	13.766	9.500	3.750	2.586	3.000	0.214	Student's t-test
Total Movement (mm)	8.47E+03	3.32E+03	6.83E+03	3.64E+03	2.20E+03	3.98E+03	0.057	Mann–Whitney U test
Time spent in upper half of vial	0.666	0.154	0.652	0.614	0.055	0.624	0.603	Student's t-test
Event-Movement ratio	1.50E-03	8.56E-04	1.45E-03	2.23E-03	2.59E-03	9.44E-04	0.886	Mann–Whitney U test
Drop Metrics								
Event duration (frames)	4.506	1.126	4.833	3.906	2.113	4.000	0.679	Student's t-test
Event duration (s)	0.078	0.019	0.083	0.067	0.036	0.069	0.679	Student's t-test
Vertical displacement (mm)	23.086	4.507	22.924	17.074	9.812	19.026	0.372	Student's t-test
Horizontal displacement (mm)	4.212	2.347	3.727	6.580	4.439	5.127	0.445	Student's t-test
Estimated height at event trigger (mm)	12.232	3.757	11.415	12.050	2.861	12.610	0.949	Student's t-test
Estimated height at event start (mm)	10.979	4.103	9.662	11.230	2.517	11.391	0.931	Student's t-test
Estimated X position at event start (mm)	17.638	3.763	19.189	26.060	7.478	22.053	0.029	Mann–Whitney U test
Estimated height at event end (mm)	34.065	3.304	34.681	28.304	11.847	32.146	0.448	Student's t-test
Estimated X position at event end (mm)	16.819	2.492	16.379	23.318	1.841	23.724	0.011	Student's t-test
Event velocity (mm/s)	408.704	131.198	393.551	457.590	222.379	377.486	0.754	Student's t-test
Maximum velocity (mm/s)	599.582	87.401	581.815	707.351	94.402	722.129	0.197	Student's t-test
Average velocity (mm/s)	364.465	98.825	334.120	468.441	215.630	386.864	0.476	Student's t-test
Initial vertical velocity (mm/s)	379.949	80.139	373.487	203.080	58.247	191.161	0.021	Student's t-test
Maximum acceleration (mm/s^2)	2.64E+04	4.41E+03	2.59E+04	3.31E+04	1.26E+04	3.50E+04	0.433	Welch's t-test
Initial velocity (mm/s)	410.284	88.870	376.844	418.409	247.265	351.410	0.959	Student's t-test
Absolute initial velocity (mm/s)	410.284	88.870	376.844	418.409	247.265	351.410	0.959	Student's t-test
Maximum jerk (mm/s^3)	1.55E+06	5.60E+05	1.54E+06	1.40E+06	9.79E+05	1.60E+06	0.829	Student's t-test
Lag time (s)	1.658	0.298	1.670	12.115	15.334	4.964	0.282	Student's t-test
Event Angle (degrees)	90.341	9.322	92.206	105.419	38.441	90.962	0.534	Student's t-test

Supplementary Table S29. Comparing event counts and drop metrics of 2-week-old control males (n = 8) to 2-week-old *SNCA* males (n = 8), Cohort 1. *p*-value for significance is $p < 0.05$.

	**Control male**			***SNCA* male**				
**Metric**	**Mean**	**SD**	**Median**	**Mean**	**SD**	**Median**	***p* value**	**Test**
Event Counts								
Upward Jumps	0	0	0	0	0	0	-	Welch's t-test
Arcjumps	0	0	0	0.125	0.331	0	0.382	Mann–Whitney U test
Falls	11	3.279	11	14.875	9.373	12	0.33	Welch's t-test
Downward Jumps	1.5	1.581	1	0.875	0.781	1	0.616	Mann–Whitney U test
Drops	12.5	4	12.5	15.75	9.203	13	0.406	Student's t-test
Total Events	12.5	4	12.5	15.875	9.266	13	0.391	Student's t-test
Total Movement (mm)	3.79E+03	2.18E+03	3.54E+03	4.90E+03	2.55E+03	4.22E+03	0.399	Student's t-test
Time spent in upper half of vial	0.598	0.109	0.561	0.469	0.161	0.393	0.102	Student's t-test
Event-Movement ratio	3.88E-03	1.44E-03	3.88E-03	3.43E-03	1.73E-03	3.06E-03	0.442	Mann–Whitney U test
Drop Metrics								
Event duration (frames)	4.213	0.605	4.333	4.575	0.745	4.637	0.336	Student's t-test
Event duration (s)	0.073	0.01	0.075	0.079	0.013	0.08	0.336	Student's t-test
Vertical displacement (mm)	25.399	5.049	25.268	23.472	5.132	22.831	0.49	Student's t-test
Horizontal displacement (mm)	3.673	1.291	3.053	3.73	1.13	3.737	0.931	Student's t-test
Estimated height at event trigger (mm)	11.576	4.4	10.625	12.868	3.837	14.467	0.567	Student's t-test
Estimated height at event start (mm)	11.011	4.027	10.324	12.272	3.612	13.437	0.547	Student's t-test
Estimated X position at event start (mm)	21.65	2.34	21.932	21.759	2.289	21.093	0.931	Student's t-test
Estimated height at event end (mm)	36.41	3.702	36.93	35.744	3.363	35.797	0.73	Student's t-test
Estimated X position at event end (mm)	20.578	1.578	21.143	20.736	2.109	20.222	0.877	Student's t-test
Event velocity (mm/s)	581.365	231.565	500.177	493.629	81.988	481.92	0.361	Student's t-test
Maximum velocity (mm/s)	716.127	251.619	647.258	588.747	112.539	594.148	0.242	Student's t-test
Average velocity (mm/s)	416.54	173.48	370.596	324.376	53.578	304.012	0.201	Student's t-test
Initial vertical velocity (mm/s)	381.541	145.918	330.011	324.251	93.093	298.06	0.505	Mann–Whitney U test
Maximum acceleration (mm/s^2)	3.50E+04	1.48E+04	3.31E+04	2.69E+04	8.06E+03	2.42E+04	0.224	Student's t-test
Initial velocity (mm/s)	398.077	152.711	341.778	336.101	92.148	303.811	0.442	Mann–Whitney U test
Absolute initial velocity (mm/s)	398.077	152.711	341.778	336.101	92.148	303.811	0.442	Mann–Whitney U test
Maximum jerk (mm/s^3)	2.21E+06	1.56E+06	2.17E+06	1.47E+06	4.60E+05	1.30E+06	0.263	Welch's t-test
Lag time (s)	4.002	2.011	4.147	8.583	8.255	5.373	0.176	Student's t-test
Event Angle (degrees)	91.943	4.192	91.345	91.793	4.277	93.42	0.948	Student's t-test

Supplementary Table S30. Comparing event counts and drop metrics of 2-week-old control males (n = 8) to 2-week-old *SNCA* males (n = 8), Cohort 2. *p*-value for significance is $p < 0.05$.

	**Control male**			***SNCA* male**				
**Metric**	**Mean**	**SD**	**Median**	**Mean**	**SD**	**Median**	***p* value**	**Test**
Event Counts								
Upward Jumps	0.125	0.331	0	0.125	0.331	0	1	Mann–Whitney U test
Arcjumps	0	0	0	0.125	0.331	0	0.382	Mann–Whitney U test
Falls	14.75	9.08	12.5	19.25	8.408	21	0.225	Mann–Whitney U test
Downward Jumps	0.75	0.829	0.5	0.625	1.111	0	0.585	Mann–Whitney U test
Drops	15.5	9.631	13.5	19.875	8.838	22	0.368	Mann–Whitney U test
Total Events	15.625	9.604	14	20.125	8.652	22	0.22	Mann–Whitney U test
Total Movement (mm)	4.22E+03	1.85E+03	4.55E+03	5.19E+03	2.64E+03	5.74E+03	0.44	Student's t-test
Time spent in upper half of vial	0.517	0.138	0.516	0.417	0.119	0.391	0.166	Student's t-test
Event-Movement ratio	3.96E-03	1.93E-03	2.84E-03	4.31E-03	1.79E-03	4.11E-03	0.721	Mann–Whitney U test
Drop Metrics								
Event duration (frames)	4.878	0.694	4.821	5.06	0.604	5.136	0.611	Student's t-test
Event duration (s)	0.084	0.012	0.083	0.087	0.01	0.089	0.611	Student's t-test
Vertical displacement (mm)	25.866	3.036	26.19	25.335	3.348	25.552	0.761	Student's t-test
Horizontal displacement (mm)	4.064	1.025	4.34	4.4	1.98	3.717	0.696	Student's t-test
Estimated height at event trigger (mm)	11.585	2.334	11.764	13.503	2.064	13.378	0.126	Student's t-test
Estimated height at event start (mm)	11.017	2.306	11.319	12.35	2.045	12.29	0.272	Student's t-test
Estimated X position at event start (mm)	20.498	1.853	20.874	21.829	2.721	21.258	0.303	Student's t-test
Estimated height at event end (mm)	36.883	2.306	36.854	37.686	2.976	38.382	0.582	Student's t-test
Estimated X position at event end (mm)	20.016	1.022	19.948	20.008	1.478	20.332	0.991	Student's t-test
Event velocity (mm/s)	418.054	92.497	428.47	404.119	94.827	405.728	0.785	Student's t-test
Maximum velocity (mm/s)	670.75	154.993	671.468	567.98	98.328	563.249	0.161	Student's t-test
Average velocity (mm/s)	326.715	26.141	320.289	299.408	36.519	304.885	0.13	Student's t-test
Initial vertical velocity (mm/s)	293.167	36.476	290.197	255.129	57.952	234.831	0.164	Student's t-test
Maximum acceleration (mm/s^2)	3.09E+04	1.03E+04	3.06E+04	2.40E+04	5.93E+03	2.28E+04	0.142	Student's t-test
Initial velocity (mm/s)	300.615	37.239	297.337	265.19	58.434	242.84	0.13	Mann–Whitney U test
Absolute initial velocity (mm/s)	300.615	37.239	297.337	265.19	58.434	242.84	0.13	Mann–Whitney U test
Maximum jerk (mm/s^3)	2.51E+06	1.36E+06	2.42E+06	1.82E+06	5.74E+05	1.76E+06	0.237	Student's t-test
Lag time (s)	4.51	2.036	4.475	3.02	2.376	2.02	0.083	Mann–Whitney U test
Event Angle (degrees)	91.347	3.678	91.522	93.678	6.404	90.838	0.798	Mann–Whitney U test

Supplementary Table S31. Comparing event counts and drop metrics of 2-week-old control males (n = 8) to 2-week-old *SNCA* males (n = 8), Cohort 3. *p*-value for significance is $p < 0.05$.

	Control male			*SNCA* male				
**Metric**	**Mean**	**SD**	**Median**	**Mean**	**SD**	**Median**	***p* value**	**Test**
Event Counts								
Upward Jumps	0.125	0.331	0	0	0	0	0.382	Mann–Whitney U test
Arcjumps	0.25	0.433	0	0.125	0.331	0	0.587	Mann–Whitney U test
Falls	12.75	7.207	11	13.875	10.729	10	0.821	Student's t-test
Downward Jumps	0.5	1	0	0.375	0.484	0	0.796	Mann–Whitney U test
Drops	13.25	7.902	11.5	14.25	10.894	10	0.847	Student's t-test
Total Events	13.625	7.549	11.5	14.375	11.101	10	0.885	Student's t-test
Total Movement (mm)	3.62E+03	1.86E+03	3.63E+03	4.88E+03	1.82E+03	5.33E+03	0.219	Student's t-test
Time spent in upper half of vial	0.449	0.104	0.423	0.46	0.097	0.461	0.843	Student's t-test
Event-Movement ratio	3.99E-03	1.32E-03	4.26E-03	2.83E-03	1.72E-03	2.52E-03	0.178	Student's t-test
Drop Metrics								
Event duration (frames)	4.789	0.794	4.889	4.509	0.673	4.515	0.488	Student's t-test
Event duration (s)	0.083	0.014	0.084	0.078	0.012	0.078	0.488	Student's t-test
Vertical displacement (mm)	24.86	3.984	23.93	24.843	1.703	25.16	0.992	Student's t-test
Horizontal displacement (mm)	4.242	1.439	4.569	3.79	1.129	3.562	0.524	Student's t-test
Estimated height at event trigger (mm)	14.18	4.798	12.785	11.142	3.094	10.773	0.181	Student's t-test
Estimated height at event start (mm)	13.135	4.3	11.933	10.446	2.964	9.909	0.195	Student's t-test
Estimated X position at event start (mm)	19.823	2.189	19.841	20.631	2.123	20.338	0.495	Student's t-test
Estimated height at event end (mm)	37.995	2.896	38.605	35.29	2.436	36.216	0.079	Student's t-test
Estimated X position at event end (mm)	20.144	1.294	20.02	19.939	2.384	20.228	0.844	Student's t-test
Event velocity (mm/s)	508.668	103.842	495.865	495.911	168.518	453.337	0.867	Student's t-test
Maximum velocity (mm/s)	498.926	153.771	486.025	516.033	106.237	543.095	0.721	Mann–Whitney U test
Average velocity (mm/s)	269.124	42.268	270.181	316.647	101.102	312.196	0.27	Student's t-test
Initial vertical velocity (mm/s)	238.371	38.968	243.867	265.501	94.889	243.823	0.878	Mann–Whitney U test
Maximum acceleration (mm/s^2)	2.21E+04	7.16E+03	2.12E+04	2.19E+04	6.37E+03	2.17E+04	0.946	Student's t-test
Initial velocity (mm/s)	247.573	43.357	251.629	278.228	106.187	252.596	0.878	Mann–Whitney U test
Absolute initial velocity (mm/s)	247.573	43.357	251.629	278.228	106.187	252.596	0.878	Mann–Whitney U test
Maximum jerk (mm/s^3)	1.38E+06	8.66E+05	1.25E+06	1.26E+06	6.87E+05	1.28E+06	0.78	Student's t-test
Lag time (s)	5.672	6.447	3.492	6.067	3.976	5.272	0.234	Mann–Whitney U test
Event Angle (degrees)	89.552	5.676	90.591	91.508	4.719	90.671	0.495	Student's t-test

Supplementary Table S32. Comparing event counts and drop metrics of 3-week-old control males (n = 4) to 3-week-old *SNCA* males (n = 8), Cohort 1. *p*-value for significance is $p < 0.05$.

	**Control male**			***SNCA* male**				
**Metric**	**Mean**	**SD**	**Median**	**Mean**	**SD**	**Median**	***p* value**	**Test**
Event Counts								
Upward Jumps	0	0	0	0.125	0.331	0	0.596	Mann–Whitney U test
Arcjumps	0.25	0.433	0	0	0	0	0.216	Mann–Whitney U test
Falls	4.25	4.603	3	5	4	3.5	0.796	Student's t-test
Downward Jumps	0.5	0.5	0.5	0.125	0.331	0	0.215	Mann–Whitney U test
Drops	4.75	5.068	3.5	5.125	4.166	3.5	0.67	Mann–Whitney U test
Total Events	5	4.848	4	5.25	4.409	3.5	0.733	Mann–Whitney U test
Total Movement (mm)	1.34E+03	917.296	1.38E+03	2.50E+03	1.61E+03	1.86E+03	0.247	Student's t-test
Time spent in upper half of vial	0.342	0.164	0.359	0.402	0.186	0.419	0.631	Student's t-test
Event-Movement ratio	2.89E-03	1.89E-03	3.13E-03	2.29E-03	1.14E-03	2.36E-03	0.545	Student's t-test
Drop Metrics								
Event duration (frames)	3.935	0.649	3.935	5.041	0.882	4.962	0.239	Mann–Whitney U test
Event duration (s)	0.068	0.011	0.068	0.087	0.015	0.086	0.239	Mann–Whitney U test
Vertical displacement (mm)	22.693	3.229	22.693	25.722	3.962	27.153	0.267	Mann–Whitney U test
Horizontal displacement (mm)	2.281	0.833	2.281	2.456	1.029	2.327	0.711	Mann–Whitney U test
Estimated height at event trigger (mm)	17.782	5.088	17.782	12.353	5.593	10.624	0.4	Mann–Whitney U test
Estimated height at event start (mm)	16.574	4.297	16.574	11.672	5.038	10.003	0.4	Mann–Whitney U test
Estimated X position at event start (mm)	21.573	0.772	21.573	20.66	2.775	20.742	0.533	Mann–Whitney U test
Estimated height at event end (mm)	39.268	1.068	39.268	37.393	4.086	37.35	0.533	Mann–Whitney U test
Estimated X position at event end (mm)	20.33	0.88	20.33	20.054	1.249	20.07	0.711	Mann–Whitney U test
Event velocity (mm/s)	404.246	20.408	404.246	376.318	129.029	343.105	0.889	Mann–Whitney U test
Maximum velocity (mm/s)	645.648	51.336	645.648	629.979	121.465	660.228	0.889	Mann–Whitney U test
Average velocity (mm/s)	322.523	0.58	322.523	312.209	70.277	307.082	0.533	Mann–Whitney U test
Initial vertical velocity (mm/s)	327.119	19.444	327.119	216.352	70.212	218.522	0.178	Mann–Whitney U test
Maximum acceleration (mm/s^2)	2.88E+04	1.99E+03	2.88E+04	2.59E+04	5.72E+03	2.77E+04	0.889	Mann–Whitney U test
Initial velocity (mm/s)	334.079	16.107	334.079	225.591	68.383	221.322	0.178	Mann–Whitney U test
Absolute initial velocity (mm/s)	334.079	16.107	334.079	225.591	68.383	221.322	0.178	Mann–Whitney U test
Maximum jerk (mm/s^3)	2.33E+06	3.17E+05	2.33E+06	1.93E+06	9.58E+05	2.51E+06	1	Mann–Whitney U test
Lag time (s)	6.742	2.391	6.742	6.64	4.004	5.961	0.889	Mann–Whitney U test
Event Angle (degrees)	94.083	0.47	94.083	91.875	3.684	91.485	0.4	Mann–Whitney U test

Supplementary Table S33. Comparing event counts and drop metrics of 3-week-old control males (n = 8) to 3-week-old *SNCA* males (n = 8), Cohort 2. *p*-value for significance is $p < 0.05$.

	**Control male**			***SNCA* male**				
**Metric**	**Mean**	**SD**	**Median**	**Mean**	**SD**	**Median**	***p* value**	**Test**
Event Counts								
Upward Jumps	0	0	0	0	0	0	-	Welch's t-test
Arcjumps	0	0	0	0	0	0	-	Welch's t-test
Falls	5.125	2.619	6.5	4.875	3.333	4.5	0.595	Mann–Whitney U test
Downward Jumps	0.25	0.433	0	0.25	0.433	0	1	Mann–Whitney U test
Drops	5.375	2.87	6.5	5.125	3.14	5	0.879	Student's t-test
Total Events	5.375	2.87	6.5	5.125	3.14	5	0.879	Student's t-test
Total Movement (mm)	1.46E+03	895.858	1.49E+03	1.77E+03	1.23E+03	1.34E+03	0.798	Mann–Whitney U test
Time spent in upper half of vial	0.457	0.209	0.427	0.447	0.083	0.444	0.906	Student's t-test
Event-Movement ratio	4.17E-03	1.90E-03	3.45E-03	2.91E-03	7.86E-04	3.03E-03	0.126	Student's t-test
Drop Metrics								
Event duration (frames)	4.639	0.745	4.802	4.496	0.851	4.65	0.744	Student's t-test
Event duration (s)	0.08	0.013	0.083	0.078	0.015	0.08	0.744	Student's t-test
Vertical displacement (mm)	26.21	5.536	27.672	21.779	5	23.329	0.138	Student's t-test
Horizontal displacement (mm)	3.281	1.58	3.26	3.239	1.514	2.98	0.96	Student's t-test
Estimated height at event trigger (mm)	14.658	6.423	12.509	17.938	6.892	18.165	0.373	Student's t-test
Estimated height at event start (mm)	13.038	5.394	11.453	16.599	6.464	16.14	0.282	Student's t-test
Estimated X position at event start (mm)	20.338	2.664	20.941	19.767	2.406	20.223	0.681	Student's t-test
Estimated height at event end (mm)	39.248	1.435	39.125	38.378	3.223	38.921	0.525	Student's t-test
Estimated X position at event end (mm)	19.462	1.923	18.818	19.151	2.221	19.904	0.574	Mann–Whitney U test
Event velocity (mm/s)	501.382	226.57	410.625	341.23	101.021	298.141	0.05	Mann–Whitney U test
Maximum velocity (mm/s)	615.914	205.309	673.068	708.041	253.303	625.77	0.467	Student's t-test
Average velocity (mm/s)	320.234	79.254	337.362	340.554	108.083	313.722	1	Mann–Whitney U test
Initial vertical velocity (mm/s)	260.121	46.006	251.711	282.519	87.197	284.282	0.56	Welch's t-test
Maximum acceleration (mm/s^2)	2.92E+04	1.06E+04	2.87E+04	3.35E+04	1.61E+04	2.64E+04	0.959	Mann–Whitney U test
Initial velocity (mm/s)	270.084	47.588	271.168	357.177	212.025	292.233	0.645	Mann–Whitney U test
Absolute initial velocity (mm/s)	270.084	47.588	271.168	357.177	212.025	292.233	0.645	Mann–Whitney U test
Maximum jerk (mm/s^3)	2.08E+06	1.29E+06	2.44E+06	2.83E+06	1.78E+06	2.15E+06	0.798	Mann–Whitney U test
Lag time (s)	11.905	7.582	12.523	10.133	13.913	4.945	0.152	Mann–Whitney U test
Event Angle (degrees)	91.377	6.065	90.468	92.738	4.842	90.959	0.645	Mann–Whitney U test

Supplementary Table S34. Comparing event counts and drop metrics of 3-week-old control males (n = 8) to 3-week-old *SNCA* males (n = 8), Cohort 3. *p*-value for significance is $p < 0.05$.

	**Control male**			***SNCA* male**				
**Metric**	**Mean**	**SD**	**Median**	**Mean**	**SD**	**Median**	***p* value**	**Test**
Event Counts								
Upward Jumps	0.375	0.484	0	0.125	0.331	0	0.295	Mann–Whitney U test
Arcjumps	0.5	0.866	0	0.375	0.992	0	0.7	Mann–Whitney U test
Falls	7.625	5.195	8	6	2.062	6	0.461	Welch's t-test
Downward Jumps	0.5	0.707	0	1.5	1.5	1	0.131	Mann–Whitney U test
Drops	8.125	5.061	9.5	7.5	2.121	7.5	0.77	Welch's t-test
Total Events	9	5.701	11	8	2.915	7.5	0.686	Student's t-test
Total Movement (mm)	2.97E+03	1.42E+03	3.43E+03	3.23E+03	1.78E+03	2.37E+03	0.798	Mann–Whitney U test
Time spent in upper half of vial	0.332	0.107	0.348	0.473	0.148	0.459	0.061	Student's t-test
Event-Movement ratio	2.68E-03	1.06E-03	2.84E-03	2.68E-03	6.13E-04	2.63E-03	0.993	Student's t-test
Drop Metrics								
Event duration (frames)	3.895	1.664	4.472	4.106	0.782	4.619	0.713	Mann–Whitney U test
Event duration (s)	0.067	0.029	0.077	0.071	0.013	0.08	0.721	Mann–Whitney U test
Vertical displacement (mm)	23.661	7.312	25.6	26.8	3.786	28.152	0.33	Student's t-test
Horizontal displacement (mm)	3.022	1.969	2.546	3.178	1.227	3.204	0.862	Student's t-test
Estimated height at event trigger (mm)	16.848	7.544	14.737	12.22	3.947	11.277	0.172	Student's t-test
Estimated height at event start (mm)	15.906	7.108	13.936	11.677	3.475	11.063	0.179	Student's t-test
Estimated X position at event start (mm)	19.284	2.68	19.683	20.826	1.658	21.512	0.216	Student's t-test
Estimated height at event end (mm)	39.568	2.355	39.714	38.477	1.273	38.563	0.299	Student's t-test
Estimated X position at event end (mm)	19.528	2.143	19.087	20.842	1.397	20.943	0.196	Student's t-test
Event velocity (mm/s)	581.86	528.832	394.445	589.546	179.847	583.601	0.161	Mann–Whitney U test
Maximum velocity (mm/s)	865.741	470.823	708.918	881.922	345.1	782.373	0.943	Student's t-test
Average velocity (mm/s)	575.73	530.143	368.037	507.222	265.025	406.258	0.878	Mann–Whitney U test
Initial vertical velocity (mm/s)	516.649	525.911	312.243	441.184	204.926	361.302	0.645	Mann–Whitney U test
Maximum acceleration (mm/s^2)	4.28E+04	2.94E+04	3.11E+04	4.22E+04	2.01E+04	3.51E+04	0.645	Mann–Whitney U test
Initial velocity (mm/s)	533.442	537.86	318.955	453.822	217.841	362.568	0.721	Mann–Whitney U test
Absolute initial velocity (mm/s)	533.442	537.86	318.955	453.822	217.841	362.568	0.721	Mann–Whitney U test
Maximum jerk (mm/s^3)	1.93E+06	1.30E+06	1.91E+06	2.73E+06	1.11E+06	3.09E+06	0.238	Student's t-test
Lag time (s)	10.315	10.599	5.847	5.731	2.289	5.615	0.721	Mann–Whitney U test
Event Angle (degrees)	92.3	7.613	92.134	90.419	1.222	90.129	0.529	Student's t-test

Supplementary Table S35. Comparing event counts and drop metrics of 4-week-old control males (n = 6) to 4-week-old *SNCA* males (n = 6). *p*-value for significance is $p < 0.05$.

	Control male			*SNCA* male				
**Metric**	**Mean**	**SD**	**Median**	**Mean**	**SD**	**Median**	***p* value**	**Test**
Event Counts								
Upward Jumps	0	0	0	0	0	0	-	Welch's t-test
Arcjumps	0.5	0.5	0.5	0.333	0.745	0	0.441	Mann–Whitney U test
Falls	6.333	6.446	5	4.5	3.354	4	1	Mann–Whitney U test
Downward Jumps	0	0	0	0.167	0.373	0	0.405	Mann–Whitney U test
Drops	6.333	6.446	5	4.667	3.249	4.5	1	Mann–Whitney U test
Total Events	6.833	6.669	5	5	3.162	5.5	1	Mann–Whitney U test
Total Movement (mm)	4.32E+03	2.44E+03	3.74E+03	1.49E+03	871.152	1.56E+03	0.049	Welch's t-test
Time spent in upper half of vial	0.473	0.128	0.433	0.438	0.171	0.456	0.727	Student's t-test
Event-Movement ratio	1.57E-03	8.51E-04	1.66E-03	3.31E-03	1.73E-03	3.55E-03	0.071	Student's t-test
Drop Metrics								
Event duration (frames)	3.964	1.382	4.417	3.884	1.47	4.5	1	Mann–Whitney U test
Event duration (s)	0.068	0.024	0.076	0.067	0.025	0.078	1	Mann–Whitney U test
Vertical displacement (mm)	20.862	8.753	23.784	23.078	9.531	25.873	0.725	Student's t-test
Horizontal displacement (mm)	4.078	2.875	4.011	3.825	1.641	3.752	0.878	Student's t-test
Estimated height at event trigger (mm)	15.656	9.735	11.679	14.162	9.648	10.965	0.823	Student's t-test
Estimated height at event start (mm)	14.263	9.068	11.231	12.181	9.76	8.215	0.748	Student's t-test
Estimated X position at event start (mm)	20.668	2.578	20.553	15.807	4.978	17.437	0.093	Student's t-test
Estimated height at event end (mm)	35.125	1.227	35.481	35.259	3.419	37.256	0.247	Mann–Whitney U test
Estimated X position at event end (mm)	21.923	2.117	21.754	13.426	3.27	13.379	1.13E-03	Student's t-test
Event velocity (mm/s)	297.98	71.151	310.406	696.364	670.233	491.307	0.126	Mann–Whitney U test
Maximum velocity (mm/s)	456.618	148.907	514.438	758.406	636.342	545.47	0.662	Mann–Whitney U test
Average velocity (mm/s)	303.165	71.775	316.868	620.07	697.109	330.417	1	Mann–Whitney U test
Initial vertical velocity (mm/s)	281.229	89.83	271.845	602.847	697.985	293.464	0.792	Mann–Whitney U test
Maximum acceleration (mm/s^2)	1.92E+04	6.08E+03	1.89E+04	3.89E+04	3.90E+04	2.47E+04	0.537	Mann–Whitney U test
Initial velocity (mm/s)	294.918	95.368	282.288	614.344	695.943	300.301	0.931	Mann–Whitney U test
Absolute initial velocity (mm/s)	294.918	95.368	282.288	614.344	695.943	300.301	0.931	Mann–Whitney U test
Maximum jerk (mm/s^3)	1.12E+06	5.82E+05	1.20E+06	1.00E+06	6.05E+05	1.13E+06	0.778	Student's t-test
Lag time (s)	11.932	10.771	6.862	24.55	15.354	17.795	0.126	Mann–Whitney U test
Event Angle (degrees)	88.895	9.952	90.643	91.553	11.086	94.97	0.429	Mann–Whitney U test

Supplementary Table S36. Comparing event counts and drop metrics of 2-week-old control males (n = 24) to 2-week-old *SNCA* males (n = 24). Pooled data from Tables S29-S31. p-value for significance is $p < 0.05$.

	**Control male**			***SNCA* male**				
**Metric**	**Mean**	**SD**	**Median**	**Mean**	**SD**	**Median**	***p* value**	**Test**
Event Counts								
Upward Jumps	0.083	0.276	0.000	0.042	0.200	0.000	0.572	Mann–Whitney U test
Arcjumps	0.083	0.276	0.000	0.125	0.331	0.000	0.654	Mann–Whitney U test
Falls	12.833	7.122	12.000	16.000	9.832	14.000	0.392	Mann–Whitney U test
Downward Jumps	0.917	1.256	0.500	0.625	0.857	0.000	0.516	Mann–Whitney U test
Drops	13.750	7.661	13.500	16.625	9.974	15.000	0.451	Mann–Whitney U test
Total Events	13.917	7.533	14.000	16.792	10.029	15.000	0.432	Mann–Whitney U test
Total Movement (mm)	3.88E+03	1.99E+03	3.91E+03	4.99E+03	2.37E+03	5.10E+03	0.091	Student's t-test
Time spent in upper half of vial	0.521	0.133	0.523	0.449	0.130	0.434	0.056	Mann–Whitney U test
Event-Movement ratio	3.94E-03	1.59E-03	3.92E-03	3.52E-03	1.85E-03	3.18E-03	0.413	Student's t-test
Drop Metrics								
Event duration (frames)	4.627	0.762	4.528	4.715	0.720	4.820	0.690	Student's t-test
Event duration (s)	0.080	0.013	0.078	0.081	0.012	0.083	0.690	Student's t-test
Vertical displacement (mm)	25.375	4.126	25.041	24.550	3.755	24.703	0.482	Student's t-test
Horizontal displacement (mm)	3.993	1.286	4.056	3.974	1.500	3.562	0.910	Mann–Whitney U test
Estimated height at event trigger (mm)	12.447	4.177	12.324	12.505	3.242	11.930	0.959	Student's t-test
Estimated height at event start (mm)	11.721	3.787	11.374	11.689	3.073	11.339	0.975	Student's t-test
Estimated X position at event start (mm)	20.657	2.266	20.804	21.406	2.453	20.526	0.288	Student's t-test
Estimated height at event end (mm)	37.096	3.095	37.717	36.240	3.127	36.556	0.356	Student's t-test
Estimated X position at event end (mm)	20.246	1.339	20.095	20.227	2.058	20.265	0.971	Student's t-test
Event velocity (mm/s)	502.696	169.657	453.834	464.553	128.574	441.450	0.490	Mann–Whitney U test
Maximum velocity (mm/s)	628.601	213.880	591.406	557.587	110.190	547.675	0.166	Welch's t-test
Average velocity (mm/s)	337.460	120.560	305.231	313.477	70.125	309.164	0.829	Mann–Whitney U test
Initial vertical velocity (mm/s)	304.360	107.359	277.712	281.627	89.084	259.864	0.427	Mann–Whitney U test
Maximum acceleration (mm/s^2)	2.93E+04	1.24E+04	2.73E+04	2.42E+04	7.14E+03	2.19E+04	0.095	Welch's t-test
Initial velocity (mm/s)	315.422	112.904	286.192	293.173	93.150	270.059	0.439	Mann–Whitney U test
Absolute initial velocity (mm/s)	315.422	112.904	286.192	293.173	93.150	270.059	0.439	Mann–Whitney U test
Maximum jerk (mm/s^3)	2.03E+06	1.38E+06	1.87E+06	1.52E+06	6.25E+05	1.39E+06	0.113	Welch's t-test
Lag time (s)	4.728	4.132	4.000	5.890	5.919	4.391	0.845	Mann–Whitney U test
Event Angle (degrees)	90.947	4.706	91.203	92.326	5.303	91.579	0.356	Student's t-test

Supplementary Table S37. Comparing event counts and drop metrics of 3-week-old control males (n = 20) to 3-week-old *SNCA* males (n = 24). Pooled data from Tables S32-S34. *p*-value for significance is $p < 0.05$.

	Control male			*SNCA* male				
**Metric**	**Mean**	**SD**	**Median**	**Mean**	**SD**	**Median**	***p* value**	**Test**
Event Counts								
Upward Jumps	0.150	0.357	0.000	0.083	0.276	0.000	0.506	Mann–Whitney U test
Arcjumps	0.250	0.622	0.000	0.125	0.599	0.000	0.257	Mann–Whitney U test
Falls	5.950	4.444	6.500	5.292	3.272	5.000	0.584	Student's t-test
Downward Jumps	0.400	0.583	0.000	0.625	1.111	0.000	0.739	Mann–Whitney U test
Drops	6.350	4.564	7.000	5.917	3.439	5.000	0.728	Student's t-test
Total Events	6.750	4.938	7.000	6.125	3.789	5.000	0.645	Student's t-test
Total Movement (mm)	2.04E+03	1.37E+03	2.01E+03	2.50E+03	1.67E+03	1.99E+03	0.377	Mann–Whitney U test
Time spent in upper half of vial	0.384	0.176	0.398	0.440	0.148	0.459	0.267	Student's t-test
Event-Movement ratio	3.32E-03	1.76E-03	3.08E-03	2.63E-03	9.10E-04	2.63E-03	0.109	Student's t-test
Drop Metrics								
Event duration (frames)	4.230	1.287	4.690	4.548	0.923	4.667	0.789	Mann–Whitney U test
Event duration (s)	0.073	0.022	0.081	0.078	0.016	0.080	0.839	Mann–Whitney U test
Vertical displacement (mm)	24.686	6.362	26.017	24.767	4.796	25.186	0.964	Student's t-test
Horizontal displacement (mm)	3.055	1.732	2.703	2.958	1.321	2.980	0.842	Student's t-test
Estimated height at event trigger (mm)	15.978	6.927	13.695	14.170	6.209	12.541	0.354	Mann–Whitney U test
Estimated height at event start (mm)	14.706	6.301	12.979	13.316	5.639	12.046	0.468	Student's t-test
Estimated X position at event start (mm)	20.007	2.639	20.906	20.418	2.373	20.738	0.608	Student's t-test
Estimated height at event end (mm)	39.392	1.879	39.236	38.083	3.132	38.392	0.107	Mann–Whitney U test
Estimated X position at event end (mm)	19.587	1.960	19.088	20.016	1.814	20.272	0.208	Mann–Whitney U test
Event velocity (mm/s)	526.357	387.891	401.175	435.698	178.249	401.626	0.602	Mann–Whitney U test
Maximum velocity (mm/s)	730.252	363.749	688.907	739.981	277.656	700.320	0.751	Mann–Whitney U test
Average velocity (mm/s)	434.042	379.163	337.362	386.662	190.668	327.673	0.790	Mann–Whitney U test
Initial vertical velocity (mm/s)	381.578	372.696	283.968	313.352	164.548	252.882	0.469	Mann–Whitney U test
Maximum acceleration (mm/s^2)	3.52E+04	2.19E+04	2.87E+04	3.39E+04	1.66E+04	2.96E+04	0.949	Mann–Whitney U test
Initial velocity (mm/s)	394.243	381.412	298.055	345.530	202.759	259.000	0.485	Mann–Whitney U test
Absolute initial velocity (mm/s)	394.243	381.412	298.055	345.530	202.759	259.000	0.485	Mann–Whitney U test
Maximum jerk (mm/s^3)	2.04E+06	1.23E+06	2.12E+06	2.49E+06	1.39E+06	2.51E+06	0.354	Mann–Whitney U test
Lag time (s)	10.549	8.927	8.709	7.501	8.673	5.366	0.128	Mann–Whitney U test
Event Angle (degrees)	92.088	6.544	92.074	91.678	3.708	90.959	0.367	Mann–Whitney U test

Supplementary Table S38. Comparing event counts and drop metrics of 2-week-old *SNCA* males (n = 24) to 3-week-old *SNCA* males (n = 24). Pooled data from Tables S29-S34. *p*-value for significance is $p < 0.05$.

	**2-week *SNCA* male**			**3-week *SNCA* male**				
**Metric**	**Mean**	**SD**	**Median**	**Mean**	**SD**	**Median**	***p* value**	**Test**
Event Counts								
Upward Jumps	0.042	0.200	0.000	0.083	0.276	0.000	0.572	Mann–Whitney U test
Arcjumps	0.125	0.331	0.000	0.125	0.599	0.000	0.344	Mann–Whitney U test
Falls	16.000	9.832	14.000	5.292	3.272	5.000	1.50E-04	Mann–Whitney U test
Downward Jumps	0.625	0.857	0.000	0.625	1.111	0.000	0.750	Mann–Whitney U test
Drops	16.625	9.974	15.000	5.917	3.439	5.000	1.77E-04	Mann–Whitney U test
Total Events	16.792	10.029	15.000	6.125	3.789	5.000	2.25E-04	Mann–Whitney U test
Total Movement (mm)	4.99E+03	2.37E+03	5.10E+03	2.50E+03	1.67E+03	1.99E+03	2.96E-04	Mann–Whitney U test
Time spent in upper half of vial	0.449	0.130	0.434	0.440	0.148	0.459	0.797	Mann–Whitney U test
Event-Movement ratio	3.52E-03	1.85E-03	3.18E-03	2.63E-03	9.10E-04	2.63E-03	0.045	Welch's t-test
Drop Metrics								
Event duration (frames)	4.715	0.720	4.820	4.548	0.923	4.667	0.497	Student's t-test
Event duration (s)	0.081	0.012	0.083	0.078	0.016	0.080	0.497	Student's t-test
Vertical displacement (mm)	24.550	3.755	24.703	24.767	4.796	25.186	0.865	Student's t-test
Horizontal displacement (mm)	3.974	1.500	3.562	2.958	1.321	2.980	0.017	Mann–Whitney U test
Estimated height at event trigger (mm)	12.505	3.242	11.930	14.170	6.209	12.541	0.571	Mann–Whitney U test
Estimated height at event start (mm)	11.689	3.073	11.339	13.316	5.639	12.046	0.233	Welch's t-test
Estimated X position at event start (mm)	21.406	2.453	20.526	20.418	2.373	20.738	0.171	Student's t-test
Estimated height at event end (mm)	36.240	3.127	36.556	38.083	3.132	38.392	0.038	Mann–Whitney U test
Estimated X position at event end (mm)	20.227	2.058	20.265	20.016	1.814	20.272	0.959	Mann–Whitney U test
Event velocity (mm/s)	464.553	128.574	441.450	435.698	178.249	401.626	0.288	Mann–Whitney U test
Maximum velocity (mm/s)	557.587	110.190	547.675	739.981	277.656	700.320	3.77E-03	Mann–Whitney U test
Average velocity (mm/s)	313.477	70.125	309.164	386.662	190.668	327.673	0.392	Mann–Whitney U test
Initial vertical velocity (mm/s)	281.627	89.084	259.864	313.352	164.548	252.882	0.797	Mann–Whitney U test
Maximum acceleration (mm/s^2)	2.42E+04	7.14E+03	2.19E+04	3.39E+04	1.66E+04	2.96E+04	0.015	Mann–Whitney U test
Initial velocity (mm/s)	293.173	93.150	270.059	345.530	202.759	259.000	0.877	Mann–Whitney U test
Absolute initial velocity (mm/s)	293.173	93.150	270.059	345.530	202.759	259.000	0.877	Mann–Whitney U test
Maximum jerk (mm/s^3)	1.52E+06	6.25E+05	1.39E+06	2.49E+06	1.39E+06	2.51E+06	5.21E-03	Mann–Whitney U test
Lag time (s)	5.890	5.919	4.391	7.501	8.673	5.366	0.197	Mann–Whitney U test
Event Angle (degrees)	92.326	5.303	91.579	91.678	3.708	90.959	0.797	Mann–Whitney U test

Supplementary Table S39. Comparing event counts and drop metrics of 2-week-old *w[1118]* males (n = 24) to 3-week-old *w[1118]* males (n = 20). Pooled data from Tables S29-S34. *p*-value for significance is $p < 0.05$.

	**2-week *w[1118]* male**			**3-week *w[1118]* male**				
**Metric**	**Mean**	**SD**	**Median**	**Mean**	**SD**	**Median**	***p* value**	**Test**
Event Counts								
Upward Jumps	0.083	0.276	0.000	0.150	0.357	0.000	0.506	Mann–Whitney U test
Arcjumps	0.083	0.276	0.000	0.250	0.622	0.000	0.454	Mann–Whitney U test
Falls	12.833	7.122	12.000	5.950	4.444	6.500	5.56E-04	Mann–Whitney U test
Downward Jumps	0.917	1.256	0.500	0.400	0.583	0.000	0.199	Mann–Whitney U test
Drops	13.750	7.661	13.500	6.350	4.564	7.000	5.62E-04	Mann–Whitney U test
Total Events	13.917	7.533	14.000	6.750	4.938	7.000	6.90E-04	Mann–Whitney U test
Total Movement (mm)	3.88E+03	1.99E+03	3.91E+03	2.04E+03	1.37E+03	2.01E+03	1.41E-03	Student's t-test
Time spent in upper half of vial	0.521	0.133	0.523	0.384	0.176	0.398	6.28E-03	Student's t-test
Event-Movement ratio	3.94E-03	1.59E-03	3.92E-03	3.32E-03	1.76E-03	3.08E-03	0.233	Student's t-test
Drop Metrics								
Event duration (frames)	4.627	0.762	4.528	4.230	1.287	4.690	0.593	Mann–Whitney U test
Event duration (s)	0.080	0.013	0.078	0.073	0.022	0.081	0.602	Mann–Whitney U test
Vertical displacement (mm)	25.375	4.126	25.041	24.686	6.362	26.017	0.700	Welch's t-test
Horizontal displacement (mm)	3.993	1.286	4.056	3.055	1.732	2.703	0.056	Student's t-test
Estimated height at event trigger (mm)	12.447	4.177	12.324	15.978	6.927	13.695	0.073	Welch's t-test
Estimated height at event start (mm)	11.721	3.787	11.374	14.706	6.301	12.979	0.070	Student's t-test
Estimated X position at event start (mm)	20.657	2.266	20.804	20.007	2.639	20.906	0.408	Student's t-test
Estimated height at event end (mm)	37.096	3.095	37.717	39.392	1.879	39.236	9.63E-03	Student's t-test
Estimated X position at event end (mm)	20.246	1.339	20.095	19.587	1.960	19.088	0.214	Student's t-test
Event velocity (mm/s)	502.696	169.657	453.834	526.357	387.891	401.175	0.191	Mann–Whitney U test
Maximum velocity (mm/s)	628.601	213.880	591.406	730.252	363.749	688.907	0.367	Mann–Whitney U test
Average velocity (mm/s)	337.460	120.560	305.231	434.042	379.163	337.362	0.191	Mann–Whitney U test
Initial vertical velocity (mm/s)	304.360	107.359	277.712	381.578	372.696	283.968	0.751	Mann–Whitney U test
Maximum acceleration (mm/s^2)	2.93E+04	1.24E+04	2.73E+04	3.52E+04	2.19E+04	2.87E+04	0.423	Mann–Whitney U test
Initial velocity (mm/s)	315.422	112.904	286.192	394.243	381.412	298.055	0.620	Mann–Whitney U test
Absolute initial velocity (mm/s)	315.422	112.904	286.192	394.243	381.412	298.055	0.620	Mann–Whitney U test
Maximum jerk (mm/s^3)	2.03E+06	1.38E+06	1.87E+06	2.04E+06	1.23E+06	2.12E+06	0.986	Student's t-test
Lag time (s)	4.728	4.132	4.000	10.549	8.927	8.709	2.24E-03	Mann–Whitney U test
Event Angle (degrees)	90.947	4.706	91.203	92.088	6.544	92.074	0.525	Student's t-test

Supplementary Table S40. Comparing event counts and drop metrics of 3-week-old *SNCA* males (n = 24) to 4-week-old *SNCA* males (n = 6). Pooled data from Tables S32-S35. *p*-value for significance is $p < 0.05$.

	3-week *SNCA* male			4-week *SNCA* male				
**Metric**	**Mean**	**SD**	**Median**	**Mean**	**SD**	**Median**	***p* value**	**Test**
Event Counts								
Upward Jumps	0.083	0.276	0.000	0.000	0.000	0.000	0.509	Mann–Whitney U test
Arcjumps	0.125	0.599	0.000	0.333	0.745	0.000	0.338	Mann–Whitney U test
Falls	5.292	3.272	5.000	4.500	3.354	4.000	0.614	Student's t-test
Downward Jumps	0.625	1.111	0.000	0.167	0.373	0.000	0.318	Mann–Whitney U test
Drops	5.917	3.439	5.000	4.667	3.249	4.500	0.443	Student's t-test
Total Events	6.125	3.789	5.000	5.000	3.162	5.500	0.522	Student's t-test
Total Movement (mm)	2.50E+03	1.67E+03	1.99E+03	1.49E+03	871.152	1.56E+03	0.230	Mann–Whitney U test
Time spent in upper half of vial	0.440	0.148	0.459	0.438	0.171	0.456	0.977	Student's t-test
Event-Movement ratio	2.63E-03	9.10E-04	2.63E-03	3.31E-03	1.73E-03	3.55E-03	0.207	Student's t-test
Drop Metrics								
Event duration (frames)	4.548	0.923	4.667	3.884	1.470	4.500	0.488	Mann–Whitney U test
Event duration (s)	0.078	0.016	0.080	0.067	0.025	0.078	0.544	Mann–Whitney U test
Vertical displacement (mm)	24.767	4.796	25.186	23.078	9.531	25.873	0.578	Student's t-test
Horizontal displacement (mm)	2.958	1.321	2.980	3.825	1.641	3.752	0.229	Student's t-test
Estimated height at event trigger (mm)	14.170	6.209	12.541	14.162	9.648	10.965	0.716	Mann–Whitney U test
Estimated height at event start (mm)	13.316	5.639	12.046	12.181	9.760	8.215	0.736	Student's t-test
Estimated X position at event start (mm)	20.418	2.373	20.738	15.807	4.978	17.437	5.36E-03	Student's t-test
Estimated height at event end (mm)	38.083	3.132	38.392	35.259	3.419	37.256	0.059	Mann–Whitney U test
Estimated X position at event end (mm)	20.016	1.814	20.272	13.426	3.270	13.379	1.18E-04	Mann–Whitney U test
Event velocity (mm/s)	435.698	178.249	401.626	696.364	670.233	491.307	0.758	Mann–Whitney U test
Maximum velocity (mm/s)	739.981	277.656	700.320	758.406	636.342	545.470	0.201	Mann–Whitney U test
Average velocity (mm/s)	386.662	190.668	327.673	620.070	697.109	330.417	1.000	Mann–Whitney U test
Initial vertical velocity (mm/s)	313.352	164.548	252.882	602.847	697.985	293.464	0.845	Mann–Whitney U test
Maximum acceleration (mm/s^2)	3.39E+04	1.66E+04	2.96E+04	3.89E+04	3.90E+04	2.47E+04	0.245	Mann–Whitney U test
Initial velocity (mm/s)	345.530	202.759	259.000	614.344	695.943	300.301	0.845	Mann–Whitney U test
Absolute initial velocity (mm/s)	345.530	202.759	259.000	614.344	695.943	300.301	0.845	Mann–Whitney U test
Maximum jerk (mm/s^3)	2.49E+06	1.39E+06	2.51E+06	1.00E+06	6.05E+05	1.13E+06	0.013	Mann–Whitney U test
Lag time (s)	7.501	8.673	5.366	24.550	15.354	17.795	1.40E-03	Mann–Whitney U test
Event Angle (degrees)	91.678	3.708	90.959	91.553	11.086	94.970	0.129	Mann–Whitney U test

Supplementary Tables S41. Comparing event counts and drop metrics of 3-week-old *w[1118]* males (n = 20) to 4-week-old *w[1118]* males (n = 6). Pooled data from Tables S32-S35. *p*-value for significance is $p < 0.05$.

	**3-week *w[1118]* male**			**4-week *w[1118]* male**				
**Metric**	**Mean**	**SD**	**Median**	**Mean**	**SD**	**Median**	***p* value**	**Test**
Event Counts								
Upward Jumps	0.150	0.357	0.000	0.000	0.000	0.000	0.350	Mann–Whitney U test
Arcjumps	0.250	0.622	0.000	0.500	0.500	0.500	0.148	Mann–Whitney U test
Falls	5.950	4.444	6.500	6.333	6.446	5.000	0.713	Mann–Whitney U test
Downward Jumps	0.400	0.583	0.000	0.000	0.000	0.000	0.107	Mann–Whitney U test
Drops	6.350	4.564	7.000	6.333	6.446	5.000	0.624	Mann–Whitney U test
Total Events	6.750	4.938	7.000	6.833	6.669	5.000	0.783	Mann–Whitney U test
Total Movement (mm)	2.04E+03	1.37E+03	2.01E+03	4.32E+03	2.44E+03	3.74E+03	0.092	Welch's t-test
Time spent in upper half of vial	0.384	0.176	0.398	0.473	0.128	0.433	0.283	Student's t-test
Event-Movement ratio	3.32E-03	1.76E-03	3.08E-03	1.57E-03	8.51E-04	1.66E-03	0.033	Student's t-test
Drop Metrics								
Event duration (frames)	4.230	1.287	4.690	3.964	1.382	4.417	0.593	Mann–Whitney U test
Event duration (s)	0.073	0.022	0.081	0.068	0.024	0.076	0.548	Mann–Whitney U test
Vertical displacement (mm)	24.686	6.362	26.017	20.862	8.753	23.784	0.282	Student's t-test
Horizontal displacement (mm)	3.055	1.732	2.703	4.078	2.875	4.011	0.328	Student's t-test
Estimated height at event trigger (mm)	15.978	6.927	13.695	15.656	9.735	11.679	0.933	Student's t-test
Estimated height at event start (mm)	14.706	6.301	12.979	14.263	9.068	11.231	0.900	Student's t-test
Estimated X position at event start (mm)	20.007	2.639	20.906	20.668	2.578	20.553	0.614	Student's t-test
Estimated height at event end (mm)	39.392	1.879	39.236	35.125	1.227	35.481	5.49E-05	Student's t-test
Estimated X position at event end (mm)	19.587	1.960	19.088	21.923	2.117	21.754	0.027	Student's t-test
Event velocity (mm/s)	526.357	387.891	401.175	297.980	71.151	310.406	5.77E-03	Mann–Whitney U test
Maximum velocity (mm/s)	730.252	363.749	688.907	456.618	148.907	514.438	0.018	Mann–Whitney U test
Average velocity (mm/s)	434.042	379.163	337.362	303.165	71.775	316.868	0.343	Mann–Whitney U test
Initial vertical velocity (mm/s)	381.578	372.696	283.968	281.229	89.830	271.845	0.494	Mann–Whitney U test
Maximum acceleration (mm/s^2)	3.52E+04	2.19E+04	2.87E+04	1.92E+04	6.08E+03	1.89E+04	0.015	Mann–Whitney U test
Initial velocity (mm/s)	394.243	381.412	298.055	294.918	95.368	282.288	0.673	Mann–Whitney U test
Absolute initial velocity (mm/s)	394.243	381.412	298.055	294.918	95.368	282.288	0.673	Mann–Whitney U test
Maximum jerk (mm/s^3)	2.04E+06	1.23E+06	2.12E+06	1.12E+06	5.82E+05	1.20E+06	0.106	Student's t-test
Lag time (s)	10.549	8.927	8.709	11.932	10.771	6.862	1.000	Mann–Whitney U test
Event Angle (degrees)	92.088	6.544	92.074	88.895	9.952	90.643	0.399	Student's t-test

Supplementary Table S42. Comparing event counts and drop metrics of 2-week-old *SNCA* males (n = 24) to 4-week-old *SNCA* males (n = 6). Pooled data from Tables S29-S31 and Table S35. *p*-value for significance is $p < 0.05$.

	2-week *SNCA* male			4-week *SNCA* male				
**Metric**	**Mean**	**SD**	**Median**	**Mean**	**SD**	**Median**	***p* value**	**Test**
Event Counts								
Upward Jumps	0.042	0.200	0.000	0.000	0.000	0.000	0.677	Mann–Whitney U test
Arcjumps	0.125	0.331	0.000	0.333	0.745	0.000	0.725	Mann–Whitney U test
Falls	16.000	9.832	14.000	4.500	3.354	4.000	9.43E-03	Mann–Whitney U test
Downward Jumps	0.625	0.857	0.000	0.167	0.373	0.000	0.238	Mann–Whitney U test
Drops	16.625	9.974	15.000	4.667	3.249	4.500	8.67E-03	Mann–Whitney U test
Total Events	16.792	10.029	15.000	5.000	3.162	5.500	0.013	Mann–Whitney U test
Total Movement (mm)	4.99E+03	2.37E+03	5.10E+03	1.49E+03	871.152	1.56E+03	1.88E-03	Student's t-test
Time spent in upper half of vial	0.449	0.130	0.434	0.438	0.171	0.456	0.781	Mann–Whitney U test
Event-Movement ratio	3.52E-03	1.85E-03	3.18E-03	3.31E-03	1.73E-03	3.55E-03	0.807	Student's t-test
Drop Metrics								
Event duration (frames)	4.715	0.720	4.820	3.884	1.470	4.500	0.272	Mann–Whitney U test
Event duration (s)	0.081	0.012	0.083	0.067	0.025	0.078	0.295	Mann–Whitney U test
Vertical displacement (mm)	24.550	3.755	24.703	23.078	9.531	25.873	0.775	Welch's t-test
Horizontal displacement (mm)	3.974	1.500	3.562	3.825	1.641	3.752	1.000	Mann–Whitney U test
Estimated height at event trigger (mm)	12.505	3.242	11.930	14.162	9.648	10.965	0.519	Student's t-test
Estimated height at event start (mm)	11.689	3.073	11.339	12.181	9.760	8.215	0.846	Student's t-test
Estimated X position at event start (mm)	21.406	2.453	20.526	15.807	4.978	17.437	1.22E-03	Student's t-test
Estimated height at event end (mm)	36.240	3.127	36.556	35.259	3.419	37.256	0.758	Mann–Whitney U test
Estimated X position at event end (mm)	20.227	2.058	20.265	13.426	3.270	13.379	3.86E-06	Student's t-test
Event velocity (mm/s)	464.553	128.574	441.450	696.364	670.233	491.307	0.845	Mann–Whitney U test
Maximum velocity (mm/s)	557.587	110.190	547.675	758.406	636.342	545.470	0.716	Mann–Whitney U test
Average velocity (mm/s)	313.477	70.125	309.164	620.070	697.109	330.417	0.245	Mann–Whitney U test
Initial vertical velocity (mm/s)	281.627	89.084	259.864	602.847	697.985	293.464	0.674	Mann–Whitney U test
Maximum acceleration (mm/s^2)	2.42E+04	7.14E+03	2.19E+04	3.89E+04	3.90E+04	2.47E+04	0.758	Mann–Whitney U test
Initial velocity (mm/s)	293.173	93.150	270.059	614.344	695.943	300.301	0.556	Mann–Whitney U test
Absolute initial velocity (mm/s)	293.173	93.150	270.059	614.344	695.943	300.301	0.556	Mann–Whitney U test
Maximum jerk (mm/s^3)	1.52E+06	6.25E+05	1.39E+06	1.00E+06	6.05E+05	1.13E+06	0.118	Student's t-test
Lag time (s)	5.890	5.919	4.391	24.550	15.354	17.795	1.90E-03	Mann–Whitney U test
Event Angle (degrees)	92.326	5.303	91.579	91.553	11.086	94.970	0.295	Mann–Whitney U test

Supplementary Table S43. Comparing event counts and drop metrics of 2-week-old *w[1118]* males (n = 24) to 4-week-old *w[1118]* males (n = 6). Pooled data from Tables S29-S31 and Table S35. *p*-value for significance is $p < 0.05$.

	2-week *w[1118]* male			4-week *w[1118]* male				
**Metric**	**Mean**	**SD**	**Median**	**Mean**	**SD**	**Median**	***p* value**	**Test**
Event Counts								
Upward Jumps	0.083	0.276	0.000	0.000	0.000	0.000	0.509	Mann–Whitney U test
Arcjumps	0.083	0.276	0.000	0.500	0.500	0.500	0.018	Mann–Whitney U test
Falls	12.833	7.122	12.000	6.333	6.446	5.000	0.019	Mann–Whitney U test
Downward Jumps	0.917	1.256	0.500	0.000	0.000	0.000	0.036	Mann–Whitney U test
Drops	13.750	7.661	13.500	6.333	6.446	5.000	0.019	Mann–Whitney U test
Total Events	13.917	7.533	14.000	6.833	6.669	5.000	0.018	Mann–Whitney U test
Total Movement (mm)	3.88E+03	1.99E+03	3.91E+03	4.32E+03	2.44E+03	3.74E+03	0.657	Student's t-test
Time spent in upper half of vial	0.521	0.133	0.523	0.473	0.128	0.433	0.440	Student's t-test
Event-Movement ratio	3.94E-03	1.59E-03	3.92E-03	1.57E-03	8.51E-04	1.66E-03	1.99E-03	Student's t-test
Drop Metrics								
Event duration (frames)	4.627	0.762	4.528	3.964	1.382	4.417	0.500	Mann–Whitney U test
Event duration (s)	0.080	0.013	0.078	0.068	0.024	0.076	0.484	Mann–Whitney U test
Vertical displacement (mm)	25.375	4.126	25.041	20.862	8.753	23.784	0.087	Student's t-test
Horizontal displacement (mm)	3.993	1.286	4.056	4.078	2.875	4.011	0.951	Welch's t-test
Estimated height at event trigger (mm)	12.447	4.177	12.324	15.656	9.735	11.679	0.500	Welch's t-test
Estimated height at event start (mm)	11.721	3.787	11.374	14.263	9.068	11.231	0.563	Welch's t-test
Estimated X position at event start (mm)	20.657	2.266	20.804	20.668	2.578	20.553	0.992	Student's t-test
Estimated height at event end (mm)	37.096	3.095	37.717	35.125	1.227	35.481	0.151	Student's t-test
Estimated X position at event end (mm)	20.246	1.339	20.095	21.923	2.117	21.754	0.028	Student's t-test
Event velocity (mm/s)	502.696	169.657	453.834	297.980	71.151	310.406	1.00E-03	Mann–Whitney U test
Maximum velocity (mm/s)	628.601	213.880	591.406	456.618	148.907	514.438	0.083	Student's t-test
Average velocity (mm/s)	337.460	120.560	305.231	303.165	71.775	316.868	0.940	Mann–Whitney U test
Initial vertical velocity (mm/s)	304.360	107.359	277.712	281.229	89.830	271.845	0.667	Mann–Whitney U test
Maximum acceleration (mm/s^2)	2.93E+04	1.24E+04	2.73E+04	1.92E+04	6.08E+03	1.89E+04	0.071	Student's t-test
Initial velocity (mm/s)	315.422	112.904	286.192	294.918	95.368	282.288	0.667	Mann–Whitney U test
Absolute initial velocity (mm/s)	315.422	112.904	286.192	294.918	95.368	282.288	0.667	Mann–Whitney U test
Maximum jerk (mm/s^3)	2.03E+06	1.38E+06	1.87E+06	1.12E+06	5.82E+05	1.20E+06	0.137	Student's t-test
Lag time (s)	4.728	4.132	4.000	11.932	10.771	6.862	0.057	Mann–Whitney U test
Event Angle (degrees)	90.947	4.706	91.203	88.895	9.952	90.643	0.484	Student's t-test

Supplementary Table S44. Kruskal-Wallis test comparing drop metrics between HDBSCAN-defined clusters.

**Metric**	**H statistic**	ε^2	**Bonferroni-adjusted *p*-value**
Event duration (frames)	469.325	0.203	3.81E-100
Event duration (s)	451.438	0.195	2.86E-96
Vertical displacement (mm)	781.291	0.339	8.89E-168
Horizontal displacement (mm)	6.305	0.001	1.00
Estimated height at event trigger (mm)	1000.886	0.435	2.08E-215
Estimated height at event start (mm)	1005.992	0.437	1.62E-216
Estimated X position at event start (mm)	0.341	-0.001	1.00
Estimated height at event end (mm)	61.630	0.026	4.74E-12
Estimated X position at event end (mm)	5.539	0.001	1.00
Event velocity (mm/s)	646.363	0.280	1.61E-138
Maximum velocity (mm/s)	805.299	0.350	5.52E-173
Average velocity (mm/s)	550.939	0.239	7.82E-118
Initial vertical velocity (mm/s)	435.433	0.188	8.41E-93
Maximum acceleration (mm/s^2)	550.068	0.238	1.21E-117
Initial velocity (mm/s)	443.035	0.192	1.90E-94
Absolute initial velocity (mm/s)	443.035	0.192	1.90E-94
Maximum jerk (mm/s^3)	343.761	0.148	6.02E-73
Event angle	5.229	0.001	1.00

Supplementary Table S45. Post-Hoc Dunn's Test comparing drop metrics between HDBSCAN-derived clusters.

Metric	First cluster	Second cluster	Cliff's δ	*p* value
Event duration (frames)	0	1	-0.201	1.00
Event duration (frames)	0	2	0.089	9.44E-03
Event duration (frames)	0	3	-0.433	9.51E-21
Event duration (frames)	1	2	0.471	1.11E-04
Event duration (frames)	1	3	-0.431	3.30E-10
Event duration (frames)	2	3	-0.626	2.66E-89
Event duration (s)	0	1	-0.191	1.00
Event duration (s)	0	2	0.093	7.63E-03
Event duration (s)	0	3	-0.424	1.41E-19
Event duration (s)	1	2	0.465	9.54E-05
Event duration (s)	1	3	-0.419	1.42E-09
Event duration (s)	2	3	-0.622	2.01E-86
Vertical displacement (mm)	0	1	-0.051	1.00
Vertical displacement (mm)	0	2	0.937	1.33E-57
Vertical displacement (mm)	0	3	0	1.00
Vertical displacement (mm)	1	2	0.968	3.02E-49
Vertical displacement (mm)	1	3	0.041	1.00
Vertical displacement (mm)	2	3	-0.871	6.39E-165
Horizontal displacement (mm)	0	1	0.146	0.259
Horizontal displacement (mm)	0	2	0.003	1.00
Horizontal displacement (mm)	0	3	0.039	1.00
Horizontal displacement (mm)	1	2	-0.167	0.107
Horizontal displacement (mm)	1	3	-0.103	0.362
Horizontal displacement (mm)	2	3	0.039	1.00
Estimated height at event trigger (mm)	0	1	0.203	0.0503
Estimated height at event trigger (mm)	0	2	0.957	1.65E-82
Estimated height at event trigger (mm)	0	3	0.097	9.19E-01
Estimated height at event trigger (mm)	1	2	1	1.10E-43
Estimated height at event trigger (mm)	1	3	-0.152	0.203
Estimated height at event trigger (mm)	2	3	-0.998	4.57E-212
Estimated height at event start (mm)	0	1	0.193	0.062
Estimated height at event start (mm)	0	2	0.974	5.45E-76
Estimated height at event start (mm)	0	3	0.03	1.00
Estimated height at event start (mm)	1	2	1	5.09E-40
Estimated height at event start (mm)	1	3	-0.209	1.99E-02
Estimated height at event start (mm)	2	3	-0.998	1.28E-215
Estimated X position at event start (mm)	0	1	-0.001	1.00
Estimated X position at event start (mm)	0	2	0.001	1.00
Estimated X position at event start (mm)	0	3	-0.016	1.00
Estimated X position at event start (mm)	1	2	-0.005	1.00
Estimated X position at event start (mm)	1	3	-0.018	1.00
Estimated X position at event start (mm)	2	3	-0.015	1.00
Estimated height at event end (mm)	0	1	0.099	0.915
Estimated height at event end (mm)	0	2	0.221	5.34E-04
Estimated height at event end (mm)	0	3	-0.031	1.00
Estimated height at event end (mm)	1	2	0.108	0.441
Estimated height at event end (mm)	1	3	-0.134	0.116
Estimated height at event end (mm)	2	3	-0.246	8.67E-14
Estimated X position at event end (mm)	0	1	0.136	0.324
Estimated X position at event end (mm)	0	2	0	1.00
Estimated X position at event end (mm)	0	3	0.015	1.00
Estimated X position at event end (mm)	1	2	-0.147	0.135
Estimated X position at event end (mm)	1	3	-0.125	0.172
Estimated X position at event end (mm)	2	3	0.017	1.00
Event velocity (mm/s)	0	1	-0.742	2.69E-14
Event velocity (mm/s)	0	2	0.745	6.00E-54
Event velocity (mm/s)	0	3	0.375	5.68E-11
Event velocity (mm/s)	1	2	0.997	3.55E-115
Event velocity (mm/s)	1	3	0.999	1.59E-55
Event velocity (mm/s)	2	3	-0.563	1.93E-59
Maximum velocity (mm/s)	0	1	1	6.29E-111
Maximum velocity (mm/s)	0	2	1	6.22E-130
Maximum velocity (mm/s)	0	3	0.953	2.77E-62
Maximum velocity (mm/s)	1	2	-0.439	1.43E-05

Maximum velocity (mm/s)	1	3	-0.833	1.42E-44
Maximum velocity (mm/s)	2	3	-0.557	9.56E-58
Average velocity (mm/s)	0	1	0.962	1.58E-64
Average velocity (mm/s)	0	2	0.948	9.96E-105
Average velocity (mm/s)	0	3	0.855	1.21E-54
Average velocity (mm/s)	1	2	-0.009	1.00
Average velocity (mm/s)	1	3	-0.505	1.30E-15
Average velocity (mm/s)	2	3	-0.452	1.53E-40
Initial vertical velocity (mm/s)	0	1	0.993	1.08E-56
Initial vertical velocity (mm/s)	0	2	0.99	8.49E-84
Initial vertical velocity (mm/s)	0	3	0.988	5.02E-83
Initial vertical velocity (mm/s)	1	2	-0.08	0.911
Initial vertical velocity (mm/s)	1	3	-0.227	1.63E-03
Initial vertical velocity (mm/s)	2	3	-0.126	1.15E-03
Maximum acceleration (mm/s^2)	0	1	1	1.65E-81
Maximum acceleration (mm/s^2)	0	2	1	6.30E-103
Maximum acceleration (mm/s^2)	0	3	0.964	4.74E-72
Maximum acceleration (mm/s^2)	1	2	-0.278	5.39E-03
Maximum acceleration (mm/s^2)	1	3	-0.53	3.12E-18
Maximum acceleration (mm/s^2)	2	3	-0.326	1.41E-20
Initial velocity (mm/s)	0	1	1	2.14E-59
Initial velocity (mm/s)	0	2	1	7.31E-84
Initial velocity (mm/s)	0	3	0.996	1.26E-84
Initial velocity (mm/s)	1	2	-0.114	0.366
Initial velocity (mm/s)	1	3	-0.246	4.39E-04
Initial velocity (mm/s)	2	3	-0.117	3.34E-03
Absolute initial velocity (mm/s)	0	1	1	2.14E-59
Absolute initial velocity (mm/s)	0	2	1	7.31E-84
Absolute initial velocity (mm/s)	0	3	0.996	1.26E-84
Absolute initial velocity (mm/s)	1	2	-0.114	0.366
Absolute initial velocity (mm/s)	1	3	-0.246	4.39E-04
Absolute initial velocity (mm/s)	2	3	-0.117	3.34E-03
Maximum jerk (mm/s^3)	0	1	0.707	2.31E-63
Maximum jerk (mm/s^3)	0	2	0.446	7.37E-21
Maximum jerk (mm/s^3)	0	3	0.375	4.46E-10
Maximum jerk (mm/s^3)	1	2	-0.84	9.66E-29
Maximum jerk (mm/s^3)	1	3	-0.924	4.73E-56
Maximum jerk (mm/s^3)	2	3	-0.222	6.82E-10
Event angle (degrees)	0	1	0.137	0.500
Event angle (degrees)	0	2	0.079	0.813
Event angle (degrees)	0	3	0.035	1.00
Event angle (degrees)	1	2	-0.026	1.00
Event angle (degrees)	1	3	-0.094	0.674
Event angle (degrees)	2	3	-0.046	0.837

Supplementary Table S46. Comparison of manually-annotated events and events detected by MDv2.1 on reference video.

Manual annotation		MDv2.1 scoring	
Timestamp	Classification	Timestamp	Classification
0:00:24	drop	0:00:23	drop
0:00:25	upjump	0:00:25	upjump
0:00:35	drop	0:00:34	drop
0:00:50	drop	0:00:50	drop
0:01:39	drop	0:01:39	drop
0:01:44	arcjump	0:01:43	arcjump
0:01:46	upjump	0:01:47	upjump
0:01:53	drop	0:01:53	drop
0:02:04	drop	0:02:04	drop
0:02:09	upjump	0:02:09	upjump
0:02:11	drop	0:02:11	drop
0:02:14	upjump	0:02:14	upjump
0:02:18	drop	0:02:18	drop
0:02:27	drop	0:02:27	drop
0:02:34	drop	0:02:33	drop
0:02:44	arcjump	0:02:44	arcjump
0:02:50	drop	0:02:50	drop
0:02:53	upjump	0:02:53	upjump
0:03:06	drop	0:03:06	drop
0:03:13	arcjump	0:03:12	arcjump
0:03:18	drop	0:03:18	drop
0:03:50	drop	0:03:49	drop
0:04:22	arcjump	0:04:23	arcjump
0:04:26	arcjump	0:04:26	arcjump
0:04:28	upjump	0:04:28	upjump
0:08:35	drop	0:08:35	drop
0:09:12	drop	0:09:11	drop
0:10:58	drop	0:10:57	drop
0:11:35	drop	0:11:35	drop
0:11:50	drop	0:11:50	drop
0:11:54	drop	0:11:54	drop
0:12:08	drop	0:12:08	drop
0:12:23	drop	0:12:23	drop
0:13:06	drop	0:13:06	drop
0:13:14	drop	0:13:14	drop
0:14:08	arcjump	0:14:08	arcjump
0:14:20	drop	0:14:19	drop
0:14:52	drop	0:14:52	drop
0:15:19	drop	0:15:18	drop
0:17:34	drop	0:17:34	drop
0:18:03	drop	0:18:03	drop
0:18:58	drop	0:18:57	drop
0:19:58	drop	0:19:57	drop
0:22:49	drop	0:22:49	drop

Supplementary Table S47. Comparison of manually-annotated events and events detected by MDv2.1 on novel data.

Manual annotation		MDv2.1 scoring	
Timestamp	Classification	Timestamp	Classification
0:01:08	upjump	-	-
0:01:13	drop	0:01:13	drop
0:02:07	drop	-	-
-	-	0:02:34	drop
-	-	0:02:38	drop
-	-	0:04:08	drop
-	-	0:04:36	drop
0:05:28	drop	0:05:27	drop
0:06:07	upjump	-	-
0:06:12	drop	0:06:12	drop
0:06:27	drop	0:06:26	drop
0:07:09	arcjump	0:07:09	arcjump
0:07:51	drop	0:07:50	drop
0:11:28	drop	0:11:28	drop
0:12:57	drop	0:12:57	drop
0:14:02	drop	0:14:02	drop
0:28.21	drop	0:28:21	drop

Supplementary Table S48. Comparing drop metrics of 2 day old *w[1118]* males (n = 6) to 2 day old dehydrated *w[1118]* males (n = 7). Pooled data from Supplementary Tables S13-S14. *p*-value for significance is $p < 0.05$.

	***w[1118]* male**			**Dehydrated *w[1118]* male**				
**Metric**	**Mean**	**SD**	**Median**	**Mean**	**SD**	**Median**	***p* value**	**Test**
Drop Metrics								
Event duration (frames)	4.245	1.822	4.450	4.573	0.676	4.388	1.000	Mann–Whitney U test
Event duration (s)	0.073	0.031	0.077	0.079	0.012	0.076	1.000	Mann–Whitney U test
Vertical displacement (mm)	28.749	4.202	28.962	23.722	3.102	23.994	0.069	Student's t-test
Horizontal displacement (mm)	2.301	1.065	2.720	3.724	1.301	2.942	0.429	Mann–Whitney U test
Estimated height at event trigger (mm)	9.070	2.639	7.886	12.171	6.099	11.716	0.340	Welch's t-test
Estimated height at event start (mm)	8.208	2.457	6.508	10.852	5.375	10.190	0.662	Mann–Whitney U test
Estimated X position at event start (mm)	22.406	1.877	23.358	20.725	2.857	22.061	0.334	Student's t-test
Estimated height at event end (mm)	36.957	2.155	36.879	34.573	2.576	34.805	0.171	Student's t-test
Estimated X position at event end (mm)	22.214	1.200	21.589	22.058	1.323	21.736	0.858	Student's t-test
Event velocity (mm/s)	681.466	622.451	416.640	411.129	129.527	433.969	0.931	Mann–Whitney U test
Maximum velocity (mm/s)	903.281	514.335	694.303	740.686	329.148	665.394	0.792	Mann–Whitney U test
Average velocity (mm/s)	647.289	637.362	348.849	446.808	177.512	475.015	0.931	Mann–Whitney U test
Initial vertical velocity (mm/s)	573.530	674.509	265.232	387.898	155.943	413.107	0.662	Mann–Whitney U test
Maximum acceleration (mm/s^2)	4.47E+04	3.36E+04	2.90E+04	3.60E+04	2.08E+04	3.05E+04	0.931	Mann–Whitney U test
Initial velocity (mm/s)	582.625	669.979	275.962	461.431	229.101	463.574	0.662	Mann–Whitney U test
Absolute initial velocity (mm/s)	582.625	669.979	275.962	461.431	229.101	463.574	0.662	Mann–Whitney U test
Maximum jerk (mm/s^3)	1.77E+06	1.00E+06	2.07E+06	2.57E+06	2.35E+06	1.60E+06	0.931	Mann–Whitney U test
Lag time (s)	2.204	2.408	1.119	1.030	0.406	0.847	0.429	Mann–Whitney U test
Event Angle (degrees)	88.802	2.495	88.300	86.412	4.059	87.998	0.326	Student's t-test
